# Estimating the prevalence of adults at risk for advanced hepatic fibrosis using FIB-4 in a Swiss tertiary care hospital

**DOI:** 10.1371/journal.pone.0317629

**Published:** 2025-01-24

**Authors:** Petra Strajhar, Annalisa Berzigotti, Henning Nilius, Michael Nagler, Jean-François Dufour

**Affiliations:** 1 Master of Public Health, University Basel, University Bern & University Zurich, Zurich, Switzerland; 2 Department of Visceral Surgery and Medicine, Inselspital, Bern University Hospital, University of Bern, Bern, Switzerland; 3 Department of Clinical Chemistry, Inselspital, Bern University Hospital, University of Bern, Bern, Switzerland; 4 Center for Digestive Diseases Lausanne, Lausanne, Switzerland; Medizinische Fakultat der RWTH Aachen, GERMANY

## Abstract

**Background & aims:**

Chronic liver diseases pose a serious public health issue. Identifying patients at risk for advanced liver fibrosis is crucial for early intervention. The Fibrosis-4 score (FIB-4), a simple non-invasive test, classifies patients into three risk groups for advanced fibrosis. This study aimed to estimate the prevalence of patients at risk for advanced hepatic fibrosis at a Swiss tertiary care hospital by calculating the FIB-4 score in routine blood analysis.

**Methods:**

A retrospective study was conducted using data from 36,360 patients who visited outpatient clinics at eight main clinics of the University Hospital Bern in Switzerland. The data collection period ran from January 1st to December 31st, 2022. Patients attending the hepatology outpatient clinic were excluded. We then calculated the overall and clinic-specific prevalence of patients falling into the high risk category for advanced fibrosis according to FIB-4.

**Results:**

Among the 36,360 patients, 26,245 (72.2%) had a low risk of advanced fibrosis (FIB-4 <1.3), whereas 3913 (10.8%) and 2597 (7.1%) patients were flagged to have a high risk of advanced fibrosis (FIB-4 >2.67 and FIB-4 >3.25 respectively). Geriatrics and Cardiology had the highest proportions of patients at risk for advanced fibrosis over all clinics.

**Conclusions:**

This study demonstrates a high prevalence of high FIB-4 score in a Swiss tertiary care hospital. The implementation of the automatically generated FIB-4 score in daily practice, not only in primary care, but also within tertiary care hospitals, could be crucial for early identification of outpatients at high risk of advanced liver fibrosis requiring further hepatological investigations.

## Introduction

Early diagnosis of advanced chronic liver disease [1–3], characterized by severe fibrosis [4, 5], is key to start treatment to prevent progression to the decompensated stage of cirrhosis, which carries a high morbidity and mortality risk [6]. Among the available non-invasive tests, the Fibrosis-4 score (FIB-4)—a simple score calculated from age, aspartate aminotransferase (ASAT), alanine aminotransferase (ALAT), and platelet count—has shown high diagnostic accuracy for rule-out advanced liver fibrosis and to identify patients at high risk of this complication in several etiologies of liver disease [7–9]. The current guidelines issued by the major international scientific associations in hepatology, as well as by the American and European endocrinology scientific societies, currently suggest to use FIB-4 score as a screening tool in individuals carrying risk factors for chronic liver disease (CLD) in primary care [7, 10–14]. The use of this score in settings other than primary care, such as endocrinology or cardiology clinics, where most patients with risk factors for metabolic dysfunction-associated liver disease are followed-up, is still debated [15–18]. Despite cost-benefit analyses on internal referring processes for suspected advanced fibrosis have been attempted, there is no data regarding the expected prevalence of negative and positive FIB-4 testing in the setting of tertiary care. This study hence aims to estimate what is the contemporary prevalence of FIB-4-based suspected advanced fibrosis in the outpatients setting in a large teaching hospital in Switzerland.

## Materials and methods

### Study design

This cross-sectional study used the results of previously analyzed patients’ blood and serum samples from the Insel Gruppe (encompassing the Inselspital, University Hospital Bern, four regional hospitals and two nursing homes) in Switzerland. The sample size included 36,360 adult individual subjects attending the outpatient clinics of 26 different clinics of the University Hospital Bern in Switzerland, regional hospitals and nursing homes during the time period from 1 January to 31 December 2022. The clinics were grouped into 8 main clinics as following: 1) Geriatrics (secondary hospital) encompassing secondary hospitals specialized in geriatrics, 2) Cardiology, 3) Secondary Hospital (encompasses secondary hospitals with heterogenous patient cohorts), 4) Emergency department, 5) Medicine (encompasses Ophthalmology, Infectiology, Hematology and Central hematological Laboratory, Pneumology & Allergology, Medical and radio oncology & Nuclear medicine, Anesthesiology and Pain management, Nephrology & Hypertension, Urology, Otorhinolaryngology, Osteoporosis, General internal medicine, Dermatology & Venereology, Rheumatology & Immunology, Neurology & Neurosurgery and Obstetrics and Gynaecology), 6) Surgery (encompasses Angiology & Vascular surgery, Visceral surgery and medicine, Orthopaedic surgery and Thoracic surgery), 7) Diabetology (encompassing Diabetology, Endocrinology, Nutritional medicine & Metabolism) and 8) Psychosomatic medicine. The structure of the Swiss health care system and the “Insel Gruppe” in Switzerland in the year of 2022 is explained in Text A in S1 Appendix.

The inclusion criteria were age ≥18 years, signed general consent of the University Hospital Bern and laboratory results of ASAT, ALAT, and platelet count. The exclusion criteria were laboratory results of ALAT ≥400 IU/L (to avoid elevated FIB-4 scores due to an artifact resulting from by acute liver disease) [19, 20] and subjects in follow-up at the hepatology outpatient clinic of the department of visceral surgery and medicine, since this clinic would per definition have a high prevalence of advanced liver fibrosis, and is not representative of the general population in tertiary care. Only the results of the first taken laboratory sample were used in cases several samples were available for the same subject. The coded data set of 36,360 patients included ASAT (IU/L), ALAT (IU/L), platelet count (G/L), age (in years), sex (female; male) and name of the clinic requiring the laboratory tests; glycated hemoglobin (HbA1c) (mmol/mol) and total cholesterol (mmol/L) where collected as non-mandatory tests, and were available in 12,229 patients and 12,649 patients respectively. Some authors had access to identifiable information during and after data collection. The data were accessed for research purposes between January 3 and February 15, 2024. The FIB-4 score was calculated using the following equation: $\text{FIB}-4\;\text{score}=\frac{\left(age*AST\right)}{\text{platelet}\;\text{count}*\surd ALT}$ where age is expressed in years, ALAT and ASAT in IU/L, and platelet count in G/L.

Two cutoffs, with age correction, were used to stratify the risk of advanced liver fibrosis as follows [2, 14, 21–23].

- <1.3 (or <2.0 for patients ≥65 years old): low risk
- ≥1.3 (or ≥2.0 for patients ≥65 years old)—≤2.67: indeterminate risk I
- ≥1.3 (or ≥2.0 for patients ≥65 years old)—≤3.25: indeterminate risk II
- >2.67: high risk
- >3.25: very high risk

The use of FIB-4 >2.67, which is less stringent and more sensitive compared to a FIB-4 score of >3.25 but with comparable specificity [24].

HbA1c levels below 39 mmol/mol, between 39 and 47 mmol/mol and ≥48 mmol/mol were classified as normal, prediabetes and diabetes respectively [25]. Total cholesterol levels below 5.2 mmol/L, between 5.2 and 6.19 mmol/L and ≥6.2 mmol/L were classified as healthy, borderline high and high levels respectively [26]. The upper limit of normal for both ASAT and ALAT is 50 IU/L at the University Hospital Bern.

The study was conducted in accordance with the regulatory requirements of the Human Research Act (HRA) as well as the Human Research Ordinance (HRO) and was approved by the Ethics Committee Bern Switzerland (Kantonale Ethikkommission Bern; Project ID 2023–02161). All of the subjects in the study provided a signed general consent (written consent) of the University Hospital Bern previous to this analysis.

### Statistical analyses

Continuous variables were expressed as mean, median, standard deviation (SD) and range whereas categorical variables were described as frequencies and percentages. The analysis of variance (ANOVA) and Bonferroni posthoc test were utilized to compare the means of patient’s clinical laboratory parameters and age between the risk strata for advanced hepatic fibrosis based on the FIB-4 score as well as to compare the means of FIB-4 score between the different clinics. Chi-square tests were performed to compare proportions. The threshold for statistical significance was set at a two-sided alpha value of 0.05 and statistical analyses were performed using the software package “STATA” version 16.1.

## Results

The patient cohort of 36,360 patients had a mean age of 55.1 years, 51.3% were females and had mean laboratory values of platelet count (249.5 G/L), ASAT (29.4 IU/L), ALAT (28.3 IU/L) and total cholesterol (4.6 mmol/L) in normal ranges, except for HbA1c (40.2 mmol/mol) indicating prediabetes. Most of the patients were classified into the age group of 35–64 years as well as group of normal HbA1c (<39 mmol/mol) and total cholesterol levels (<5.2 mmol/L) (Table 1). Of the 4129 patients aged 80 years or older (11%), 547 (13.2%) had a platelet count below 150 G/L, and 3879 (94%) had ASAT/ALAT above 0.8. 516 patients (12.5%) had both a low platelet count and elevated ASAT/ALAT. 6848 patients (18%) were younger than 35 years. The patient populations among the clinics are heterogeneous on their patient characteristics and cohort sizes. The clinics Medicine, Secondary hospitals and Emergency department had the biggest patient cohorts accounting for 41%, 24% and 19% of the whole patient sample size, whereas Geriatrics (secondary hospital) and Psychosomatic had the smallest patient cohorts (<1% of the whole patient sample size). In all clinics there were more females than males, except for Cardiology (24% females) and Emergency department (45% females). The lowest mean age was shown for Psychosomatic medicine (44 years), Diabetology (49 years) and Surgery (49 years) whereas the highest for Geriatrics (secondary hospital) (78 years) and Cardiology (60 years). Among all clinics Emergency department (22%) and Surgery (27%) had the highest proportion of patients in the youngest age group (18–34 years old), whereas Geriatrics (secondary hospital) (85%) and Cardiology (46%) the highest proportion of patients in the oldest age group (≥65 years).

**Table 1:** Cohort characteristics.

	Overall	Geriatrics (secondary hospital)	Cardiology	Secondary hospital^†^	Emergency department	Medicine^‡^	Surgery^§^	Diabetology	Psycho-somatic medicine
**N or n (%)**	**36,360**	168 (0.46)	1,182 (3.25)	8,1798 (24.2)	6,1725 (18.5)	14,1877 (40.92)	1,1777 (4.89)	2,1596 (7.14)	237 (0.65)
**Female; n (%)**	**18,666 (51.3)**	93 (55.36)	284 (24.03)	4,640 (52.74)	3,058 (45.47)	8,008 (53.83)	961 (54.08)	1,453 (55.97)	169 (71.31)
**Age; mean (SD), years**	**55.1 (19.3)**	77.57 (13.94)	60.31 (17.29)	61.56 (21.11)	54.75 (20.69)	52.83 (17.24)	49.10 (17.59)	48.91 (15.97)	43.55 (15.66)
**Age group; n (%)**									
18–34 years	**6,848 (18.8)**	3 (1.79)	125 (10.58)	1320 (15)	1489 (22.14)	2798 (18.81)	474 (26.67)	561 (21.61)	78 (32.91)
35–64 years	**16,788 (46.2)**	22 (13.1)	508 (42.98)	3069 (34.88)	2767 (41.14)	7816 (52.54)	895 (50.37)	1567 (60.36)	144 (60.76)
≥65 years	**12,724 (35.0)**	143 (85.12)	549 (46.45)	4409 (50.11)	2469 (36.71)	4263 (28.65)	408 (22.96)	468 (18.03)	15 (6.33)
**Platelet Count; mean (SD), G/L**	**249.5 (83.2)**	233.26 (76.62)	219.87 (61.87)	244.04 (79.91)	240.11 (87.53)	255.11 (86.84)	264.84 (79.18)	264.48 (65.8)	241.19 (58.55)
**ASAT; mean (SD), IU/L**	**29.4 (34.4)**	22.46 (15.28)	27.02 (14.01)	30.07 (34.07)	41.94 (62.05)	24.62 (15.2)	27.62 (23.5)	25.24 (15.27)	22.84 (12.48)
**ALAT; mean (SD), IU/L**	**28.3 (28.9)**	18.51 (22.49)	28.14 (21.48)	24.97 (28.28)	36.03 (43.63)	26.29 (19.75)	31.67 (34.44)	30.01 (20.94)	26.73 (25.77)
**HbA1c; N or n (%)**	**12,229**	40 (0.33)	1,041 (8.51)	1,148 (9.39)	1,299 (10.62)	4,639 (37.93)	2,389 (18.72)	1,628 (13.31)	45 (0.37)
**HbA1c; mean (SD), mmol/mol**	**40.2 (12.2)**	43.7 (12.35)	40.82 (9.91)	47.18 (17.38)	42.68 (14.61)	38.65 (10.13)	38.74 (10.24)	39.44 (13.03)	42.33 (13.84)
**HbA1c; n (%)**									
<39 mmol/mol	**7,549 (61.73)**	19 (47.5)	562 (53.99)	420 (36.59)	678 (52.19)	3116 (67.17)	1581 (66.18)	1148 (70.52)	25 (55.56)
39–47 mmol/mol	**2,836 (23.19)**	12 (30)	330 (31.7)	363 (31.62)	359 (27.64)	996 (21.47)	505 (21.14)	261 (16.03)	10 (22.22)
≥48 mmol/mol	**1,844 (15.08)**	9 (22.5)	149 (14.31)	365 (31.79)	262 (20.17)	527 (11.36)	303 (12.68)	219 (13.45)	10 (22.22)
**Total cholesterol; N or n (%)**	**12,649**	19 (0.15)	999 (7.9)	825 (6.5)	1112 (8.79)	5067 (40.6)	2298 (18.17)	2304 (18.21)	25 (0.2)
**Total cholesterol; mean (SD), mmol/L**	**4.6 (1.2)**	4.08 (1.2)	4.25 (1.14)	4.48 (1.31)	4.35 (1.28)	4.85 (1.21)	4.68 (1.18)	4.52 (1.14)	5.29 (1.33)
**Total cholesterol; n (%)**									
<5.2 mmol/L	**8,778 (69.40)**	16 (84.21)	800 (80.08)	598 (72.48)	827 (74.37)	3,1207 (63.29)	1,1608 (69.97)	1,1708 (74.13)	14 (56)
5.2–6.19 mmol/L	**2,630 (20.79)**	1 (5.26)	141 (14.11)	151 (18.3)	195 (17.54)	1241 (24.49)	472 (20.54)	424 (18.4)	5 (20)
≥6.2 mmol/L	**1,241 (9.81)**	2 (10.53)	58 (5.81)	76 (9.21)	90 (8.09)	619 (12.22)	218 (9.49)	172 (7.47)	6 (24)

SD, standard deviation; ASAT, aspartate aminotransferase; ALAT, alanine aminotransferase; HbA1c, glycosylated hemoglobin.

^†^encompasses secondary hospital with heterogenous patient cohorts.

^‡^encompasses the following clinics: Ophthalmology, Infectiology, Hematology and Central hematological Laboratory, Pneumology & Allergology, Medical and radio oncology & Nuclear medicine, Anesthesiology and Pain management, Nephrology & Hypertension, Urology, Otorhinolaryngology, Osteoporosis, General internal medicine, Dermatology & Venereology, Rheumatology & Immunology, Neurology & Neurosurgery and Obstetrics and Gynaecology.

^§^encompasses the following clinics: Angiology & Vascular surgery, Visceral surgery and medicine, Orthopaedic surgery and Thoracic surgery.

For all clinics the mean values of HbA1c were increased and belonged to ranges indicating prediabetes, except for Medicine and Surgery. Secondary hospital showed the highest proportion of patients with HbA1c levels ≥48 mmol/mol indicating diabetes. Over all clinics (except for Psychosomatic medicine) approximately two-thirds of the patients were showing healthy levels of total cholesterol. The cohort characteristics over all 26 clinics can be find in Table A in S1 Appendix.

**Fig 1:**
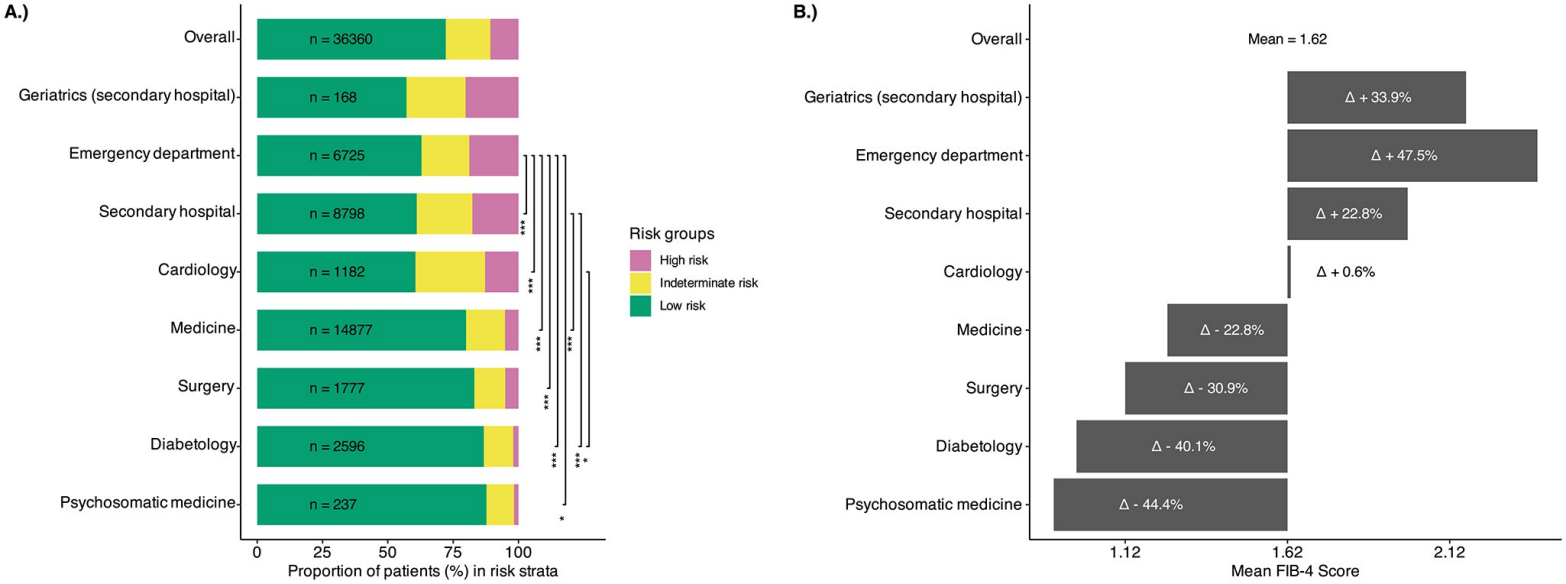
Comparisons between outpatient clinics. Graphical illustration A.) of proportions of patients with low, indeterminate and high risk of advanced fibrosis based on the lower FIB-4 score cutoff of <1.3 (<65 years) and <2.0 (≥65 years) and upper FIB-4 score cutoff of >2.67 and B.) of means of FIB-4 score. FIB-4, Fibrosis-4. ***P<0.001, *P<0.05.

The average value of FIB-4 score ranged from 0.9 (Psychosomatic medicine) to 2.39 (Emergency department) over the clinics, where the latter showed significant higher FIB-4 score values compared to the majority of other clinics (Fig 1).

Among the 36,360 patients, 26,245 (72.2%) had a low risk of advanced fibrosis, whereas 3,913 (10.8%) and 2597 (7.1%) had a high risk of advanced fibrosis defined by the two upper cutoff values of FIB-4 score >2.67 and FIB-4 score >3.25 respectively. The number of patients with an indeterminate risk of advanced fibrosis were 6,202 (17.06%) and 7,518 (20.7%) classified by the lower cutoff value of FIB-4 score (<1.3 (or <2.0 for patients ≥65 years old)) and the two upper cutoff values of FIB-4 score >2.67 and FIB-4 score >3.25 respectively. Change in upper cutoff values of FIB-4 score from >2.67 to >3.25 is reclassifying approximately 4% of the patients (Table 2). By using an upper cutoff value of FIB-4 score of >2.67 the proportions of patient at risk for indeterminate and high risk of advanced fibrosis according to the clinic were as follow: Geriatrics (43%), Cardiology (39%), secondary hospitals (39%), Emergency department (37%), Medicine (20%), Surgery (17%), Diabetology (13%) and Psychosomatic medicine (12%).

**Table 2:** Risk stratification for advanced fibrosis by FIB-4 score.

	N or n (%)	FIB-4 score						Patients (%) affected by changing the upper cutoff value^#^
		Lower cutoff: <1.3 (<65 years); <2.0 (≥65 years) Upper cutoff: >2.67			Lower cutoff: <1.3 (<65 years); <2.0 (≥65 years) Upper cutoff: >3.25			
		Low risk of ≥ F3 fibrosis	Indeterminate risk of ≥ F3 fibrosis	High risk of ≥ F3 fibrosis	Low risk of ≥ F3 fibrosis	Indeterminate risk of ≥ F3 fibrosis	High risk of ≥ F3 fibrosis	
**Overall**	**36,360**	**26,245 (72.18)**	**6,202 (17.06)**	**3,913 (10.76)**	**26,245 (72.18)**	**7,518 (20.68)**	**2,597 (7.14)**	**3.6**
Geriatrics (secondary hospital)	**168 (0.46)**	96 (57.14)	38 (22.62)	34 (20.24)	96 (57.14)	49 (29.17)	23 (13.69)	6.5
Cardiology	**1182 (3.25)**	716 (60.58)	315 (26.65)	151 (12.77)	716 (60.58)	385 (32.57)	81 (6.85)	5.9
Secondary hospital^†^	**8798 (24.2)**	5373 (61.07)	1874 (21.3)	1551 (17.63)	5373 (61.07)	2375 (26.99)	1050 (11.93)	5.7
Emergency department	**6725 (18.5)**	4231 (62.91)	1229 (18.28)	1265 (18.81)	4231 (62.91)	1590 (23.64)	904 (13.44)	5.4
Medicine^‡^	**14877 (40.92)**	11892 (79.94)	2219 (14.92)	766 (5.15)	11892 (79.94)	2530 (17.01)	455 (3.06)	2.1
Surgery^§^	**1777 (4.89)**	1477 (83.12)	210 (11.82)	90 (5.06)	1477 (83.12)	242 (13.62)	58 (3.26)	1.8
Diabetology	**2596 (7.14)**	2252 (86.75)	292 (11.25)	52 (2)	2252 (86.75)	321 (12.37)	23 (0.89)	1.1
Psychosomatic medicine	**237 (0.65)**	208 (87.76)	25 (10.55)	4 (1.69)	208 (87.76)	26 (10.97)	3 (1.27)	0.4

FIB-4, Fibrosis-4 score; F3, advanced fibrosis.

^†^encompasses secondary hospital with heterogenous patient cohorts.

^‡^encompasses the following clinics: Ophthalmology, Infectiology, Hematology and Central hematological Laboratory, Pneumology & Allergology, Medical and radio oncology & Nuclear medicine, Anesthesiology and Pain management, Nephrology & Hypertension, Urology, Otorhinolaryngology, Osteoporosis, General internal medicine, Dermatology & Venereology, Rheumatology & Immunology, Neurology & Neurosurgery and Obstetrics and Gynaecology.

^§^encompasses the following clinics: Angiology & Vascular surgery, Visceral surgery and medicine, Orthopaedic surgery and Thoracic surgery.

^#^Increasing the upper cutoff from >2.67 to >3.25 is reclassifying patients (%) from "high" to "Indeterminate risk of ≥ F3 fibrosis".

The clinic comparisons but using an upper cutoff value of FIB-4 score of >3.25 is shown in Fig A in S1 Appendix.

Geriatrics (20% if FIB-4>2.67; 14% FIB-4>3.25), Emergency department (19% if FIB-4>2.67; 13% FIB-4>3.25) and secondary hospitals (18% if FIB-4>2.67; 12% FIB-4>3.25) had the highest proportion of patient at high risk for of advanced fibrosis, whereas Diabetology (2% if FIB-4>2.67; 1% FIB-4>3.25) and Psychosomatic medicine (2% if FIB-4>2.67; 1% FIB-4>3.25) when compared over all clinics (Table 2).

We observed an increasing prevalence of male sex in FIB-4 strata indicating increased risk of advanced liver fibrosis, whereas age is significantly increasing. The average value of HbA1c was higher over the risk strata of advanced liver fibrosis overall and in Diabetology, but the same trends are not found for the other clinics. No correlation between FIB-4 score vs. Hb1Ac levels could be shown (Fig B1 in S1 Appendix). The average value of total cholesterol decreased over the risk strata of advanced liver fibrosis overall and all clinics, except for Surgery. No correlation between FIB-4 score vs. total cholesterol levels could be shown (Fig B2 in S1 Appendix).

Tables B-F in S1 Appendix provide a detailed overview of cohort and clinical laboratory parameters, both for the 8 main clinic groups and for all 26 clinics individually. Table F in S1 Appendix shows the comparison of risk stratification for advanced fibrosis by FIB-4 score in hematological settings.

## Discussion

Identifying patients at high risk for advanced hepatic fibrosis in routine practice is a largely unmet need in hepatology. Outpatient clinics at tertiary referral hospital could be important players in this complex task, as a large proportion of individuals with risk factors for metabolic dysfunction associated liver disease have comorbidities linking them to tertiary care in departments other than hepatology. If cases of compensated advanced liver disease could be promptly suspected, internal referral to hepatology can take place directly in tertiary care centers, and patients could complete the needed work-up and be started on treatment for liver disease or provided access to clinical trials. Risk stratification and improvement in linkage to care requires the rational use of non-invasive fibrosis tests. Therefore, the automatic calculation and systematic reporting of the simple, available, and affordable FIB-4 score in populations at risk of hepatic fibrosis in tertiary care could be appropriate.

This study provided an estimate of patients at risk of advanced fibrosis in tertiary care practice focusing on outpatient clinics. By applying the current recommendation of 2-step care pathway [2, 23, 27, 28], a high proportion (28%) of patients were flagged to require a further in-depth hepatological investigation. This would result in 17% of the flagged patients requiring a second-line test such as enhanced liver fibrosis (ELF) and 11% of the flagged patients being directly referred to liver specialists for specialist’s assessment. The proportion of patients requiring further hepatological investigations in this study was similar to that of a recently published French study, where patients in primary care were screened using FIB-4 during routine check-up [29]. Other screening studies, all performed in patients attending primary care,—flagged in France lower (19%) [30] and in Spain higher (49%) [31] portions of patients with indeterminate or high risk of advance liver fibrosis compared to this study.

Due to the known association between FIB-4 score values and cardiovascular diseases [18, 32, 33], it was not unexpected that at Cardiology a high proportion of patients requiring further in-depth hepatological investigation was flagged. As for the high proportion of patients with FIB-4 score values indicating an indeterminate and high risk for advanced liver fibrosis at the Emergency department, it could be the result of hospital access of patients with liver disease [34, 35] but could also represent the prevalence of unknown liver fibrosis inpatients with risk factors such as Type 2 diabetes and metabolic syndrome, who face liver-unrelated clinical events [36, 37]. However also acute non-liver related diseases (e.g. muscle diseases, viral infections, severe hemolytic anemia) [38–40] or medications may lead to misleadingly high FIB-4 score emergency department.

As advanced liver fibrosis is common in patients with Type 2 diabetes mellitus (T2DM), we expected to observe a high FIB-4 score in patients attending the Diabetology clinic [41]. Surprisingly we observed the contrary and we hypothesize that this depends on optimal care and younger age as compared to other clinics. Additionally, the accuracy of FIB-4 in T2DM is disputed [15–18, 42, 43], and lower scores do not always imply lack of fibrosis. However, a regular monitoring of the liver health and an accurate evaluation of liver fibrosis in this patient population remains important especially as the degree of liver fibrosis and T2DM-related complications show a strong correlation [44]. Similar to another study [29], the proportion of patients at high risk of advances fibrosis was increasing with increasing glucose levels in our study.

Traditionally, high cholesterol levels have been associated with metabolic dysfunction-associated steatotic liver disease (MASLD) and considered a risk factor for liver fibrosis [45]. However, our study revealed a counterintuitive finding, since we observed a lower cholesterol level in groups with higher risk for advanced fibrosis.

Despite adjusting the lower cutoff value of FIB-4 scores for patients equal or older than 65 years old to account for acceptable specificity and age being of the FIB-4 score equation, age is included in the FIB-4 formula and remains an important confounder for advanced liver fibrosis [24] since FIB-4 scores increases with age [8]. This trend was evident in our study, with geriatric and secondary hospital cohorts harboring the oldest patients and demonstrating this association. Given that FIB-4 score increases with age, considering factors like platelet count <150 G/L and/or AST/ALT ratio >0.8 in patients over 80 years could support identify those at actual risk for liver fibrosis, rather than attributing elevated scores solely to age.

Furthermore, this study revealed a statistically significant association between sex and FIB-4 score distribution, where men were more prone to be classified into the group of high risk of advanced liver fibrosis compared to women. This aligns with existing literature suggesting men are more likely to develop and die from liver fibrosis than women [46, 47]. Notably, the understanding regarding sex-specific risk and FIB-4 scores in advanced liver fibrosis remains incomplete.

The large sample size, which was collected over one full year, is a strength of this study. However, the following limitations have to be discussed. First, a linkage between the patient’s FIB-4 scores and history is impossible due to the retrospective study design based on coded patients’ data. Second, no diagnostic performance of FIB-4 in our patient collective could be performed as information on patient’s liver disease is not available. Therefore the accuracy of the FIB-4 score in the outpatient setting of a large tertiary care center setting remains unclear. Third, several patient’s demographic (e.g. race, education, place of residence) and clinical data (e.g. body mass index, blood pressure), which correlate with risk factors for liver disease, is lacking. Fourth, currently no guidelines are recommending the use of FIB-4 score in an unselected population. Therefore, it would have been of major interest to address the unselected versus targeted testing approach to reduce avoidable follow-up hepatological investigations and identifying outpatient clinics at risk of false positive FIB-4 scores due to acute non-liver related diseases. As the used dataset does not include any information on risk factors related to high-risk populations (e.g. obesity, diabetes, increased alcohol intake) a study specifically addressing this issue will be required. Fifth, the FIB-4 score can be misleading in patients with hematological diseases. Low platelet counts (immune thrombocytopenia) might falsely raise the score, while high platelet counts (essential thrombocythemia) might falsely lower it. Sixth, the gender differences in transaminases (ASAT and ALAT) were not taking into account.

Nowadays two different upper FIB-4 score cutoffs (FIB-4 >3.25 [14, 23] or FIB-4 >2.67 [48]) are used in clinical practice to flag patients with an risk of advanced of liver fibrosis requiring a complete hepatological workup. However, medical societies and expert groups [7, 12, 48–50] are recommending a upper cutoff FIB-4>2.67 to rule in patients with advanced hepatic fibrosis.

In conclusion, this study demonstrated a high prevalence of subjects at high risk of carrying advanced liver fibrosis in the outpatient setting of a large tertiary care center based on the calculation of FIB-4. This non-invasive and simple to obtain score could be automatized and used as a screening tool to flag outpatients at high risk for advanced liver fibrosis in a tertiary care setting. If this strategy is considered, a careful analysis of the existing and needed resources at the hepatology departments of tertiary care hospital should be carried out to optimize internal pathways of care.

## Supporting information

S1 Appendix Supporting information. (DOCX)

## Abbreviations

ALAT: alanine aminotransferase
ANOVA: analysis of variance
ASAT: aspartate aminotransferase
CLD: chronic liver disease
ELF: enhanced liver fibrosis
F3: advanced fibrosis
FIB-4: Fibrosis-4 score
HbA1c: glycated hemoglobin
HRA: Human Research Act
HRO: Human Research Ordinance
MASLD: metabolic dysfunction-associated steatotic liver disease
SD: standard deviation
T2DM: Type 2 diabetes mellitus
