# Supplementary material for: Estimating the prevalence of adults at risk for advanced hepatic fibrosis using FIB-4 in a Swiss tertiary care hospital
Source: PLoS One. 2025 Jan 24;20(1):e0317629. doi: 10.1371/journal.pone.0317629 (PMC11759403; doi:10.1371/journal.pone.0317629)
Supplement: S1 Appendix — (DOCX) [file pone.0317629.s001.docx]

**Supporting Information**

**Contents**

[1. **Table A.** Cohort characteristics over all 26 clinics. 2](#_Toc184597120)

[2**. Figure A.** Clinic comparisons. 5](#_Toc184597121)

[3**. Figure B.** Scatter plot of **1)** FIB-4 score vs. glycosylated hemoglobin (HbA1c) and **2)** FIB-4 score vs. total cholesterol at different cut-off values for all patients. 6](#_Toc184597122)

[4. **Table B.** Cohort and clinical laboratory parameters over all 8 main clinic groups by F3 fibrosis risk strata, using an upper FIB-4 cut-off value of 2.67. 7](#_Toc184597123)

[5. **Table C.** Cohort and clinical laboratory parameters over all 26 clinics individually by F3 fibrosis risk strata, using an upper FIB-4 cut-off value of 2.67. 10](#_Toc184597124)

[6. **Table D.** Cohort and clinical laboratory parameters over all 8 main clinic groups by F3 fibrosis risk strata, using an upper FIB-4 cut-off value of 3.25. 17](#_Toc184597125)

[7. **Table E.** Cohort and clinical laboratory parameters over all 26 clinics individually by F3 fibrosis risk strata, using an upper FIB-4 cut-off value of 3.25. 20](#_Toc184597126)

[8. **Table F.** Comparison of risk stratification for advanced fibrosis by FIB-4 score in hematological settings. 27](#_Toc184597127)

[9. **Text A.** The structure of the Swiss health care system is explained in Text A in S1 Appendix. 28](#_Toc184597128)

# 1. **Table A.** Cohort characteristics over all 26 clinics.

|  | **Overall** | **Angiology & Vascular surgery** | **Cardiology** | **Neurology & Neurosurgery** | **Psychosomatic medicine** | **Ophthalmology** | **Obstetrics and Gynaecology** | **Anesthesiology and  Pain management** | **Infectiology** |
| --- | --- | --- | --- | --- | --- | --- | --- | --- | --- |
| **N or n (%)** | **36,360** | 31 (0.09) | 1,182 (3.25) | 1,800 (4.95) | 237 (0.65) | 62 (0.17) | 1,629 (4.48) | 24 (0.07) | 990 (2.72) |
| **Female; n (%)** | **18,666 (51.3)** | 15 (48.39) | 284 (24.03) | 1,121 (62.28) | 169 (71.31) | 27 (43.55) | 1,589 (97.54) | 13 (54.17) | 334 (33.74) |
| **Age; mean (SD), years** | **55.1 (19.3)** | 49.71 (19.31) | 60.31 (17.29) | 46.9 (16.12) | 43.55 (15.66) | 62.89 (19.27) | 43.42 (15.37) | 58.17 (18.03) | 50.81 (13.06) |
| **Age group; n (%)** |  |  |  |  |  |  |  |  |  |
| 18-34 years | **6,848 (18.8)** | 7 (22.58) | 125 (10.58) | 494 (27.44) | 78 (32.91) | 8 (12.9) | 576 (35.36) | 3 (12.5) | 115 (11.62) |
| 35-64 years | **16,788 (46.2)** | 16 (51.61) | 508 (42.98) | 1032 (57.33) | 144 (60.76) | 21 (33.87) | 879 (53.96) | 13 (54.17) | 742 (74.95) |
| ≥65 years | **12,724 (35.0)** | 8 (25.81) | 549 (46.45) | 274 (15.22) | 15 (6.33) | 33 (53.23) | 174 (10.68) | 8 (33.33) | 133 (13.43) |
| **Platelet Count; mean (SD), G/L** | **249.5 (83.2)** | 224.94 (60.78) | 219.87 (61.87) | 259.78 (61.09) | 241.19 (58.55) | 238.9 (64.53) | 253.17 (66.6) | 255.38 (100.99) | 233.52 (62.74) |
| **ASAT; mean (SD), IU/L** | **29.4 (34.4)** | 24.84 (10.76) | 27.02 (14.01) | 23.54 (13.5) | 22.84 (12.48) | 27.66 (17.51) | 23.41 (20.16) | 26.75 (15.98) | 26.95 (17.65) |
| **ALAT; mean (SD), IU/L** | **28.3 (28.9)** | 26.52 (21.82) | 28.14 (21.48) | 25.81 (17.21) | 26.73 (25.77) | 28.63 (22.75) | 22.18 (17.7) | 29.33 (21.83) | 28.66 (18.95) |
| **HbA1c; N or n (%)** | **12,229** | 19 (0.16) | 1041 (8.51) | 561 (4.59) | 45 (0.37) | 17 (0.14) | 481 (3.93) | 6 (0.05) | 224 (1.83) |
| **HbA1c; mean (SD), mmol/mol** | **40.2 (12.2)** | 36.77 (6.03) | 40.82 (9.91) | 36.79 (7.77) | 42.33 (13.84) | 44.12 (15.26) | 34.54 (7.03) | 43.7 (14.3) | 40.11 (11.96) |
| **HbA1c range; n (%)** |  |  |  |  |  |  |  |  |  |
| <39 mmol/mol (normal) | **7,549 (61.73)** | 11 (57.89) | 562 (53.99) | 420 (74.87) | 25 (55.56) | 8 (47.06) | 424 (88.15) | 4 (66.67) | 138 (61.61) |
| 39-47 mmol/mol (prediabetes) | **2,836 (23.19)** | 8 (42.11) | 330 (31.7) | 110 (19.61) | 10 (22.22) | 5 (29.41) | 40 (8.32) | 1 (16.67) | 58 (25.89) |
| ≥48 mmol/mol (diabetes) | **1,844 (15.08)** | na | 149 (14.31) | 31 (5.53) | 10 (22.22) | 4 (23.53) | 17 (3.53) | 1 (16.67) | 28 (12.5) |
| **Total cholesterol; N or n (%)** | **12,649** | 19 (0.15) | 999 (7.9) | 155 (1.23) | 25 (0.2) | 12 (0.09) | 741 (5.86) | 6 (0.05) | 797 (6.3) |
| **Total cholesterol; mean (SD), mmol/L** | **4.6 (1.2)** | 4.79 (1.36) | 4.25 (1.14) | 4.85 (1.13) | 5.29 (1.33) | 4.83 (1.06) | 5.11 (1) | 4.63 (0.81) | 5 (1.13) |
| **Total cholesterol range; n (%)** |  |  |  |  |  |  |  |  |  |
| <5.2 mmol/L (healthy level) | **8,778 (69.40)** | 11 (57.89) | 800 (80.08) | 99 (63.87) | 14 (56) | 8 (66.67) | 409 (55.2) | 5 (83.33) | 467 (58.59) |
| 5.2-6.19 mmol/L (borderline high level) | **2,630 (20.79)** | 5 (26.32) | 141 (14.11) | 37 (23.87) | 5 (20) | 3 (25) | 237 (31.98) | 1 (16.67) | 218 (27.35) |
| ≥6.2 mmol/L (high level) | **1,241 (9.81)** | 3 (15.79) | 58 (5.81) | 19 (12.26) | 6 (24) | 1 (8.33) | 95 (12.82) | na | 112 (14.05) |

**Table A.** (Continued.)

|  | **Emergency department** | **Medical and radio oncology  & Nuclear medicine** | **Otorhinolaryngology** | **Orthopaedic surgery** | **Osteoporosis** | **General internal medicine** | **Hematology and Central  hematological Laboratory** | **Rheumatology & Immunology** | **Pneumology & Allergology** |
| --- | --- | --- | --- | --- | --- | --- | --- | --- | --- |
| **N or n (%)** | 6,725 (18.5) | 2,668 (7.34) | 20 (0.06) | 74 (0.2) | 6 (0.02) | 762 (2.1) | 1,139 (3.13) | 2,313 (6.36) | 560 (1.54) |
| **Female; n (%)** | 3,058 (45.47) | 922 (34.56) | 10 (50) | 35 (47.3) | 2 (33.33) | 407 (53.41) | 552 (48.46) | 1,495 (64.63) | 258 (46.07) |
| **Age; mean (SD), years** | 54.75 (20.69) | 61.43 (15.32) | 59.25 (15.24) | 59.93 (18.66) | 61 (14.89) | 49.84 (16.36) | 55.34 (18.75) | 52.28 (16.58) | 56.77 (16.71) |
| **Age group; n (%)** |  |  |  |  |  |  |  |  |  |
| 18-34 years | 1489 (22.14) | 204 (7.65) | 2 (10) | 9 (12.16) | 0 (0) | 165 (21.65) | 214 (18.79) | 412 (17.81) | 70 (12.5) |
| 35-64 years | 2767 (41.14) | 1147 (42.99) | 9 (45) | 29 (39.19) | 3 (50) | 447 (58.66) | 486 (42.67) | 1304 (56.38) | 274 (48.93) |
| ≥65 years | 2469 (36.71) | 1317 (49.36) | 9 (45) | 36 (48.65) | 3 (50) | 150 (19.69) | 439 (38.54) | 597 (25.81) | 216 (38.57) |
| **Platelet Count; mean (SD), G/L** | 240.11 (87.53) | 260.72 (104.59) | 260.55 (60.76) | 279.49 (77.89) | 305.5 (153.68) | 251.65 (69.9) | 269.75 (161.02) | 260.86 (70.7) | 247.87 (82.9) |
| **ASAT; mean (SD), IU/L** | 41.94 (62.05) | 25.69 (15.04) | 22.8 (6.66) | 25.18 (15.37) | 33.67 (21.35) | 25.9 (19.04) | 25.52 (12.47) | 24.14 (16.46) | 24.95 (10.72) |
| **ALAT; mean (SD), IU/L** | 36.03 (43.63) | 27.07 (20.49) | 22.65 (8.77) | 26.26 (18.96) | 36.83 (19.67) | 29.83 (28.04) | 26.48 (18.73) | 26.58 (20.02) | 26.02 (16.2) |
| **HbA1c; N or n (%)** | 1299 (10.62) | 519 (4.24) | 3 (0.02) | 8 (0.07) | 5 (0.04) | 450 (3.68) | 240 (1.96) | 351 (2.87) | 139 (1.14) |
| **HbA1c; mean (SD), mmol/mol** | 42.68 (14.61) | 39.81 (11.14) | 34.78 (8.84) | 37.83 (5.86) | 45.12 (21.52) | 39.03 (8.77) | 37.29 (9.42) | 39.01 (9.34) | 40.81 (9.32) |
| **HbA1c range; n (%)** |  |  |  |  |  |  |  |  |  |
| <39 mmol/mol (normal) | 678 (52.19) | 317 (61.08) | 2 (66.67) | 4 (50) | 4 (80) | 294 (65.33) | 174 (72.5) | 234 (66.67) | 72 (51.8) |
| 39-47 mmol/mol (prediabetes) | 359 (27.64) | 131 (25.24) | 1 (33.33) | 4 (50) | na | 108 (24) | 43 (17.92) | 73 (20.8) | 41 (29.5) |
| ≥48 mmol/mol (diabetes) | 262 (20.17) | 71 (13.68) | na | na | 1 (20) | 48 (10.67) | 23 (9.58) | 44 (12.54) | 26 (18.71) |
| **Total cholesterol; N or n (%)** | 1112 (8.79) | 574 (4.54) | 1 (0.01) | 8 (0.06) | 4 (0.03) | 434 (3.43) | 248 (1.96) | 305 (2.41) | 135 (1.07) |
| **Total cholesterol; mean (SD), mmol/L** | 4.35 (1.28) | 4.72 (1.29) | 8.72 | 4.79 (0.94) | 4.99 (1.04) | 5.09 (1.26) | 4.43 (1.17) | 5.15 (1.29) | 4.46 (1.16) |
| **Total cholesterol range; n (%)** |  |  |  |  |  |  |  |  |  |
| <5.2 mmol/L (healthy level) | 827 (74.37) | 385 (67.07) | na | 6 (75) | 2 (50) | 239 (55.07) | 188 (75.81) | 169 (55.41) | 101 (74.81) |
| 5.2-6.19 mmol/L (borderline high level) | 195 (17.54) | 136 (23.69) | na | 1 (12.5) | 2 (50) | 129 (29.72) | 41 (16.53) | 66 (21.64) | 23 (17.04) |
| ≥6.2 mmol/L (high level) | 90 (8.09) | 53 (9.23) | 1 (100) | 1 (12.5) | na | 66 (15.21) | 19 (7.66) | 70 (22.95) | 11 (8.15) |

**Table A.** (Continued.)

|  | **Thoracic surgery** | **Dermatology & Venereology** | **Diabetology, Endocrinology,  Nutritional medicine &**  **Metabolism** | **Visceral surgery and medicine** | **Urology** | **Nephrology & Hypertension** | **Secondary hospital I†** | **Secondary hospital II‡** | **Geriatrics (secondary**  **hospital)§** |
| --- | --- | --- | --- | --- | --- | --- | --- | --- | --- |
| **N or n (%)** | 343 (0.94) | 1,250 (3.44) | 2,596 (7.14) | 1,329 (3.66) | 39 (0.11) | 1,615 (4.44) | 5152 (14.18) | 3,646 (10.03) | 168 (0.46) |
| **Female; n (%)** | 144 (41.98) | 598 (47.84) | 1,453 (55.97) | 767 (57.71) | 10 (25.64) | 670 (41.49) | 2702 (52.45) | 1,938 (53.15) | 93 (55.35) |
| **Age; mean (SD), years** | 49.56 (18.73) | 50.84 (18.52) | 48.91 (15.97) | 48.37 (17) | 65.49 (14.49) | 55.69 (16.59) | 61.92 (20.83) | 61.05 (21.51) | 77.56 (13.94) |
| **Age group; n (%)** |  |  |  |  |  |  |  |  |  |
| 18-34 years | 96 (27.99) | 305 (24.4) | 561 (21.61) | 362 (27.24) | 1 (2.56) | 229 (14.18) | 730 (14.17) | 590 (16.18) | 3 (1.79) |
| 35-64 years | 150 (43.73) | 614 (49.12) | 1567 (60.36) | 700 (52.67) | 15 (38.46) | 830 (51.39) | 1809 (35.11) | 1260 (34.56) | 22 (13.1) |
| ≥65 years | 97 (28.28) | 331 (26.48) | 468 (18.03) | 267 (20.09) | 23 (58.97) | 556 (34.43) | 2613 (50.72) | 1796 (49.26) | 143 (85.12) |
| **Platelet Count; mean (SD), G/L** | 271.91 (84.83) | 261.58 (68.74) | 264.48 (65.8) | 263.13 (77.77) | 268.03 (116.06) | 236.48 (69.75) | 241.26 (76.03) | 247.98 (84.95) | 233.26 (76.63) |
| **ASAT; mean (SD), IU/L** | 23.91 (8.97) | 24.87 (11.79) | 25.24 (15.27) | 28.77 (26.39) | 21.59 (6.79) | 22.9 (10.15) | 28.16 (30.85) | 32.78 (38.01) | 22.46 (15.28) |
| **ALAT; mean (SD), IU/L** | 26.59 (17.86) | 28.18 (22.17) | 30.01 (20.94) | 33.4 (38.24) | 23 (12.5) | 24.62 (17.46) | 24.26 (26.85) | 25.97 (30.17) | 18.51 (22.49) |
| **HbA1c; N or n (%)** | 33 (0.27) | 251 (2.05) | 1628 (13.31) | 2329 (19.04) | 9 (0.07) | 1383 (11.31) | 643 (5.25) | 505 (4.13) | 40 (0.33) |
| **HbA1c; mean (SD), mmol/mol** | 36.07 (6.97) | 40.71 (11.27) | 39.44 (13.03) | 38.79 (10.31) | 36.96 (5.1) | 39.49 (11.09) | 47.35 (17.2) | 46.96 (17.62) | 43.7 (12.34) |
| **HbA1c range; n (%)** |  |  |  |  |  |  |  |  |  |
| <39 mmol/mol (normal) | 23 (69.7) | 150 (59.76) | 1148 (70.52) | 1543 (66.25) | 5 (55.56) | 870 (62.91) | 227 (35.3) | 193 (38.22) | 19 (47.5) |
| 39-47 mmol/mol (prediabetes) | 8 (24.24) | 58 (23.11) | 261 (16.03) | 485 (20.82) | 4 (44.44) | 323 (23.36) | 205 (31.9) | 158 (31.29) | 12 (30) |
| ≥48 mmol/mol (diabetes) | 2 (6.06) | 43 (17.13) | 219 (13.45) | 301 (12.92) | na | 190 (13.74) | 211 (32.81) | 154 (30.5) | 9 (22.5) |
| **Total cholesterol; N or n (%)** | 35 (0.28) | 293 (2.32) | 2304 (18.21) | 2236 (17.68) | 11 (0.09) | 1351 (10.68) | 478 (3.78) | 347 (2.74) | 19 (0.15) |
| **Total cholesterol; mean (SD), mmol/L** | 4.57 (1.11) | 4.92 (1.09) | 4.52 (1.14) | 4.68 (1.18) | 4.63 (1.35) | 4.62 (1.26) | 4.52 (1.27) | 4.42 (1.36) | 4.08 (1.2) |
| **Total cholesterol range; n (%)** |  |  |  |  |  |  |  |  |  |
| <5.2 mmol/L (healthy level) | 27 (77.14) | 178 (60.75) | 1708 (74.13) | 1564 (69.95) | 7 (63.64) | 950 (70.32) | 349 (73.01) | 249 (71.76) | 16 (84.21)) |
| 5.2-6.19 mmol/L (borderline high level) | 4 (11.43) | 76 (25.94) | 424 (18.4) | 462 (20.66) | 2 (18.18) | 270 (19.99) | 85 (17.78) | 66 (19.02) | 1 (5.26) |
| ≥6.2 mmol/L (high level) | 4 (11.43) | 39 (13.31) | 172 (7.47) | 210 (9.39) | 2 (18.18) | 131 (9.7) | 44 (9.21) | 32 (9.22) | 2 (110.52) |

**SD, standard deviation; ASAT, aspartate aminotransferase; ALAT, alanine aminotransferase;** **HbA1c, glycosylated hemoglobin.**

^†^encompasses heterogenous patient cohorts from the hospitals Aarberg, Riggisberg and Münsingen.

‡encompasses heterogenous patient cohorts from the hospital Tiefenau.

§encompasses the nursing home Frienisberg and Seelandheim.

# 2**. Figure A.** Clinic comparisons.


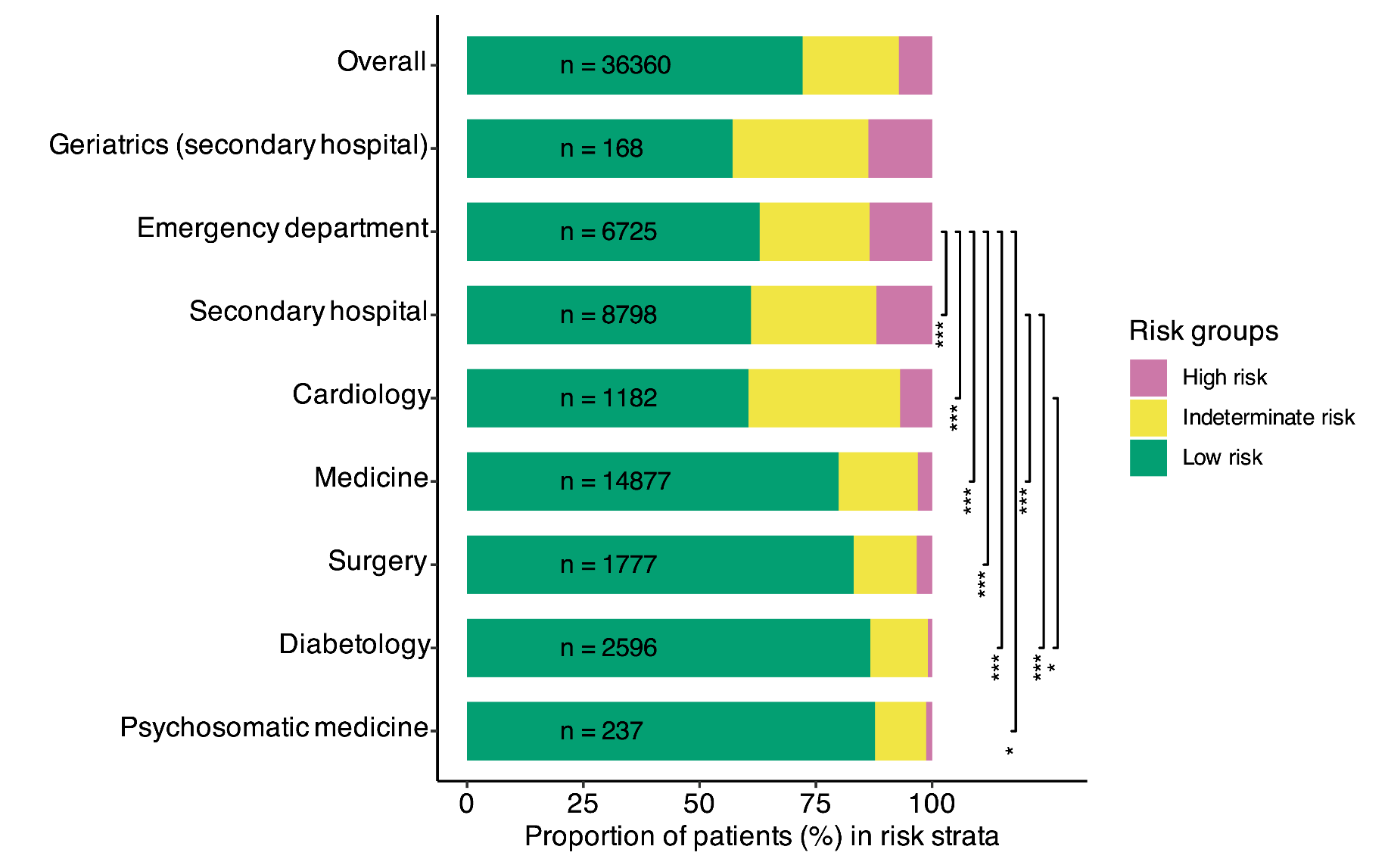


Graphical illustration A.) of proportions of patients with low, indeterminate and high risk of advanced fibrosis based on the lower FIB-4 score cutoff of <1.3 (<65 years) and <2.0 (≥65 years) and upper FIB-4 score cutoff of >3.25. FIB-4, Fibrosis-4. ***P<0.001, *P<0.05.

# 3**. Figure B.** Scatter plot of **1)** FIB-4 score vs. glycosylated hemoglobin (HbA1c) and **2)** FIB-4 score vs. total cholesterol at different cut-off values for all patients.

| **A** | **B** |
| --- | --- |
| 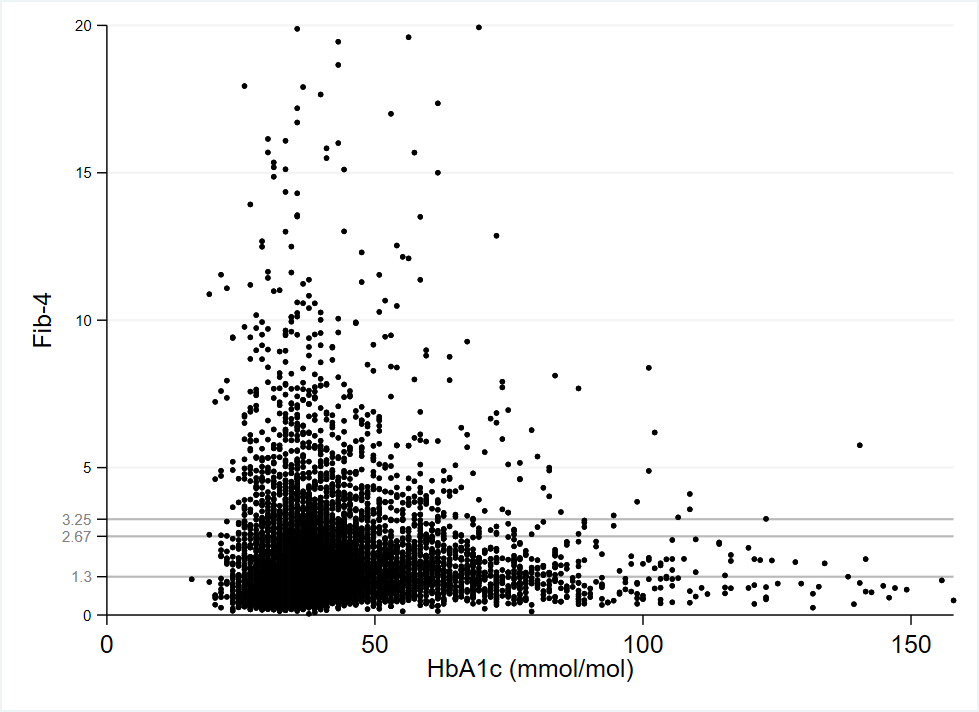 | 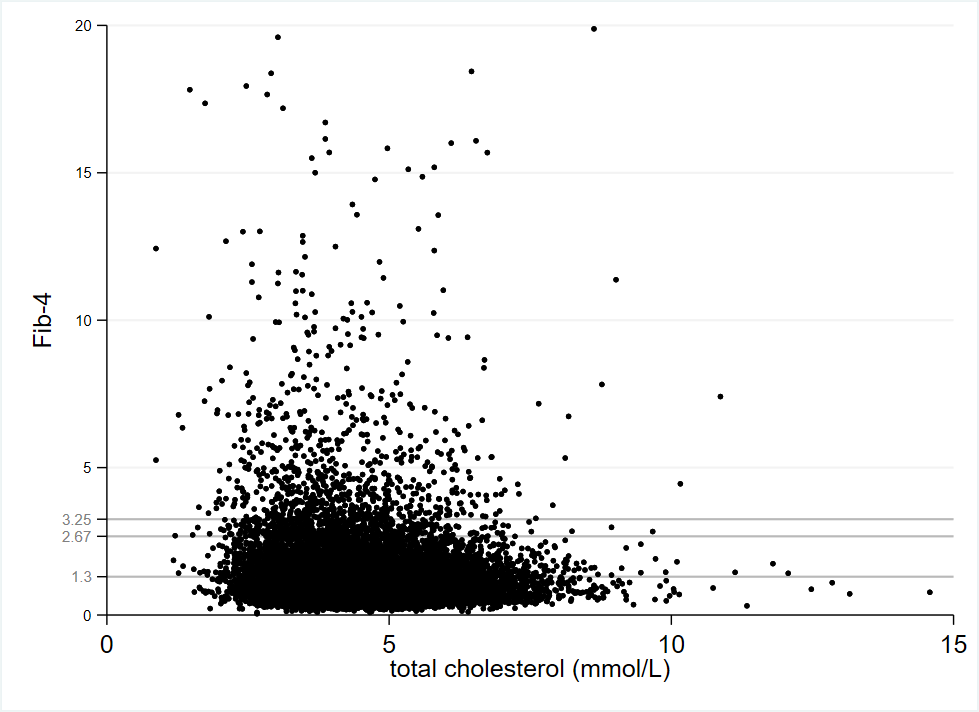 |

# 4. **Table B.** Cohort and clinical laboratory parameters over all 8 main clinic groups by F3 fibrosis risk strata, using an upper FIB-4 cut-off value of 2.67.

|  |  | **N or n (%)** | **Female;  n (%)** | | **Age; years** | | | **Age group;  n (%)** | | | | **HbA1c;  N or n (%)** | **HbA1c;  mmol/mol** | | |
| --- | --- | --- | --- | --- | --- | --- | --- | --- | --- | --- | --- | --- | --- | --- | --- |
|  |  |  |  | p-value | mean | SD | p-value | 18-34 years | 35-64 years | ≥65 years | p-value |  | mean | SD | p-value |
| **Overall** | **all** | **36,360** | **18,666 (51.34)** |  | **55.13** | **19.36** |  | **6,848 (18.8)** | **16,788 (46.2)** | **12,724 (35.0)** |  | **12,229** | **40.21** | **12.22** |  |
|  | low risk | 26,245 (72.18) | 14,301 (54.49) | <0.001ꬸ | 50.4 | 18.72 | <0.001† | 6,579 (25.07 ) | 12,380 (47.17) | 7,286 (27.76) | <0.001ꬸ | 8,464 (69.21) | 39.73 | 12.13 | <0.001† |
|  | indeterminate risk | 6,202 ( 17.06) | 2,760 (44.50) |  | 62.94 | 14.48 | <0.001◊ | 218 (3.51 ) | 3,666 (59.11) | 2,318 (37.38) |  | 2,323 (19) | 41.16 | 12.17 | <0.001◊ |
|  | high risk | 3,913 (10.76) | 1,605 (41.02) |  | 74.46 | 13.46 | <0.001¥ | 51 (1.3) | 742 (18.96) | 3,120 (79.73) |  | 1,442 (11.79) | 41.57 | 12.59 | ns¥ |
| **Geriatrics** | **all** | **168 (0.46)** | **93 (55.35)** |  | **77.57** | **13.93** |  | **3 (1.79)** | **22 (13.1)** | **143 (85.12)** |  | **40 (0.33)** | **43.7** | **12.34** |  |
| (secondary hospital) | low risk | 96 (57.14) | 54 (56.25) | nsꬸ | 74.25 | 14.64 | ns† | 3 (3.13) | 13 (13.54) | 80 (83.33) | <0.05ꬸ | 23 (57.5) | 43.3 | 11.74 | ns† |
|  | indeterminate risk | 38 (22.61) | 21 (55.26) |  | 78.45 | 13.38 | <0.001◊ | 0 (0) | 9 (23.68) | 29 (76.32) |  | 7 (17.5) | 49.56 | 13.82 | ns◊ |
|  | high risk | 34 (20.24) | 18 (52.94) |  | 85.94 | 7.77 | ns¥ | 0 (0) | 0 (0) | 34 (100) |  | 10 (25) | 40.53 | 12.56 | ns¥ |
| **Cardiology** | **all** | **1,182 (3.25)** | **284 (24.03)** |  | **60.31** | **17.29** |  | **125 (10.58)** | **508 (42.98)** | **549 (46.45)** |  | **1,041 (8.51)** | **40.82** | **9.91** |  |
|  | low risk | 716 (60.58) | 202 (28.21) | <0.001ꬸ | 55.21 | 17.77 | <0.001† | 115 (16.06) | 319 (44.55) | 282 (39.39) | <0.001ꬸ | 637 (61.19) | 40.61 | 9.58 | ns† |
|  | indeterminate risk | 315 (26.65) | 57 (18.1) |  | 63.97 | 12.9 | <0.001◊ | 10 (3.17) | 175 (55.56) | 130 (41.27) |  | 270 (25.94) | 41.25 | 11.44 | ns◊ |
|  | high risk | 151 (12.77) | 25 (16.56) |  | 76.91 | 8.44 | <0.001¥ | 0 (0) | 14 (9.27) | 137 (90.73) |  | 134 (12.87) | 40.99 | 8.01 | ns¥ |
| **Secondary hospital*** | **all** | **8,798 (24.2)** | **4,640 (52.74)** |  | **61.56** | **21.11** |  | **1,320 (15)** | **3,069 (34.88)** | **4,409 (50.11)** |  | **1,148 (9.39)** | **47.18** | **17.38** |  |
|  | low risk | 5,373 (61.07) | 2,894 (53.86) | <0.05ꬸ | 54.37 | 21.09 | <0.001† | 1,253 (23.32) | 2,054 (38.23) | 2,066 (38.45) | <0.001ꬸ | 584 (50.87) | 48.07 | 19.28 | ns† |
|  | indeterminate risk | 1874 (21.3) | 959 (51.17) |  | 67.21 | 16 | <0.001◊ | 60 (3.2) | 863 (46.05) | 951 (50.75) |  | 289 (25.17) | 45.18 | 14.5 | ns◊ |
|  | high risk | 1551 (17.63) | 787 (50.74) |  | 79.61 | 11.66 | <0.001¥ | 7 (0.45) | 152 (9.8) | 1,392 (89.75) |  | 275 (23.95) | 47.39 | 15.7 | ns¥ |
| **Emergency department** | **all** | **6,725 (18.5)** | **3,058 (45.47)** |  | **54.75** | **20.69** |  | **1,489 (22.14)** | **2,767 (41.14)** | **2,469 (36.71)** |  | **1,299 (10.62)** | **42.68** | **14.61** |  |
|  | low risk | 4,231 (62.91) | 2,096 (49.54) | <0.001ꬸ | 47.98 | 20.2 | <0.001† | 1,389 (32.83) | 1,715 (40.53) | 1,127 (26.64) | <0.001ꬸ | 699 (53.81) | 43.37 | 16.32 | ns† |
|  | indeterminate risk | 1,229 (18.28) | 495 (40.28) |  | 61.52 | 15.81 | <0.001◊ | 73 (5.94) | 708 (57.61) | 448 (36.45) |  | 284 (21.86) | 41.64 | 12.15 | ns◊ |
|  | high risk | 1,265 (18.81) | 467 (36.92) |  | 70.82 | 14.54 | <0.001¥ | 27 (2.13) | 344 (27.19) | 894 (70.67) |  | 316 (24.33) | 42.09 | 12.42 | ns¥ |
| **Medicine**** | **all** | **14,877 (40.92)** | **8,008 (53.83)** |  | **52.83** | **17.24** |  | **2,798 (18.81)** | **7,816 (52.54)** | **4,263 (28.65)** |  | **4,639 (37.93)** | **38.65** | **10.13** |  |
|  | low risk | 11,892 (79.94) | 6,731 (56.6) | <0.001ꬸ | 50.29 | 17.26 | <0.001† | 2,719 (22.86) | 6,108 (51.36) | 3,065 (25.77) | <0.001ꬸ | 3572 (77) | 38.46 | 10.09 | ns† |
|  | indeterminate risk | 2,219 (14.92) | 1,005 (45.29) |  | 60.46 | 12.25 | <0.001◊ | 66 (2.97) | 1,520 (68.5) | 633 (28.53) |  | 795 (17.14) | 39.48 | 10.03 | ns◊ |
|  | high risk | 766 (5.15) | 272 (35.51) |  | 70.12 | 12.19 | <0.001¥ | 13 (1.7) | 188 (24.54) | 565 (73.76) |  | 272 (5.86) | 38.71 | 10.86 | ns¥ |
| **Surgery***** | **all** | **1,777 (4.89)** | **961 (54.08)** |  | **49.1** | **17.59** |  | **474 (26.67)** | **895 (50.37)** | **408 (22.96)** |  | **2,389 (18.72)** | **38.74** | **10.24** |  |
|  | low risk | 1,477 (83.12) | 848 (57.41) | <0.001ꬸ | 46.17 | 17.09 | <0.001† | 469 (31.75) | 727 (49.22) | 281 (19.03) | <0.001ꬸ | 1476 (61.78) | 38.05 | 9.24 | <0.001† |
|  | indeterminate risk | 210 (11.82) | 92 (43.81) |  | 60.81 | 11.83 | <0.001◊ | 4 (1.9) | 142 (67.62) | 64 (30.48) |  | 507 (21.2) | 40.47 | 12.29 | ns◊ |
|  | high risk | 90 (5.06) | 21 (23.33) |  | 70.06 | 9.84 | <0.001¥ | 1 (1.11) | 26 (28.89) | 63 (70) |  | 406 (17) | 39.06 | 10.63 | ns¥ |
| **Diabetology** | **all** | **2,596 (7.14)** | **1,453 (55.97)** |  | **48.91** | **15.97** |  | **561 (21.61)** | **1,567 (60.36)** | **468 (18.03)** |  | **1,628 (13.31)** | **39.44** | **13.03** |  |
|  | low risk | 2,252 (86.75) | 1,325 (58.84) | <0.001ꬸ | 47.1 | 15.86 | <0.001† | 555 (24.64) | 1,325 (58.84) | 372 (16.52) | <0.001ꬸ | 1,435 (88.14) | 38.89 | 12.45 | <0.05† |
|  | indeterminate risk | 292 (11.25) | 116 (39.73) |  | 59.48 | 9.27 | <0.001◊ | 3 (1.03) | 227 (77.74) | 62 (21.23) |  | 165 (10.14) | 43.14 | 15.63 | <0.001◊ |
|  | high risk | 52 (2) | 12 (23.08) |  | 67.9 | 15.3 | <0.05¥ | 3 (5.77) | 15 (28.85) | 34 (65.38) |  | 28 (1.72) | 46.01 | 19.16 | ns¥ |
| **Psychosomatic medicine** | **all** | **237 (0.65)** | **169 (71.31)** |  | **43.55** | **15.66** |  | **78 (32.91)** | **144 (60.76)** | **15 (6.33)** |  | **45 (0.37)** | **42.33** | **13.84** |  |
|  | low risk | 208 (87.76) | 151 (72.6) | nsꬸ | 41.9 | 15.52 | <0.001† | 76 (36.54) | 119 (57.21) | 13 (6.25) | <0.05ꬸ | 38 (84.44) | 43.3 | 14.78 | ns† |
|  | indeterminate risk | 25 (10.55) | 15 (60) |  | 55.4 | 11.09 | ns◊ | 2 (8) | 22 (88) | 1 (4) |  | 6 (13.33) | 36.23 | 4.19 | ns◊ |
|  | high risk | 4 (1.69) | 3 (75) |  | 55.25 | 12.58 | ns¥ | 0 (0) | 3 (75) | 1 (25) |  | 1 (2.22) | 42.06 | na | ns¥ |

**Table B.** (Continued.)

|  |  | **HbA1c range;  n (%)** | | | | **Total cholesterol;  N or n (%)** | **Total cholesterol;  mmol/L** | | | **Total cholesterol range;  n (%)** | | | |
| --- | --- | --- | --- | --- | --- | --- | --- | --- | --- | --- | --- | --- | --- |
|  |  | <39 mmol/mol  (normal) | 39-47 mmol/mol  (prediabetes) | ≥48 mmol/mol  (diabetes) | p-value |  | mean | SD | p-value | <5.2 mmol/L  (healthy level) | 5.2-6.19 mmol/L  (borderline high level) | ≥6.2 mmol/L  (high level) | p-value |
| **Overall** | **all** | **7,549 (61.73)** | **2,836 (23.19)** | **1,844 (15.08)** |  | **12,649** | **4.64** | **1.22** |  | **8,778 (69.40)** | **2,630 (20.79)** | **1,241 (9.81)** |  |
|  | low risk | 5,482 (72.62) | 1,826 (64.39) | 1,156 (62.69) | <0.001ꬸ | 9,051 (71.56) | 4.73 | 1.2 | <0.001† | 6,115 (69.66) | 1,990 (75.67 ) | 946 (76.23) | <0.001ꬸ |
|  | indeterminate risk | 1,324 (17.54) | 605 (21.33) | 394 (21.37) |  | 2,377 (18.79) | 4.57 | 1.23 | <0.001◊ | 1,679 (19.13) | 477 ( 18.14) | 221 (17.81) |  |
|  | high risk | 743 (9.84) | 405 (14.28) | 294 (15.94) |  | 1,221 (9.65) | 4.16 | 1.26 | <0.001¥ | 984 (11.21) | 163 (6.20) | 74 (5.96) |  |
| **Geriatrics** | **all** | **19 (47.5)** | **12 (30)** | **9 (22.5)** |  | **19 (0.15)** | **4.08** | **1.2** |  | **16 (84.21))** | **1 (5.26)** | **2 (110.52)** |  |
| (secondary hospital) | low risk | 12 (63.16) | 6 (50) | 5 (55.56) | nsꬸ | 10 (52.63) | 4.65 | 1.36 | ns† | 7 (43.75) | 1 (100.00) | 2 (100.00) | nsꬸ |
|  | indeterminate risk | 1 (5.26) | 3 (25) | 3 (33.33) |  | 5 (26.32) | 3.63 | 0.65 | ns◊ | 5 (31.25) | 0 (0) | 0 (0) |  |
|  | high risk | 6 (31.58) | 3 (25) | 1 (11.11) |  | 4 (21.05) | 3.23 | 0.43 | ns¥ | 4 (25) | 0 (0) | 0 (0) |  |
| **Cardiology** | **all** | **562 (53.99)** | **330 (31.7)** | **149 (14.31)** |  | **999 (7.9)** | **4.25** | **1.14** |  | **800 (80.08)** | **141 (14.11)** | **58 (5.81)** |  |
|  | low risk | 347 (61.74) | 201 (60.91) | 89 (59.73) | nsꬸ | 601 (60.16) | 4.36 | 1.18 | ns† | 465 (58.13) | 89 (63.12) | 47 (81.03) | <0.05ꬸ |
|  | indeterminate risk | 153 (27.22) | 76 (23.03) | 41 (27.52) |  | 269 (26.93) | 4.19 | 1.04 | <0.001◊ | 220 (27.5) | 41 (29.08) | 8 (13.79) |  |
|  | high risk | 62 (11.03) | 53 (16.06) | 19 (12.75) |  | 129 (12.91) | 3.86 | 1 | <0.05¥ | 115 (14.37) | 11 (7.8) | 3 (5.17) |  |
| **Secondary hospital**^α^ | **all** | **420 (36.59)** | **363 (31.62)** | **365 (31.79)** |  | **825 (6.5)** | **4.48** | **1.31** |  | **598 (72.48)** | **151 (18.3)** | **76 (9.21)** |  |
|  | low risk | 223 (53.10) | 168 (46.28) | 193 (52.88) | nsꬸ | 454 (55.03) | 4.58 | 1.26 | ns† | 315 (52.68) | 97 (64.24) | 42 (55.26) | nsꬸ |
|  | indeterminate risk | 106 (25.24) | 107 (29.48) | 76 (20.82) |  | 214 (25.94) | 4.39 | 1.25 | <0.001◊ | 160 (26.76) | 34 (22.52) | 20 (26.32) |  |
|  | high risk | 91 (21.67) | 88 (24.24) | 96 (26.30) |  | 157 (19.03) | 4.28 | 1.48 | ns¥ | 123 (20.57) | 20 (13.25) | 14 (18.42) |  |
| **Emergency department** | **all** | **678 (52.19)** | **359 (27.64)** | **262 (20.17)** |  | **1,112 (8.79)** | **4.35** | **1.28** |  | **827 (74.37)** | **195 (17.54)** | **90 (8.09)** |  |
|  | low risk | 376 (55.46) | 173 (48.19) | 150 (57.25) | nsꬸ | 601 (54.05) | 4.57 | 1.28 | <0.05† | 417 (50.42) | 116 (59.49) | 68 (75.56) | <0.001ꬸ |
|  | indeterminate risk | 146 (21.53) | 89 (24.79) | 49 (18.7) |  | 245 (22.03) | 4.3 | 1.2 | <0.001◊ | 186 (22.49) | 45 (23.08) | 14 (15.56) |  |
|  | high risk | 156 (23.01) | 97 (27.02) | 63 (24.05) |  | 266 (23.92) | 3.92 | 1.25 | <0.05¥ | 224 (27.09) | 34 (17.44) | 8 (8.89) |  |
| **Medicine**^‡^ | **all** | **3,116 (67.17)** | **996 (21.47)** | **527 (11.36)** |  | **5,067 (40.6)** | **4.85** | **1.21** |  | **3,207 (63.29)** | **1,241 (24.49)** | **619 (12.22)** |  |
|  | low risk | 2,436 (78.18) | 745 (74.80) | 391 (74.19) | nsꬸ | 3,893 (76.83) | 4.9 | 1.19 | <0.05† | 2,417 (75.37) | 988 (79.61) | 488 (78.84) | <0.05ꬸ |
|  | indeterminate risk | 503 (16.14) | 189 (18.98) | 103 (19.54) |  | 901 (17.78) | 4.75 | 1.25 | <0.001◊ | 586 (18.27) | 207 (16.68) | 108 (17.45) |  |
|  | high risk | 177 (5.68) | 62 (6.22) | 33 (6.26) |  | 273 (5.39) | 4.37 | 1.23 | <0.001¥ | 204 (6.36) | 46 (3.71) | 23 (3.72) |  |
| **Surgery^§^** | **all** | **1,581 (66.18)** | **505 (21.14)** | **303 (12.68)** |  | **2,298 (18.17)** | **4.68** | **1.18** |  | **1,608 (69.97)** | **472 (20.54)** | **218 (9.49)** |  |
|  | low risk | 1,035 (65.46) | 295 (58.42) | 146 (48.18) | <0.001ꬸ | 1455 (63.4) | 4.75 | 1.12 | ns† | 999 (62.13) | 313 (66.31) | 143 (65.6) | <0.001ꬸ |
|  | indeterminate risk | 311 (19.67) | 113 (22.38) | 83 (27.39) |  | 492 (21.41) | 4.78 | 1.22 | <0.001◊ | 328 (20.4) | 110 (23.31) | 54 (24.77) |  |
|  | high risk | 235 (14.86) | 97 (19.21) | 74 (24.42) |  | 351 (15.27) | 4.23 | 1.24 | <0.001¥ | 281 (17.48) | 49 (10.38) | 21 (9.63) |  |
| **Diabetology** | **all** | **1,148 (70.52)** | **261 (16.03)** | **219 (13.45)** |  | **2,304 (18.21)** | **4.52** | **1.14** |  | **1,708 (74.13)** | **424 (18.4)** | **172 (7.47)** |  |
|  | low risk | 1,032 (89.9) | 231 (88.51) | 172 (78.54) | <0.001ꬸ | 2,016 (87.5) | 4.55 | 1.13 | <0.001† | 1,484 (86.89) | 382 (90.09) | 150 (87.21) | nsꬸ |
|  | indeterminate risk | 100 (8.71) | 26 (9.96) | 39 (17.81) |  | 247 (10.72) | 4.29 | 1.16 | ns◊ | 191 (11.18) | 39 (9.20) | 17 (9.88) |  |
|  | high risk | 16 (1.39) | 4 (1.53) | 8 (3.65) |  | 41 (1.78) | 4.2 | 1.24 | ns¥ | 33 (1.93) | 3 (0.71) | 5 (2.91) |  |
| **Psychosomatic medicine** | **all** | **25 (55.56)** | **10 (22.22)** | **10 (22.22)** |  | **25 (0.2)** | **5.29** | **1.33** |  | **14 (56)** | **5 (20)** | **6 (24)** |  |
|  | low risk | 21 (84) | 7 (70) | 10 (100) | nsꬸ | 21 (84) | 5.37 | 1.37 | <0.001† | 11 (78.57) | 4 (80) | 6 (100) | nsꬸ |
|  | indeterminate risk | 4 (16) | 2 (20) | 0 (0) |  | 4 (16) | 4.84 | 1.16 | ns◊ | 3 (21.43) | 1 (20) | 0 (0) |  |
|  | high risk | 0 (0) | 1 (10) | 0 (0) |  | 0 (0) | na | na | ns¥ | 0 (0) | 0 (0) | 0 (0) |  |

**SD, standard deviation; ASAT, aspartate aminotransferase; ALAT, alanine aminotransferase; HbA1c, glycosylated hemoglobin; ns, not significant; na, not applicable.**

Analysis of variance (ANOVA) and Bonferroni posthoc: † p low risk vs. intermediate risk of ≥ F3 fibrosis; ◊ p low risk vs. high risk of ≥ F3 fibrosis; ¥ intermediate risk vs. high risk of ≥ F3 fibrosis.

ꬸChi-square test.

Low risk of ≥ F3 fibrosis: <1.3 (<65 years) and <2.0 (≥65 years); Intermediate risk of ≥ F3 fibrosis: ≥1.3 to ≤2.67 (<65 years) ≥2.0 to ≤2.67 (≥65 years); High risk of ≥ F3 fibrosis: >2.67.

^α^encompasses secondary hospital with heterogenous patient cohorts.

^‡^encompasses the following clinics: Ophthalmology, Infectiology, Hematology and Central hematological Laboratory, Pneumology & Allergology, Medical and radio oncology & Nuclear medicine, Anesthesiology and Pain management, Nephrology & Hypertension, Urology, Otorhinolaryngology, Osteoporosis, General internal medicine, Dermatology & Venereology, Rheumatology & Immunology, Neurology & Neurosurgery and Obstetrics and Gynaecology.

^§^encompasses the following clinics: Angiology & Vascular surgery, Visceral surgery and medicine, Orthopaedic surgery and Thoracic surgery.

# 5. **Table C.** Cohort and clinical laboratory parameters over all 26 clinics individually by F3 fibrosis risk strata, using an upper FIB-4 cut-off value of 2.67.

|  |  | **N or n (%)** | **Female;  n (%)** | | **Age,  years** | | | **Age group;  n (%)** | | | | **HbA1c;  N or n (%)** | **HbA1c,  mmol/mol** | | |
| --- | --- | --- | --- | --- | --- | --- | --- | --- | --- | --- | --- | --- | --- | --- | --- |
|  |  |  |  | p-value | mean | SD | p-value | 18-34 years | 35-64 years | ≥65 years | p-value |  | mean | SD | p-value |
| **Overall** | **all** | **36,360** | **18,666 (51.34)** |  | **55.13** | **19.36** |  | **6,848 (18.8)** | **16,788 (46.2)** | **12,724 (35.0)** |  | **12,229** | **40.21** | **12.22** |  |
|  | low risk | 26,245 (72.18) | 14,301 (54.49) | <0.001ꬸ | 50.4 | 18.72 | <0.001† <0.001◊ <0.001¥ | 6,579 (25.07 ) | 12,380 (47.17) | 7,286 (27.76) | <0.001ꬸ | 8,464 (69.21) | 39.73 | 12.13 | <0.001† <0.001◊ ns¥ |
|  | indeterminate risk | 6,202 ( 17.06) | 2,760 (44.50) |  | 62.94 | 14.48 |  | 218 (3.51 ) | 3,666 (59.11) | 2,318 (37.38) |  | 2,323 (19) | 41.16 | 12.17 |  |
|  | high risk | 3,913 (10.76) | 1,605 (41.02) |  | 74.46 | 13.46 |  | 51 (1.3) | 742 (18.96) | 3,120 (79.73) |  | 1,442 (11.79) | 41.57 | 12.59 |  |
| **Angiology & Vascular surgery** | **all** | **31 (0.09)** | **15 (48.39)** |  | **49.71** | **19.31** |  | **7 (22.58)** | **16 (51.61)** | **8 (25.81)** |  | **19 (0.16)** | **36.77** | **6.03** |  |
|  | low risk | 24 (77.42) | 14 (58.33) | nsꬸ | 46.54 | 19.08 | ns† <0.05◊ ns¥ | 7 (29.17) | 12 (50) | 5 (20.83) | <0.05ꬸ | 13 (68.42) | 35.25 | 6.2 | ns† ns◊ ns¥ |
|  | indeterminate risk | 4 (12.9) | 1 (25) |  | 48.75 | 11.53 |  | 0 (0) | 4 (100) | 0 (0) |  | 3 (15.79) | 42.79 | 1.67 |  |
|  | high risk | 3 (9.68) | 0 (0) |  | 76.33 | 4.73 |  | 0 (0) | 0 (0) | 3 (100) |  | 3 (15.79) | 37.33 | 4.93 |  |
| **Cardiology** | **all** | **1,182 (3.25)** | **284 (24.03)** |  | **60.31** | **17.29** |  | **125 (10.58)** | **508 (42.98)** | **549 (46.45)** |  | **1041 (8.51)** | **40.82** | **9.91** |  |
|  | low risk | 716 (60.58) | 202 (28.21) | <0.001ꬸ | 55.21 | 17.77 | <0.001† <0.001◊ <0.001¥ | 115 (16.06) | 319 (44.55) | 282 (39.39) | <0.001ꬸ | 637 (61.19) | 40.61 | 9.58 | ns† ns◊ ns¥ |
|  | indeterminate risk | 315 (26.65) | 57 (18.1) |  | 63.97 | 12.9 |  | 10 (3.17) | 175 (55.56) | 130 (41.27) |  | 270 (25.94) | 41.25 | 11.44 |  |
|  | high risk | 151 (12.77) | 25 (16.56) |  | 76.91 | 8.44 |  | 0 (0) | 14 (9.27) | 137 (90.73) |  | 134 (12.87) | 40.99 | 8.01 |  |
| **Neurology & Neurosurgery** | **all** | **1,800 (4.95)** | **1,121 (62.28)** |  | **46.9** | **16.12** |  | **494 (27.44)** | **1032 (57.33)** | **274 (15.22)** |  | **561 (4.59)** | **36.79** | **7.77** |  |
|  | low risk | 1551 (86.17) | 989 (63.77) | <0.05ꬸ | 44.65 | 15.55 | <0.001† <0.001◊ <0.001¥ | 489 (31.53) | 851 (54.87) | 211 (13.6) | <0.001ꬸ | 470 (83.78) | 36.42 | 7.69 | ns† ns◊ ns¥ |
|  | indeterminate risk | 217 (12.06) | 114 (52.53) |  | 58.85 | 10.87 |  | 5 (2.3) | 175 (80.65) | 37 (17.05) |  | 74 (13.19) | 38.21 | 8.6 |  |
|  | high risk | 32 (1.78) | 18 (56.25) |  | 74.78 | 9.93 |  | 0 (0) | 6 (18.75) | 26 (81.25) |  | 17 (3.03) | 40.78 | 3.71 |  |
| **Psychosomatic medicine** | **all** | **237 (0.65)** | **169 (71.31)** |  | **43.55** | **15.66** |  | **78 (32.91)** | **144 (60.76)** | **15 (6.33)** |  | **45 (0.37)** | **42.33** | **13.84** |  |
|  | low risk | 208 (87.76) | 151 (72.6) | nsꬸ | 41.9 | 15.52 | <0.001† ns◊ ns¥ | 76 (36.54) | 119 (57.21) | 13 (6.25) | <0.05ꬸ | 38 (84.44) | 43.3 | 14.78 | ns† ns◊ ns¥ |
|  | indeterminate risk | 25 (10.55) | 15 (60) |  | 55.4 | 11.09 |  | 2 (8) | 22 (88) | 1 (4) |  | 6 (13.33) | 36.23 | 4.19 |  |
|  | high risk | 4 (1.69) | 3 (75) |  | 55.25 | 12.58 |  | 0 (0) | 3 (75) | 1 (25) |  | 1 (2.22) | 42.06 | na |  |
| **Ophthalmology** | **all** | **62 (0.17)** | **27 (43.55)** |  | **62.89** | **19.27** |  | **8 (12.9)** | **21 (33.87)** | **33 (53.23)** |  | **17 (0.14)** | **44.12** | **15.26** |  |
|  | low risk | 40 (64.52) | 20 (50) | nsꬸ | 58.4 | 20.92 | ns† <0.05◊ ns¥ | 8 (20) | 13 (32.5) | 19 (47.5) | <0.05ꬸ | 9 (52.94) | 44.61 | 17.85 | ns† ns◊ ns¥ |
|  | indeterminate risk | 13 (20.97) | 5 (38.46) |  | 67.15 | 13.11 |  | 0 (0) | 7 (53.85) | 6 (46.15) |  | 6 (35.29) | 43.89 | 14.41 |  |
|  | high risk | 9 (14.52) | 2 (22.22) |  | 76.67 | 9.81 |  | 0 (0) | 1 (11.11) | 8 (88.89) |  | 2 (11.76) | 42.61 | 11.59 |  |
| **Obstetrics and Gynaecology** | **all** | **1,629 (4.48)** | **1,589 (97.54)** |  | **43.42** | **15.37** |  | **576 (35.36)** | **879 (53.96)** | **174 (10.68)** |  | **481 (3.93)** | **34.54** | **7.03** |  |
|  | low risk | 1423 (87.35) | 1387 (97.47) | nsꬸ | 42.08 | 14.97 | <0.001† <0.001◊ <0.05¥ | 547 (38.44) | 738 (51.86) | 138 (9.7) | <0.001ꬸ | 421 (87.53) | 34.52 | 7.11 | ns† ns◊ ns¥ |
|  | indeterminate risk | 176 (10.8) | 173 (98.3) |  | 51.54 | 14.23 |  | 25 (14.2) | 128 (72.73) | 23 (13.07) |  | 53 (11.02) | 34.89 | 6.32 |  |
|  | high risk | 30 (1.84) | 29 (96.67) |  | 59.23 | 17.44 |  | 4 (13.33) | 13 (43.33) | 13 (43.33) |  | 7 (1.46) | 33.16 | 8.26 |  |
| **Anesthesiology and  Pain management** | **all** | **24 (0.07)** | **13 (54.17)** |  | **58.17** | **18.03** |  | **3 (12.5)** | **13 (54.17)** | **8 (33.33)** |  | **6 (0.05)** | **43.7** | **14.3** |  |
|  | low risk | 18 (75) | 12 (66.67) | nsꬸ | 53 | 16.25 | ns† <0.05◊ ns¥ | 3 (16.67) | 11 (61.11) | 4 (22.22) | <0.05ꬸ | 2 (33.33) | 39.33 | 8.5 | ns† ns◊ ns¥ |
|  | indeterminate risk | 2 (8.33) | 0 (0) |  | 57.5 | 4.95 |  | 0 (0) | 2 (100) | 0 (0) |  | 1 (16.67) | 34.41 | na |  |
|  | high risk | 4 (16.67) | 1 (25) |  | 81.75 | 9.22 |  | 0 (0) | 0 (0) | 4 (100) |  | 3 (50) | 49.72 | 18.93 |  |
| **Infectiology** | **all** | **990 (2.72)** | **334 (33.74)** |  | **50.81** | **13.06** |  | **115 (11.62)** | **742 (74.95)** | **133 (13.43)** |  | **224 (1.83)** | **40.11** | **11.96** |  |
|  | low risk | 699 (70.61) | 247 (35.34) | nsꬸ | 48.01 | 13.31 | <0.001† <0.001◊ ns¥ | 110 (15.74) | 497 (71.1) | 92 (13.16) | <0.001ꬸ | 144 (64.29) | 40.1 | 11.91 | ns† ns◊ ns¥ |
|  | indeterminate risk | 254 (25.66) | 78 (30.71) |  | 56.93 | 9.35 |  | 4 (1.57) | 222 (87.4) | 28 (11.02) |  | 65 (29.02) | 39.46 | 8.96 |  |
|  | high risk | 37 (3.74) | 9 (24.32) |  | 61.57 | 10.14 |  | 1 (2.7) | 23 (62.16) | 13 (35.14) |  | 15 (6.7) | 43.08 | 21.18 |  |
| **Emergency department** | **all** | **6,725 (18.5)** | **3,058 (45.47)** |  | **54.75** | **20.69** |  | **1489 (22.14)** | **2767 (41.14)** | **2469 (36.71)** |  | **1299 (10.62)** | **42.68** | **14.61** |  |
|  | low risk | 4231 (62.91) | 2096 (49.54) | <0.001ꬸ | 47.98 | 20.2 | <0.001† <0.001◊ <0.001¥ | 1389 (32.83) | 1715 (40.53) | 1127 (26.64) | <0.001ꬸ | 699 (53.81) | 43.37 | 16.32 | ns† ns◊ ns¥ |
|  | indeterminate risk | 1229 (18.28) | 495 (40.28) |  | 61.52 | 15.81 |  | 73 (5.94) | 708 (57.61) | 448 (36.45) |  | 284 (21.86) | 41.64 | 12.15 |  |
|  | high risk | 1265 (18.81) | 467 (36.92) |  | 70.82 | 14.54 |  | 27 (2.13) | 344 (27.19) | 894 (70.67) |  | 316 (24.33) | 42.09 | 12.42 |  |
| **Medical and radio oncology  & Nuclear medicine** | **all** | **2,668 (7.34)** | **922 (34.56)** |  | **61.43** | **15.32** |  | **204 (7.65)** | **1147 (42.99)** | **1317 (49.36)** |  | **519 (4.24)** | **39.81** | **11.14** |  |
|  | low risk | 1993 (74.7) | 711 (35.67) | <0.05ꬸ | 59.75 | 15.97 | <0.001† <0.001◊ <0.001¥ | 197 (9.88) | 834 (41.85) | 962 (48.27) | <0.001ꬸ | 368 (70.91) | 40.65 | 10.97 | ns† ns◊ ns¥ |
|  | indeterminate risk | 426 (15.97) | 151 (35.45) |  | 63.87 | 12.01 |  | 6 (1.41) | 254 (59.62) | 166 (38.97) |  | 85 (16.38) | 37.73 | 10.68 |  |
|  | high risk | 249 (9.33) | 60 (24.1) |  | 70.63 | 10.5 |  | 1 (0.4) | 59 (23.69) | 189 (75.9) |  | 66 (12.72) | 37.81 | 12.14 |  |
| **Otorhinolaryngology** | **all** | **20 (0.06)** | **10 (50)** |  | **59.25** | **15.24** |  | **2 (10)** | **9 (45)** | **9 (45)** |  | **3 (0.02)** | **34.78** | **8.84** |  |
|  | low risk | 16 (80) | 8 (50) | nsꬸ | 59 | 16.37 | ns† ns◊ ns¥ | 2 (12.5) | 6 (37.5) | 8 (50) | nsꬸ | 3 (100) | 34.78 | 8.84 | na |
|  | indeterminate risk | 3 (15) | 2 (66.67) |  | 55.33 | 7.09 |  | 0 (0) | 3 (100) | 0 (0) |  | 0 (0) | na | na |  |
|  | high risk | 1 (5) | 0 (0) |  | 75 |  |  | 0 (0) | 0 (0) | 1 (100) |  | 0 (0) | na | na |  |
| **Orthopaedic surgery** | **all** | **74 (0.2)** | **35 (47.3)** |  | **59.93** | **18.66** |  | **9 (12.16)** | **29 (39.19)** | **36 (48.65)** |  | **8 (0.07)** | **37.83** | **5.86** |  |
|  | low risk | 62 (83.78) | 31 (50) | nsꬸ | 58.84 | 18.99 | ns† ns◊ ns¥ | 9 (14.52) | 24 (38.71) | 29 (46.77) | nsꬸ | 5 (62.5) | 36.82 | 6.8 | ns† ns◊ ns¥ |
|  | indeterminate risk | 9 (12.16) | 4 (44.44) |  | 60.56 | 15.64 |  | 0 (0) | 5 (55.56) | 4 (44.44) |  | 1 (12.5) | 40.97 | na |  |
|  | high risk | 3 (4.05) | 0 (0) |  | 80.67 | 6.51 |  | 0 (0) | 0 (0) | 3 (100) |  | 2 (25) | 38.78 | 6.18 |  |
| **Osteoporosis** | **all** | **6 (0.02)** | **2 (33.33)** |  | **61** | **14.89** |  | **0 (0)** | **3 (50)** | **3 (50)** |  | **5 (0.04)** | **45.12** | **21.52** |  |
|  | low risk | 5 (83.33) | 2 (40) | nsꬸ | 60 | 16.42 | ns† | 0 (0) | 3 (60) | 2 (40) | nsꬸ | 4 (80) | 47.26 | 24.24 | ns† |
|  | indeterminate risk | 1 (16.67) | 0 (0) |  | 66 | na |  | 0 (0) | 1 (100) | 1 (100) |  | 1 (20) | 36.6 | na |  |
|  | high risk | 0 (0) | 0 (0) |  | na | na |  | 0 (0) | 0 (0) | 0 (0) |  | na | na | na |  |
| **General internal medicine** | **all** | **762 (2.1)** | **407 (53.41)** |  | **49.84** | **16.36** |  | **165 (21.65)** | **447 (58.66)** | **150 (19.69)** |  | **450 (3.68)** | **39.03** | **8.77** |  |
|  | low risk | 637 (83.6) | 356 (55.89) | <0.05ꬸ | 47.45 | 15.93 | <0.001† <0.001◊ ns¥ | 163 (25.59) | 368 (57.77) | 106 (16.64) | <0.001ꬸ | 353 (78.44) | 38.61 | 8.28 | <0.05† ns◊ ns¥ |
|  | indeterminate risk | 93 (12.2) | 38 (40.86) |  | 61.11 | 12.83 |  | 2 (2.15) | 67 (72.04) | 24 (25.81) |  | 74 (16.44) | 41.3 | 10.49 |  |
|  | high risk | 32 (4.2) | 13 (40.63) |  | 64.56 | 12.61 |  | 0 (0) | 12 (37.5) | 20 (62.5) |  | 23 (5.11) | 38.26 | 9.37 |  |
| **Hematology and Central  hematological Laboratory** | **all** | **1,139 (3.13)** | **552 (48.46)** |  | **55.34** | **18.75** |  | **214 (18.79)** | **486 (42.67)** | **439 (38.54)** |  | **240 (1.96)** | **37.29** | **9.42** |  |
|  | low risk | 817 (71.73) | 430 (52.63) | <0.001ꬸ | 51.59 | 18.9 | <0.001† <0.001◊ <0.001¥ | 201 (24.6) | 349 (42.72) | 267 (32.68) | <0.001ꬸ | 166 (69.17) | 36.72 | 9.1 | ns† ns◊ ns¥ |
|  | indeterminate risk | 167 (14.66) | 67 (40.12) |  | 60.37 | 13.89 |  | 9 (5.39) | 101 (60.48) | 57 (34.13) |  | 43 (17.92) | 38.63 | 8.54 |  |
|  | high risk | 155 (13.61) | 55 (35.48) |  | 69.68 | 13.73 |  | 4 (2.58) | 36 (23.23) | 115 (74.19) |  | 31 (12.92) | 38.47 | 11.98 |  |
| **Rheumatology & Immunology** | **all** | **2,313 (6.36)** | **1,495 (64.63)** |  | **52.28** | **16.58** |  | **412 (17.81)** | **1304 (56.38)** | **597 (25.81)** |  | **351 (2.87)** | **39.01** | **9.34** |  |
|  | low risk | 1953 (84.44) | 1293 (66.21) | <0.05ꬸ | 50.31 | 16.55 | <0.001† <0.001◊ <0.001¥ | 406 (20.79) | 1091 (55.86) | 456 (23.35) | <0.001ꬸ | 281 (80.06) | 38.96 | 9.64 | ns† ns◊ ns¥ |
|  | indeterminate risk | 293 (12.67) | 165 (56.31) |  | 61.38 | 11.64 |  | 5 (1.71) | 196 (66.89) | 92 (31.4) |  | 53 (15.1) | 38.5 | 7.39 |  |
|  | high risk | 67 (2.9) | 37 (55.22) |  | 70.06 | 11.11 |  | 1 (1.49) | 17 (25.37) | 49 (73.13) |  | 17 (4.84) | 41.42 | 9.83 |  |
| **Pneumology & Allergology** | **all** | **560 (1.54)** | **258 (46.07)** |  | **56.77** | **16.71** |  | **70 (12.5)** | **274 (48.93)** | **216 (38.57)** |  | **139 (1.14)** | **40.81** | **9.32** |  |
|  | low risk | 413 (73.75) | 203 (49.15) | nsꬸ | 53.4 | 16.95 | <0.001† <0.001◊ <0.05¥ | 69 (16.71) | 210 (50.85) | 134 (32.45) | <0.001ꬸ | 94 (67.63) | 39.62 | 7.47 | <0.05† ns◊ ns¥ |
|  | indeterminate risk | 112 (20) | 43 (38.39) |  | 63.64 | 11.6 |  | 1 (0.89) | 61 (54.46) | 50 (44.64) |  | 31 (22.3) | 45.06 | 12.95 |  |
|  | high risk | 35 (6.25) | 12 (34.29) |  | 74.51 | 7.01 |  | 0 (0) | 3 (8.57) | 32 (91.43) |  | 14 (10.07) | 39.41 | 9.02 |  |
| **Thoracic surgery** | **all** | **343 (0.94)** | **144 (41.98)** |  | **49.56** | **18.73** |  | **96 (27.99)** | **150 (43.73)** | **97 (28.28)** |  | **33 (0.27)** | **36.07** | **6.97** |  |
|  | low risk | 296 (86.3) | 131 (44.26) | nsꬸ | 47.21 | 18.44 | <0.001† <0.05◊ ns¥ | 95 (32.09) | 129 (43.58) | 72 (24.32) | <0.001ꬸ | 25 (75.76) | 35.07 | 6.22 | ns† ns◊ ns¥ |
|  | indeterminate risk | 38 (11.08) | 12 (31.58) |  | 63.05 | 12.82 |  | 1 (2.63) | 18 (47.37) | 19 (50) |  | 5 (15.15) | 41.85 | 9.82 |  |
|  | high risk | 9 (2.62) | 1 (11.11) |  | 70.11 | 12.41 |  | 0 (0) | 3 (33.33) | 6 (66.67) |  | 3 (9.09) | 34.78 | 4.55 |  |
| **Dermatology & Venereology** | **all** | **1,250 (3.44)** | **598 (47.84)** |  | **50.84** | **18.52** |  | **305 (24.4)** | **614 (49.12)** | **331 (26.48)** |  | **251 (2.05)** | **40.71** | **11.27** |  |
|  | low risk | 1048 (83.84) | 526 (50.19) | <0.05ꬸ | 48.11 | 18.2 | <0.001† <0.001◊ <0.001¥ | 301 (28.72) | 507 (48.38) | 240 (22.9) | <0.001ꬸ | 194 (77.29) | 40.57 | 11.02 | ns† ns◊ ns¥ |
|  | indeterminate risk | 167 (13.36) | 59 (35.33) |  | 62.74 | 12.32 |  | 4 (2.4) | 104 (62.28) | 59 (35.33) |  | 46 (18.33) | 41.92 | 12.96 |  |
|  | high risk | 35 (2.8) | 13 (37.14) |  | 75.86 | 9.33 |  | 0 (0) | 3 (8.57) | 32 (91.43) |  | 11 (4.38) | 38.29 | 7.84 |  |
| **Diabetology, Endocrinology,  Nutritional medicine  & Metabolism** | **all** | **2,596 (7.14)** | **1,453 (55.97)** |  | **48.91** | **15.97** |  | **561 (21.61)** | **1567 (60.36)** | **468 (18.03)** |  | **1628 (13.31)** | **39.44** | **13.03** |  |
|  | low risk | 2252 (86.75) | 1325 (58.84) | <0.001ꬸ | 47.1 | 15.86 | <0.001† <0.001◊ <0.05¥ | 555 (24.64) | 1325 (58.84) | 372 (16.52) | <0.001ꬸ | 1435 (88.14) | 38.89 | 12.45 | <0.05† <0.001◊ ns¥ |
|  | indeterminate risk | 292 (11.25) | 116 (39.73) |  | 59.48 | 9.27 |  | 3 (1.03) | 227 (77.74) | 62 (21.23) |  | 165 (10.14) | 43.14 | 15.63 |  |
|  | high risk | 52 (2) | 12 (23.08) |  | 67.9 | 15.3 |  | 3 (5.77) | 15 (28.85) | 34 (65.38) |  | 28 (1.72) | 46.01 | 19.16 |  |
| **Visceral surgery and medicine** | **all** | **1,329 (3.66)** | **767 (57.71)** |  | **48.37** | **17** |  | **362 (27.24)** | **700 (52.67)** | **267 (20.09)** |  | **2329 (19.04)** | **38.79** | **10.31** |  |
|  | low risk | 1095 (82.39) | 672 (61.37) | <0.001ꬸ | 45.16 | 16.25 | <0.001† <0.001◊ <0.001¥ | 358 (32.69) | 562 (51.32) | 175 (15.98) | <0.001ꬸ | 1433 (61.53) | 38.13 | 9.31 | <0.05† ns◊ ns¥ |
|  | indeterminate risk | 159 (11.96) | 75 (47.17) |  | 60.6 | 11.25 |  | 3 (1.89) | 115 (72.33) | 41 (25.79) |  | 498 (21.38) | 40.45 | 12.37 |  |
|  | high risk | 75 (5.64) | 20 (26.67) |  | 69.37 | 9.56 |  | 1 (1.33) | 23 (30.67) | 51 (68) |  | 398 (17.09) | 39.1 | 10.71 |  |
| **Urology** | **all** | **39 (0.11)** | **10 (25.64)** |  | **65.49** | **14.49** |  | **1 (2.56)** | **15 (38.46)** | **23 (58.97)** |  | **9 (0.07)** | **36.96** | **5.1** |  |
|  | low risk | 31 (79.49) | 10 (32.26) | nsꬸ | 63.81 | 14.83 | ns† ns◊ ns¥ | 1 (3.23) | 13 (41.94) | 17 (54.84) | nsꬸ | 5 (55.56) | 35.29 | 4.79 | ns† ns◊ ns¥ |
|  | indeterminate risk | 5 (12.82) | 0 (0) |  | 65 | 7.71 |  | 0 (0) | 2 (40) | 3 (60) |  | 3 (33.33) | 39.51 | 6.41 |  |
|  | high risk | 3 (7.69) | 0 (0) |  | 83.67 | 5.51 |  | 0 (0) | 0 (0) | 3 (100) |  | 1 (11.11) | 37.69 | na |  |
| **Nephrology & Hypertension** | **all** | **1,615 (4.44)** | **670 (41.49)** |  | **55.69** | **16.59** |  | **229 (14.18)** | **830 (51.39)** | **556 (34.43)** |  | **1383 (11.31)** | **39.49** | **11.09** |  |
|  | low risk | 1248 (77.28) | 537 (43.03) | <0.05ꬸ | 53.42 | 17.11 | <0.001† <0.001◊ <0.001¥ | 222 (17.79) | 617 (49.44) | 409 (32.77) | <0.001ꬸ | 1058 (76.5) | 39.48 | 11.47 | ns† ns◊ ns¥ |
|  | indeterminate risk | 290 (17.96) | 110 (37.93) |  | 61.21 | 10.75 |  | 5 (1.72) | 198 (68.28) | 87 (30) |  | 260 (18.8) | 40 | 10.17 |  |
|  | high risk | 77 (4.77) | 23 (29.87) |  | 71.58 | 12.29 |  | 2 (2.6) | 15 (19.48) | 60 (77.92) |  | 65 (4.7) | 37.56 | 7.84 |  |
| **Secondary hospital I^†^** | **all** | **5,152 (14.17)** | **2702 (52.45)** |  | **61.91** | **20.82** |  | **730 (14.17)** | **1809 (35.11)** | **2613 (50.72)** |  | **643 (5.25)** | **47.35** | **17.2** |  |
|  | low risk | 3303 (64.11) | 1761 (53.32) | nsꬸ | 55.56 | 21 | <0.001† <0.001◊ <0.001¥ | 704 (21.31) | 1271 (38.48) | 1328 (40.21) | <0.001ꬸ | 227 (35.3) | 48.86 | 19.59 | 0.05† ns◊ ns¥ |
|  | indeterminate risk | 1056 (20.34) | 539 (51.04) |  | 68.63 | 15.31 |  | 22 (2.08) | 462 (43.75) | 572 (54.17) |  | 205 (31.9) | 44.4 | 12.92 |  |
|  | high risk | 793 (15.39) | 402 (50.69) |  | 79.46 | 11.59 |  | 4 (0.5) | 76 (9.58) | 713 (89.91) |  | 211 (32.81) | 47.11 | 14.57 |  |
| **Secondary hospital II**^‡^ | **all** | **3,646 (10.03)** | **1,938 (53.15)** |  | **61.05** | **21.51** |  | **590 (16.18)** | **1260 (34.56)** | **1796 (49.26)** |  | **505 (4.13)** | **46.96** | **17.62** |  |
|  | low risk | 2070 (56.77) | 1133 (54.73) | nsꬸ | 52.49 | 21.11 | <0.001† <0.001◊ <0.001¥ | 549 (26.52) | 783 (37.83) | 738 (35.65) | <0.001ꬸ | 237 (46.93) | 46.92 | 18.81 | ns† ns◊ ns¥ |
|  | indeterminate risk | 818 (22.44) | 420 (51.34) |  | 65.38 | 16.67 |  | 38 (4.65) | 401 (49.02) | 379 (46.33) |  | 122 (24.16) | 46.25 | 16.41 |  |
|  | high risk | 758 (20.79) | 385 (50.79) |  | 79.77 | 11.73 |  | 3 (0.4) | 76 (10.03) | 679 (89.58) |  | 146 (28.91) | 47.64 | 16.68 |  |
| **Secondary hospital  specialized in geriatrics^§^** | **all** | **168 (0.46)** | **93 (55.35)** |  | **77.57** | **13.93** |  | **3 (1.79)** | **22 (13.1)** | **143 (85.12)** |  | **40 (0.33)** | **43.7** | **12.34** |  |
|  | low risk | 96 (57.14) | 54 (56.25) | nsꬸ | 74.25 | 14.64 | ns† <0.001◊ ns¥ | 3 (3.13) | 13 (13.54) | 80 (83.33) | <0.05ꬸ | 19 (47.5) | 43.3 | 11.74 | ns† ns◊ ns¥ |
|  | indeterminate risk | 38 (22.61) | 21 (55.26) |  | 78.45 | 13.38 |  | 0 (0) | 9 (23.68) | 29 (76.32) |  | 12 (30) | 49.56 | 13.82 |  |
|  | high risk | 34 (20.24) | 18 (52.94) |  | 85.94 | 7.77 |  | 0 (0) | 0 (0) | 34 (100) |  | 9 (20.5) | 40.53 | 12.56 |  |

**Table C.** (Continued.)

|  |  | **HbA1c range;  n (%)** | | | | **Total cholesterol;  N or n (%)** | **Total cholesterol,  mmol/L** | | | **Total cholesterol range;  n (%)** | | | |
| --- | --- | --- | --- | --- | --- | --- | --- | --- | --- | --- | --- | --- | --- |
|  |  | <39 mmol/mol  (normal) | 39-47 mmol/mol  (prediabetes) | ≥48 mmol/mol  (diabetes) | p-value |  | mean | SD | p-value | <5.2 mmol/L  (healthy level) | 5.2-6.19 mmol/L  (borderline high level) | ≥6.2 mmol/L  (high level) | p-value |
| **Overall** | **all** | **7,549 (61.73)** | **2,836 (23.19)** | **1,844 (15.08)** |  | **12,649** | **4.64** | **1.22** |  | **8,778 (69.40)** | **2,630 (20.79)** | **1,241 (9.81)** |  |
|  | low risk | 5482 (72.62) | 1826 (64.39) | 1156 (62.69) | <0.001ꬸ | 9,051 (71.56) | 4.73 | 1.2 | <0.001† <0.001◊ <0.001¥ | 6115 (69.66) | 1990 (75.67) | 946 (76.23) | <0.001ꬸ |
|  | indeterminate risk | 1324 (17.54) | 605 (21.33) | 394 (21.37) |  | 2,377 (18.79) | 4.57 | 1.23 |  | 1679 (19.13) | 477 (18.14) | 221 (17.81) |  |
|  | high risk | 743 (9.84) | 405 (14.28) | 294 (15.94) |  | 1,221 (9.65) | 4.16 | 1.26 |  | 984 (11.21) | 163 (6.20) | 74 (5.96) |  |
| **Angiology & Vascular surgery** | **all** | **11 (57.89)** | **8 (42.11)** | **0 (0)** |  | **19 (0.15)** | **4.79** | **1.36** |  | **11 (57.89)** | **5 (26.32)** | **3 (15.79)** |  |
|  | low risk | 9 (81.82) | 4 (50) | 0 (0) | nsꬸ | 13 (68.42) | 4.87 | 1.27 | ns† ns◊ ns¥ | 7 (63.64) | 4 (80) | 2 (66.67) | nsꬸ |
|  | indeterminate risk | 0 (0) | 3 (37.5) | 0 (0) |  | 3 (15.79) | 5.16 | 0.86 |  | 2 (18.18) | 1 (20) | 0 (0) |  |
|  | high risk | 2 (18.18) | 1 (12.5) | 0 (0) |  | 3 (15.79) | 4.08 | 2.31 |  | 2 (18.18) | 0 (0) | 1 (33.33) |  |
| **Cardiology** | **all** | **562 (53.99)** | **330 (31.7)** | **149 (14.31)** |  | **999 (7.9)** | **4.25** | **1.14** |  | **800 (80.08)** | **141 (14.11)** | **58 (5.81)** |  |
|  | low risk | 347 (61.74) | 201 (60.91) | 89 (59.73) | nsꬸ | 601 (60.16) | 4.36 | 1.18 | ns† <0.001◊ <0.05¥ | 465 (58.13) | 89 (63.12) | 47 (81.03) | <0.05ꬸ |
|  | indeterminate risk | 153 (27.22) | 76 (23.03) | 41 (27.52) |  | 269 (26.93) | 4.19 | 1.04 |  | 220 (27.5) | 41 (29.08) | 8 (13.79) |  |
|  | high risk | 62 (11.03) | 53 (16.06) | 19 (12.75) |  | 129 (12.91) | 3.86 | 1 |  | 115 (14.37) | 11 (7.8) | 3 (5.17) |  |
| **Neurology & Neurosurgery** | **all** | **420 (74.87)** | **110 (19.61)** | **31 (5.53)** |  | **155 (1.23)** | **4.85** | **1.13** |  | **99 (63.87)** | **37 (23.87)** | **19 (12.26)** |  |
|  | low risk | 359 (85.48) | 85 (77.27) | 26 (83.87) | <0.05ꬸ | 123 (79.35) | 4.92 | 1.12 | ns† ns◊ ns¥ | 76 (76.77) | 30 (81.08) | 17 (89.47) | nsꬸ |
|  | indeterminate risk | 55 (13.1) | 15 (13.64) | 4 (12.9) |  | 24 (15.48) | 4.79 | 0.99 |  | 16 (16.16) | 7 (18.92) | 1 (5.26) |  |
|  | high risk | 6 (1.43) | 10 (9.09) | 1 (3.23) |  | 8 (5.16) | 4.04 | 1.43 |  | 7 (7.07) | 0 (0) | 1 (5.26) |  |
| **Psychosomatic medicine** | **all** | **25 (55.56)** | **10 (22.22)** | **10 (22.22)** |  | **25 (0.2)** | **5.29** | **1.33** |  | **14 (56)** | **5 (20)** | **6 (24)** |  |
|  | low risk | 21 (84) | 7 (70) | 10 (100) | nsꬸ | 21 (84) | 5.37 | 1.37 | ns† | 11 (78.57) | 4 (80) | 6 (100) | nsꬸ |
|  | indeterminate risk | 4 (16) | 2 (20) | 0 (0) |  | 4 (16) | 4.84 | 1.16 |  | 3 (21.43) | 1 (20) | 0 (0) |  |
|  | high risk | 0 (0) | 1 (10) | 0 (0) |  | 0 (0) | na | na |  | 0 (0) | 0 (0) | 0 (0) |  |
| **Ophthalmology** | **all** | **8 (47.06)** | **5 (29.41)** | **4 (23.53)** |  | **12 (0.09)** | **4.83** | **1.06** |  | **8 (66.67)** | **3 (25)** | **1 (8.33)** |  |
|  | low risk | 4 (50) | 3 (60) | 2 (50) | nsꬸ | 7 (58.33) | 5.12 | 1.14 | ns† ns◊ ns¥ | 4 (50) | 2 (66.67) | 1 (100) | nsꬸ |
|  | indeterminate risk | 3 (37.5) | 2 (40) | 1 (25) |  | 3 (25) | 4.62 | 1.03 |  | 2 (25) | 1 (33.33) | 0 (0) |  |
|  | high risk | 1 (12.5) | 0 (0) | 1 (25) |  | 2 (16.67) | 4.12 | 0.82 |  | 2 (25) | 0 (0) | 0 (0) |  |
| **Obstetrics and Gynaecology** | **all** | **424 (88.15)** | **40 (8.32)** | **17 (3.53)** |  | **741 (5.86)** | **5.11** | **1** |  | **409 (55.2)** | **237 (31.98)** | **95 (12.82)** |  |
|  | low risk | 371 (87.5) | 36 (90) | 14 (82.35) | nsꬸ | 655 (88.39) | 5.08 | 0.99 | ns† ns◊ ns¥ | 371 (90.71) | 207 (87.34) | 77 (81.05) | nsꬸ |
|  | indeterminate risk | 47 (11.08) | 4 (10) | 2 (11.76) |  | 79 (10.66) | 5.34 | 1.08 |  | 35 (8.56) | 28 (11.81) | 16 (16.84) |  |
|  | high risk | 6 (1.42) | 0 (0) | 1 (5.88) |  | 7 (0.94) | 5.43 | 1.14 |  | 3 (0.73) | 2 (0.84) | 2 (2.11) |  |
| **Anesthesiology and  Pain management** | **all** | **4 (66.67)** | **1 (16.67)** | **1 (16.67)** |  | **6 (0.05)** | **4.63** | **0.81** |  | **5 (83.33)** | **1 (16.67)** | **0 (0)** |  |
|  | low risk | 1 (25) | 1 (100) | 0 (0) | nsꬸ | 2 (33.33) | 4.79 | 0.27 | ns† ns◊ ns¥ | 2 (40) | 0 (0) | 0 (0) | nsꬸ |
|  | indeterminate risk | 1 (25) | 0 (0) | 0 (0) |  | 1 (16.67) | 4.13 | na |  | 1 (20) | 0 (0) | 0 (0) |  |
|  | high risk | 2 (50) | 0 (0) | 1 (100) |  | 3 (50) | 4.68 | 1.21 |  | 2 (40) | 1 (100) | 0 (0) |  |
| **Infectiology** | **all** | **138 (61.61)** | **58 (25.89)** | **28 (12.5)** |  | **797 (6.3)** | **5** | **1.13** |  | **467 (58.59)** | **218 (27.35)** | **112 (14.05)** |  |
|  | low risk | 87 (63.04) | 40 (68.97) | 17 (60.71) | nsꬸ | 548 (68.76) | 5.04 | 1.12 | ns† ns◊ ns¥ | 316 (67.67) | 155 (71.1) | 77 (68.75) | nsꬸ |
|  | indeterminate risk | 40 (28.99) | 18 (31.03) | 7 (25) |  | 216 (27.1) | 4.96 | 1.11 |  | 129 (27.62) | 56 (25.69) | 31 (27.68) |  |
|  | high risk | 11 (7.97) | 0 (0) | 4 (14.29) |  | 33 (4.14) | 4.56 | 1.28 |  | 22 (4.71) | 7 (3.21) | 4 (3.57) |  |
| **Emergency department** | **all** | **678 (52.19)** | **359 (27.64)** | **262 (20.17)** |  | **1112 (8.79)** | **4.35** | **1.28** |  | **827 (74.37)** | **195 (17.54)** | **90 (8.09)** |  |
|  | low risk | 376 (55.46) | 173 (48.19) | 150 (57.25) | nsꬸ | 601 (54.05) | 4.57 | 1.28 | <0.05† <0.001◊ <0.05¥ | 417 (50.42) | 116 (59.49) | 68 (75.56) | <0.001ꬸ |
|  | indeterminate risk | 146 (21.53) | 89 (24.79) | 49 (18.7) |  | 245 (22.03) | 4.3 | 1.2 |  | 186 (22.49) | 45 (23.08) | 14 (15.56) |  |
|  | high risk | 156 (23.01) | 97 (27.02) | 63 (24.05) |  | 266 (23.92) | 3.92 | 1.25 |  | 224 (27.09) | 34 (17.44) | 8 (8.89) |  |
| **Medical and radio oncology  & Nuclear medicine** | **all** | **317 (61.08)** | **131 (25.24)** | **71 (13.68)** |  | **574 (4.54)** | **4.72** | **1.29** |  | **385 (67.07)** | **136 (23.69)** | **53 (9.23)** |  |
|  | low risk | 213 (67.19) | 97 (74.05) | 58 (81.69) | nsꬸ | 414 (72.13) | 4.84 | 1.31 | <0.05† <0.001◊ ns¥ | 269 (69.87) | 103 (75.74) | 42 (79.25) | nsꬸ |
|  | indeterminate risk | 57 (17.98) | 21 (16.03) | 7 (9.86) |  | 97 (16.9) | 4.46 | 1.12 |  | 70 (18.18) | 21 (15.44) | 6 (11.32) |  |
|  | high risk | 47 (14.83) | 13 (9.92) | 6 (8.45) |  | 63 (10.98) | 4.35 | 1.27 |  | 46 (11.95) | 12 (8.82) | 5 (9.43) |  |
| **Otorhinolaryngology** | **all** | **2 (66.67)** | **1 (33.33)** | **0 (0)** |  | **1 (0.01)** | **8.72** | **na** |  | **0 (0)** | **0 (0)** | **1 (100)** |  |
|  | low risk | 2 (100) | 1 (100) | 0 (0) | naꬸ | 3 (100) | 8.72 | na | na | 0 (0) | 0 (0) | 1 (100) | naꬸ |
|  | indeterminate risk | 0 (0) | 0 (0) | 0 (0) |  | 0 (0) | na | na |  | 0 (0) | 0 (0) | 0 (0) |  |
|  | high risk | 0 (0) | 0 (0) | 0 (0) |  | 0 (0) | na | na |  | 0 (0) | 0 (0) | 0 (0) |  |
| **Orthopaedic surgery** | **all** | **4 (50)** | **4 (50)** | **8 (100)** |  | **8 (0.06)** | **4.79** | **0.94** |  | **6 (75)** | **1 (12.5)** | **1 (12.5)** |  |
|  | low risk | 3 (75) | 2 (50) | 5 (62.5) | nsꬸ | 5 (62.5) | 4.96 | 1.13 | ns† ns◊ ns¥ | 3 (50) | 1 (100) | 1 (100) | nsꬸ |
|  | indeterminate risk | 0 (0) | 1 (25) | 1 (12.5) |  | 1 (12.5) | 4.65 | na |  | 1 (16.67) | 0 (0) | 0 (0) |  |
|  | high risk | 1 (25) | 1 (25) | 2 (25) |  | 2 (25) | 4.44 | 0.79 |  | 2 (33.33) | 0 (0) | 0 (0) |  |
| **Osteoporosis** | **all** | **4 (80)** | **0 (0)** | **1 (20)** |  | **4 (0.03)** | **4.99** | **1.04** |  | **2 (50)** | **2 (50)** | **0 (0)** |  |
|  | low risk | 3 (75) | 0 (0) | 1 (100) | nsꬸ | 3 (75) | 4.59 | 0.82 | ns† | 2 (100) | 1 (50) | 3 (75) | nsꬸ |
|  | indeterminate risk | 1 (25) | 0 (0) | 0 (0) |  | 1 (25) | 6.18 | na |  | 0 (0) | 1 (50) | 1 (25) |  |
|  | high risk | 0 (0) | 0 (0) | 0 (0) |  | na | na | na |  | 0 (0) | 0 (0) | 0 (0) |  |
| **General internal medicine** | **all** | **294 (65.33)** | **108 (24)** | **48 (10.67)** |  | **434 (3.43)** | **5.09** | **1.26** |  | **239 (55.07)** | **129 (29.72)** | **66 (15.21)** |  |
|  | low risk | 239 (81.29) | 82 (75.93) | 32 (66.67) | nsꬸ | 346 (79.72) | 5.16 | 1.19 | ns† <0.05◊ ns¥ | 183 (76.57) | 107 (82.95) | 56 (84.85) | nsꬸ |
|  | indeterminate risk | 40 (13.61) | 21 (19.44) | 13 (27.08) |  | 69 (15.9) | 4.89 | 1.52 |  | 43 (17.99) | 17 (13.18) | 9 (13.64) |  |
|  | high risk | 15 (5.1) | 5 (4.63) | 3 (6.25) |  | 19 (4.38) | 4.36 | 1.23 |  | 13 (5.44) | 5 (3.88) | 1 (1.52) |  |
| **Hematology and Central  hematological Laboratory** | **all** | **174 (72.5)** | **43 (17.92)** | **23 (9.58)** |  | **248 (1.96)** | **4.43** | **1.17** |  | **188 (75.81)** | **41 (16.53)** | **19 (7.66)** |  |
|  | low risk | 125 (71.84) | 27 (62.79) | 14 (60.87) | nsꬸ | 166 (66.94) | 4.53 | 1.15 | ns† ns◊ ns¥ | 121 (64.36) | 32 (78.05) | 13 (68.42) | nsꬸ |
|  | indeterminate risk | 27 (15.52) | 11 (25.58) | 5 (21.74) |  | 43 (17.34) | 4.4 | 1.18 |  | 32 (17.02) | 8 (19.51) | 3 (15.79) |  |
|  | high risk | 22 (12.64) | 5 (11.63) | 4 (17.39) |  | 39 (15.73) | 4.05 | 1.15 |  | 35 (18.62) | 1 (2.44) | 3 (15.79) |  |
| **Rheumatology & Immunology** | **all** | **234 (66.67)** | **73 (20.8)** | **44 (12.54)** |  | **305 (2.41)** | **5.15** | **1.29** |  | **169 (55.41)** | **66 (21.64)** | **70 (22.95)** |  |
|  | low risk | 190 (81.2) | 57 (78.08) | 34 (77.27) | nsꬸ | 244 (80) | 5.15 | 1.29 | ns† ns◊ ns¥ | 136 (80.47) | 50 (75.76) | 58 (82.86) | nsꬸ |
|  | indeterminate risk | 34 (14.53) | 13 (17.81) | 6 (13.64) |  | 45 (14.75) | 5.09 | 1.34 |  | 25 (14.79) | 11 (16.67) | 9 (12.86) |  |
|  | high risk | 10 (4.27) | 3 (4.11) | 4 (9.09) |  | 16 (5.25) | 5.26 | 1.24 |  | 8 (4.73) | 5 (7.58) | 3 (4.29) |  |
| **Pneumology & Allergology** | **all** | **72 (51.8)** | **41 (29.5)** | **26 (18.71)** |  | **135 (1.07)** | **4.46** | **1.16** |  | **101 (74.81)** | **23 (17.04)** | **11 (8.15)** |  |
|  | low risk | 52 (72.22) | 26 (63.41) | 16 (61.54) | nsꬸ | 92 (68.15) | 4.58 | 1.13 | ns† <0.05◊ ns¥ | 68 (67.33) | 15 (65.22) | 9 (81.82) | nsꬸ |
|  | indeterminate risk | 13 (18.06) | 9 (21.95) | 9 (34.62) |  | 32 (23.7) | 4.4 | 1.29 |  | 22 (21.78) | 8 (34.78) | 2 (18.18) |  |
|  | high risk | 7 (9.72) | 6 (14.63) | 1 (3.85) |  | 11 (8.15) | 3.6 | 0.63 |  | 11 (10.89) | 0 (0) | 0 (0) |  |
| **Thoracic surgery** | **all** | **23 (69.7)** | **8 (24.24)** | **2 (6.06)** |  | **35 (0.28)** | **4.57** | **1.11** |  | **27 (77.14)** | **4 (11.43)** | **4 (11.43)** |  |
|  | low risk | 19 (82.61) | 5 (62.5) | 1 (50) | nsꬸ | 29 (82.86) | 4.51 | 1.09 | ns† ns◊ ns¥ | 23 (85.19) | 3 (75) | 3 (75) | nsꬸ |
|  | indeterminate risk | 2 (8.7) | 2 (25) | 1 (50) |  | 4 (11.43) | 5.52 | 0.98 |  | 2 (7.41) | 1 (25) | 1 (25) |  |
|  | high risk | 2 (8.7) | 1 (12.5) | 0 (0) |  | 2 (5.71) | 3.5 | 0 |  | 2 (7.41) | 0 (0) | 0 (0) |  |
| **Dermatology & Venereology** | **all** | **150 (59.76)** | **58 (23.11)** | **43 (17.13)** |  | **293 (2.32)** | **4.92** | **1.09** |  | **178 (60.75)** | **76 (25.94)** | **39 (13.31)** |  |
|  | low risk | 115 (76.67) | 47 (81.03) | 32 (74.42) | nsꬸ | 245 (83.62) | 4.92 | 1.04 | ns† ns◊ ns¥ | 150 (84.27) | 63 (82.89) | 32 (82.05) | nsꬸ |
|  | indeterminate risk | 27 (18) | 9 (15.52) | 10 (23.26) |  | 39 (13.31) | 5.06 | 1.37 |  | 21 (11.8) | 11 (14.47) | 7 (17.95) |  |
|  | high risk | 8 (5.33) | 2 (3.45) | 1 (2.33) |  | 9 (3.07) | 4.51 | 1.08 |  | 7 (3.93) | 2 (2.63) | 0 (0) |  |
| **Diabetology, Endocrinology,  Nutritional medicine  & Metabolism** | **all** | **1148 (70.52)** | **261 (16.03)** | **219 (13.45)** |  | **2304 (18.21)** | **4.52** | **1.14** |  | **1708 (74.13)** | **424 (18.4)** | **172 (7.47)** |  |
|  | low risk | 1032 (89.90) | 231 (88.51) | 172 (78.54) | <0.001ꬸ | 2016 (87.5) | 4.55 | 1.13 | <0.001† ns◊ ns¥ | 1484 (86.89) | 382 (90.09) | 150 (87.21) | nsꬸ |
|  | indeterminate risk | 100 (8.71) | 26 (9.96) | 39 (17.81) |  | 247 (10.72) | 4.29 | 1.16 |  | 191 (11.18) | 39 (9.2) | 17 (9.88) |  |
|  | high risk | 16 (1.39) | 4 (1.53) | 8 (3.65) |  | 41 (1.78) | 4.2 | 1.24 |  | 33 (1.93) | 3 (0.71) | 5 (2.91) |  |
| **Visceral surgery and medicine** | **all** | **1543 (66.25)** | **485 (20.82)** | **301 (12.92)** |  | **2236 (17.68)** | **4.68** | **1.18** |  | **1564 (69.95)** | **462 (20.66)** | **210 (9.39)** |  |
|  | low risk | 1004 (65.07) | 284 (58.56) | 145 (48.17) | <0.001ꬸ | 1408 (62.97) | 4.75 | 1.12 | ns† <0.001◊ <0.05¥ | 966 (61.76) | 305 (66.02) | 137 (65.24) | <0.001ꬸ |
|  | indeterminate risk | 309 (20.03) | 107 (22.06) | 82 (27.24) |  | 484 (21.65) | 4.77 | 1.22 |  | 323 (20.65) | 108 (23.38) | 53 (25.24) |  |
|  | high risk | 230 (14.91) | 94 (19.38) | 74 (24.58) |  | 344 (15.38) | 4.24 | 1.24 |  | 275 (17.58) | 49 (10.61) | 20 (9.52) |  |
| **Urology** | **all** | **5 (55.56)** | **4 (44.44)** | **0 (0)** |  | **11 (0.09)** | **4.63** | **1.35** |  | **7 (63.64)** | **2 (18.18)** | **2 (18.18)** |  |
|  | low risk | 3 (60) | 2 (50) | 5 (55.56) | nsꬸ | 8 (72.72) | 4.65 | 1.33 | ns† | 5 (71.43) | 2 (100) | 1 (50) | nsꬸ |
|  | indeterminate risk | 1 (20) | 2 (50) | 3 (33.33) |  | 3 (27.27) | 4.58 | 1.7 |  | 2 (28.57) | 0 (0) | 1 (50) |  |
|  | high risk | 1 (20) | 0 (0) | 1 (11.11) |  | 0 (0) | na | na |  | 0 (0) | 0 (0) | 0 (0) |  |
| **Nephrology & Hypertension** | **all** | **870 (62.91)** | **323 (23.36)** | **190 (13.74)** |  | **1351 (10.68)** | **4.62** | **1.26** |  | **950 (70.32)** | **270 (19.99)** | **131 (9.7)** |  |
|  | low risk | 672 (77.24) | 241 (74.61) | 145 (76.32) | nsꬸ | 1039 (76.91) | 4.68 | 1.25 | <0.05† ns◊ ns¥ | 714 (75.16) | 221 (81.85) | 104 (79.39) | nsꬸ |
|  | indeterminate risk | 157 (18.05) | 64 (19.81) | 39 (20.53) |  | 249 (18.43) | 4.45 | 1.25 |  | 188 (19.79) | 38 (14.07) | 23 (17.56) |  |
|  | high risk | 41 (4.71) | 18 (5.57) | 6 (3.16) |  | 63 (4.66) | 4.29 | 1.19 |  | 48 (5.05) | 11 (4.07) | 4 (3.05) |  |
| **Secondary hospital I^†^** | **all** | **227 (35.3)** | **205 (31.9)** | **211 (32.81)** |  | **478 (3.78)** | **4.52** | **1.27** |  | **349 (73.01)** | **85 (17.78)** | **44 (9.21)** |  |
|  | low risk | 122 (53.74) | 107 (52.2) | 118 (55.92) | nsꬸ | 349 (73.01) | 4.62 | 1.26 | ns† <0.05◊ ns¥ | 200 (57.31) | 58 (68.24) | 27 (61.36) | nsꬸ |
|  | indeterminate risk | 61 (26.87) | 62 (30.24) | 44 (20.85) |  | 85 (17.78) | 4.38 | 1.19 |  | 93 (26.65) | 17 (20) | 11 (25) |  |
|  | high risk | 44 (19.38) | 36 (17.56) | 49 (23.22) |  | 44 (9.21) | 4.36 | 1.39 |  | 56 (16.05) | 10 (11.76) | 6 (13.64) |  |
| **Secondary hospital II**^‡^ | **all** | **193 (38.22)** | **158 (31.29)** | **154 (30.5)** |  | **347 (2.74)** | **4.42** | **1.36** |  | **249 (71.76)** | **66 (19.02)** | **32 (9.22)** |  |
|  | low risk | 101 (52.33) | 61 (38.61) | 75 (48.7) | nsꬸ | 169 (48.7) | 4.53 | 1.26 | ns† ns◊ ns¥ | 115 (46.18) | 39 (59.09) | 15 (46.88) | nsꬸ |
|  | indeterminate risk | 45 (23.32) | 45 (28.48) | 32 (20.78) |  | 93 (26.8) | 4.4 | 1.33 |  | 67 (26.91) | 17 (25.76) | 9 (28.13) |  |
|  | high risk | 47 (24.35) | 52 (32.91) | 47 (30.52) |  | 85 (24.5) | 4.21 | 1.56 |  | 67 (26.91) | 10 (15.15) | 8 (25) |  |
| **Secondary hospital  specialized in geriatrics^§^** | **all** | **19 (47.5)** | **12 (30)** | **9 (22.5)** |  | **19 (0.15)** | **4.08** | **1.2** |  | **16 (84.21))** | **1 (5.26)** | **2 (110.52)** |  |
|  | low risk | 12 (63.16) | 6 (50) | 5 (55.56) | nsꬸ | 16 (84.21) | 4.65 | 1.36 | ns† ns◊ ns¥ | 7 (43.75) | 1 (100) | 2 (100) | nsꬸ |
|  | indeterminate risk | 1 (5.26) | 3 (25) | 3 (33.33) |  | 1 (5.26) | 3.63 | 0.65 |  | 5 (31.25) | 0 (0) | 0 (0) |  |
|  | high risk | 6 (31.58) | 3 (25) | 1 (11.11) |  | 2 (10.53) | 3.23 | 0.43 |  | 4 (25) | 0 (0) | 0 (0) |  |

**SD, standard deviation; ASAT, aspartate aminotransferase; ALAT, alanine aminotransferase; HbA1c, glycosylated hemoglobin; ns, not significant; na, not applicable.**

Analysis of variance (ANOVA) and Bonferroni posthoc: † p low risk vs. intermediate risk of ≥ F3 fibrosis; ◊ p low risk vs. high risk of ≥ F3 fibrosis; ¥ intermediate risk vs. high risk of ≥ F3 fibrosis.

ꬸChi-square test.

Low risk of ≥ F3 fibrosis: <1.3 (<65 years) and <2.0 (≥65 years); Intermediate risk of ≥ F3 fibrosis: ≥1.3 to ≤2.67 (<65 years) ≥2.0 to ≤2.67 (≥65 years); High risk of ≥ F3 fibrosis: >2.67.

^†^encompasses heterogenous patient cohorts from the hospitals Aarberg, Riggisberg and Münsingen.

‡encompasses heterogenous patient cohorts from the hospital Tiefenau.

§encompasses the nursing home Frienisberg and Seelandheim.

# 6. **Table D.** Cohort and clinical laboratory parameters over all 8 main clinic groups by F3 fibrosis risk strata, using an upper FIB-4 cut-off value of 3.25.

|  |  | **N or n (%)** | **Female;  n (%)** | | **Age; years** | | | **Age group;  n (%)** | | | | **HbA1c;  N or n (%)** | **HbA1c;  mmol/mol** | | |
| --- | --- | --- | --- | --- | --- | --- | --- | --- | --- | --- | --- | --- | --- | --- | --- |
|  |  |  |  | p-value | mean | SD | p-value | 18-34 years | 35-64 years | ≥65 years | p-value |  | mean | SD | p-value |
| **Overall** | **all** | **36,360** | **18,666 (51.34)** |  | **55.13** | **19.36** |  | **6,848 (18.8)** | **16,788 (46.2)** | **12,724 (35.0)** |  | **12,229** | **40.21** | **12.22** |  |
|  | low risk | 26,245 (72.18) | 14,301 (54.49) | <0.001ꬸ | 50.4 | 18.72 | <0.001† <0.001◊ <0.001¥ | 6,579 (25.07) | 12,380 (47.17) | 7,286 (27.76) | <0.001ꬸ | 8,464 (69.21) | 39.73 | 12.13 | <0.001† <0.001◊ ns¥ |
|  | indeterminate risk | 7,518 ( 20.68) | 3,325 (44.23) |  | 64.97 | 14.87 |  | 238 (3.17) | 3,881 (51.62) | 3,399 (45.21) |  | 2,815 (23.02) | 41.22 | 12 |  |
|  | high risk | 2,597 (7.14) | 1,040 (40.05) |  | 74.44 | 13.7 |  | 31 (1.19) | 527 (20.29) | 2,039 (78.51) |  | 950 (7.77) | 41.61 | 13.18 |  |
| **Geriatrics** | **all** | **168 (0.46)** | **93 (55.35)** |  | **77.57** | **13.93** |  | **3 (1.79)** | **22 (13.1)** | **143 (85.12)** |  | **40 (0.33)** | **43.7** | **12.34** |  |
| (secondary hospital) | low risk | 96 (57.14) | 54 (56.25) | nsꬸ | 74.25 | 14.64 | <0.05† <0.05◊ ns¥ | 3 (3.13) | 13 (13.54) | 80 (83.33) | nsꬸ | 23 (57.5) | 43.3 | 11.75 | ns† ns◊ ns¥ |
|  | indeterminate risk | 49 (29.17) | 28 (57.14) |  | 80.12 | 12.45 |  | 0 (0) | 9 (18.37) | 40 (81.63) |  | 9 (22.5) | 47.17 | 12.89 |  |
|  | high risk | 23 (13.69) | 11 (47.83) |  | 85.96 | 8.67 |  | 0 (0) | 0 (0) | 23 (100) |  | 8 (20) | 40.97 | 14.19 |  |
| **Cardiology** | **all** | **1,182 (3.25)** | **284 (24.03)** |  | **60.31** | **17.29** |  | **125 (10.58)** | **508 (42.98)** | **549 (46.45)** |  | **1,041 (8.51)** | **40.82** | **9.91** |  |
|  | low risk | 716 (60.58) | 202 (28.21) | <0.001ꬸ | 55.21 | 17.77 | <0.001† <0.001◊ <0.001¥ | 115 (16.06) | 319 (44.55) | 282 (39.39) | <0.001ꬸ | 637 (61.19) | 40.61 | 9.58 | ns† ns◊ ns¥ |
|  | indeterminate risk | 385 (32.57) | 70 (18.18) |  | 65.92 | 12.97 |  | 10 (2.6) | 186 (48.31) | 189 (49.09) |  | 331 (31.8) | 41.4 | 10.94 |  |
|  | high risk | 81 (6.85) | 12 (14.81) |  | 78.8 | 7.38 |  | 0 (0) | 3 (3.7) | 78 (96.3) |  | 73 (7.01) | 40.07 | 7.56 |  |
| **Secondary hospital*** | **all** | **8,798 (24.2)** | **4,640 (52.74)** |  | **61.56** | **21.11** |  | **1,320 (15)** | **3,069 (34.88)** | **4,409 (50.11)** |  | **1,148 (9.39)** | **47.18** | **17.38** |  |
|  | low risk | 5,373 (61.07) | 2,894 (53.86) | <0.05ꬸ | 54.37 | 21.09 | <0.001† <0.001◊ <0.001¥ | 1253 (23.32) | 2,054 (38.23) | 2,066 (38.45) | <0.001ꬸ | 584 (50.87) | 48.07 | 19.28 | ns† ns◊ ns¥ |
|  | indeterminate risk | 2,375 (26.99) | 1,237 (52.08) |  | 69.58 | 15.85 |  | 64 (2.69) | 912 (38.4) | 1,399 (58.91) |  | 378 (32.93) | 45.6 | 14.37 |  |
|  | high risk | 1,050 (11.93) | 509 (48.48) |  | 80.17 | 11.64 |  | 3 (0.29) | 103 (9.81) | 944 (89.9) |  | 186 (16.2) | 47.59 | 16.51 |  |
| **Emergency department** | **all** | **6,725 (18.5)** | **3,058 (45.47)** |  | **54.75** | **20.69** |  | **1,489 (22.14)** | **2,767 (41.14)** | **2,469 (36.71)** |  | **1,299 (10.62)** | **42.68** | **14.61** |  |
|  | low risk | 4,231 (62.91) | 2,096 (49.54) | <0.001ꬸ | 47.98 | 20.2 | <0.001† <0.001◊ <0.001¥ | 1,389 (32.83) | 1,715 (40.53) | 1,127 (26.64) | <0.001ꬸ | 699 (53.81) | 43.37 | 16.32 | ns† ns◊ ns¥ |
|  | indeterminate risk | 1,590 (23.64) | 623 (39.18) |  | 63.78 | 16.06 |  | 82 (5.16) | 790 (49.69) | 718 (45.16) |  | 369 (28.41) | 41.8 | 12.12 |  |
|  | high risk | 904 (13.44) | 339 (37.5) |  | 70.57 | 14.58 |  | 18 (1.99) | 262 (28.98) | 624 (69.03) |  | 231 (17.78) | 41.99 | 12.56 |  |
| **Medicine**** | **all** | **14,877 (40.92)** | **8,008 (53.83)** |  | **52.83** | **17.24** |  | **2,798 (18.81)** | **7,816 (52.54)** | **4,263 (28.65)** |  | **4,639 (37.93)** | **38.65** | **10.13** |  |
|  | low risk | 11,892 (79.94) | 6,731 (56.6) | <0.001ꬸ | 50.29 | 17.26 | <0.001† <0.001◊ <0.001¥ | 2,719 (22.86) | 6,108 (51.36) | 3,065 (25.77) | <0.001ꬸ | 3572 (77) | 38.46 | 10.09 | ns† ns◊ ns¥ |
|  | indeterminate risk | 2,530 (17.01) | 1,129 (44.62) |  | 61.88 | 12.78 |  | 72 (2.85) | 1,580 (62.45) | 878 (34.7) |  | 913 (19.68) | 39.35 | 9.87 |  |
|  | high risk | 455 (3.06) | 148 (32.53) |  | 68.83 | 12.28 |  | 7 (1.54) | 128 (28.13) | 320 (70.33) |  | 154 (3.32) | 38.91 | 12.31 |  |
| **Surgery***** | **all** | **1,777 (4.89)** | **961 (54.08)** |  | **49.1** | **17.59** |  | **474 (26.67)** | **895 (50.37)** | **4,08 (22.96)** |  | **2,389 (18.72)** | **38.74** | **10.24** |  |
|  | low risk | 1,477 (83.12) | 848 (57.41) | <0.001ꬸ | 46.17 | 17.09 | <0.001† <0.001◊ <0.05¥ | 469 (31.75) | 727 (49.22) | 281 (19.03) | <0.001ꬸ | 1,476 (61.78) | 38.05 | 9.24 | <0.001† ns◊ ns¥ |
|  | indeterminate risk | 242 (13.62) | 101 (41.74) |  | 62.37 | 12.08 |  | 4 (1.65) | 148 (61.16) | 90 (37.19) |  | 628 (26.29) | 40.24 | 11.94 |  |
|  | high risk | 58 (3.26) | 12 (20.69) |  | 68.66 | 10.43 |  | 1 (1.72) | 20 (34.48) | 37 (63.79) |  | 285 (11.93) | 38.97 | 10.79 |  |
| **Diabetology** | **all** | **2,596 (7.14)** | **1,453 (55.97)** |  | **48.91** | **15.97** |  | **561 (21.61)** | **1,567 (60.36)** | **468 (18.03)** |  | **1,628 (13.31)** | **39.44** | **13.03** |  |
|  | low risk | 2,252 (86.75) | 1,325 (58.84) | <0.001ꬸ | 47.1 | 15.86 | <0.001† <0.001◊ ns¥ | 555 (24.64) | 1,325 (58.84) | 372 (16.52) | <0.001ꬸ | 1,435 (88.14) | 38.89 | 12.45 | <0.001† <0.05◊ ns¥ |
|  | indeterminate risk | 321 (12.37) | 121 (37.69) |  | 60.35 | 9.99 |  | 4 (1.25) | 233 (72.59) | 84 (26.17) |  | 181 (11.12) | 43.24 | 15.62 |  |
|  | high risk | 23 (0.89) | 7 (30.43) |  | 66.3 | 18.31 |  | 2 (8.7) | 9 (39.13) | 12 (52.17) |  | 12 (0.74) | 48.35 | 23.27 |  |
| **Psychosomatic medicine** | **all** | **237 (0.65)** | **169 (71.31)** |  | **43.55** | **15.66** |  | **78 (32.91)** | **144 (60.76)** | **15 (6.33)** |  | **45 (0.37)** | **42.33** | **13.84** |  |
|  | low risk | 208 (87.76) | 151 (72.6) | nsꬸ | 41.9 | 15.52 | <0.001† ns◊ ns¥ | 76 (36.54) | 119 (57.21) | 13 (6.25) | <0.05ꬸ | 38 (84.44) | 43.3 | 14.78 | ns† ns◊ ns¥ |
|  | indeterminate risk | 26 (10.97) | 16 (61.54) |  | 54.88 | 11.18 |  | 2 (7.69) | 23 (88.46) | 1 (3.85) |  | 6 (13.33) | 36.23 | 4.19 |  |
|  | high risk | 3 (1.27) | 2 (66.67) |  | 59.67 | 10.97 |  | 0 (0) | 2 (66.67) | 1 (33.33) |  | 1 (2.22) | 42.06 |  |  |

**Table D.** (Continued.)

|  |  | **HbA1c range;  n (%)** | | | | **Total cholesterol;  N or n (%)** | **Total cholesterol;  mmol/L** | | | **Total cholesterol range;  n (%)** | | | |
| --- | --- | --- | --- | --- | --- | --- | --- | --- | --- | --- | --- | --- | --- |
|  |  | <39 mmol/mol  (normal) | 39-47 mmol/mol  (prediabetes) | ≥48 mmol/mol  (diabetes) | p-value |  | mean | SD | p-value | <5.2 mmol/L  (healthy level) | 5.2-6.19 mmol/L  (borderline high level) | ≥6.2 mmol/L  (high level) | p-value |
| **Overall** | **all** | **7,549 (61.73)** | **2,836 (23.19)** | **1,844 (15.08)** |  | **12,649** | **4.64** | **1.22** |  | **8,778 (69.40)** | **2,630 (20.79)** | **1,241 (9.81)** |  |
|  | low risk | 5,482 (72.62) | 1,826 (64.39) | 1,156 (62.69) | <0.001ꬸ | 9,051 (71.56) | 4.73 | 1.2 | <0.001† <0.001◊ <0.001¥ | 6,115 (69.66) | 1,990 (75.67) | 946 (76.23) | <0.001ꬸ |
|  | indeterminate risk | 1,566 (20.74) | 765 (26.97) | 484 (26.25) |  | 2,804 (22.17) | 4.5 | 1.24 |  | 2,022 (23.03) | 540 (20.53) | 242 (19.5) |  |
|  | high risk | 501 (6.64) | 245 (8.64) | 204 (11.06) |  | 794 (6.28) | 4.15 | 1.28 |  | 641 (7.3) | 100 (3.8) | 53 (4.27) |  |
| **Geriatrics** | **all** | **19 (47.5)** | **12 (30)** | **9 (22.5)** |  | **19 (0.15)** | **4.08** | **1.2** |  | **16 (84.21))** | **1 (5.26)** | **2 (110.52)** |  |
| (secondary hospital) | low risk | 12 (63.16) | 6 (50) | 5 (55.56) | nsꬸ | 10 (52.63) | 4.65 | 1.36 | ns† ns◊ ns¥ | 7 (43.75) | 1 (100) | 2 (100) | nsꬸ |
|  | indeterminate risk | 2 (10.53) | 4 (33.33) | 3 (33.33) |  | 6 (31.58) | 3.5 | 0.66 |  | 6 (37.5) | 0 (0) | 0 (0) |  |
|  | high risk | 5 (26.32) | 2 (16.67) | 1 (11.11) |  | 3 (15.79) | 3.36 | 0.42 |  | 3 (18.75) | 0 (0) | 0 (0) |  |
| **Cardiology** | **all** | **562 (53.99)** | **330 (31.7)** | **149 (14.31)** |  | **999 (7.9)** | **4.25** | **1.14** |  | **800 (80.08)** | **141 (14.11)** | **58 (5.81)** |  |
|  | low risk | 347 (61.74) | 201 (60.91) | 89 (59.73) | nsꬸ | 601 (60.16) | 4.36 | 1.18 | <0.05† <0.05◊ ns¥ | 465 (58.13) | 89 (63.12) | 47 (81.03) | <0.05ꬸ |
|  | indeterminate risk | 176 (31.32) | 104 (31.52) | 51 (34.23) |  | 325 (32.53) | 4.12 | 1.04 |  | 271 (33.88) | 45 (31.91) | 9 (15.52) |  |
|  | high risk | 39 (6.94) | 25 (7.58) | 9 (6.04) |  | 73 (7.31) | 3.9 | 1 |  | 64 (8) | 7 (4.96) | 2 (3.45) |  |
| **Secondary hospital**^α^ | **all** | **420 (36.59)** | **363 (31.62)** | **365 (31.79)** |  | **825 (6.5)** | **4.48** | **1.31** |  | **598 (72.48)** | **151 (18.3)** | **76 (9.21)** |  |
|  | low risk | 223 (53.1) | 168 (46.28) | 193 (52.88) | <0.05ꬸ | 454 (55.03) | 4.58 | 1.26 | <0.05† ns◊ ns¥ | 315 (52.68) | 97 (64.24) | 42 (55.26) | nsꬸ |
|  | indeterminate risk | 134 (31.9) | 140 (38.57) | 104 (28.49) |  | 270 (32.73) | 4.34 | 1.29 |  | 206 (34.45) | 41 (27.15) | 23 (30.26) |  |
|  | high risk | 63 (15) | 55 (15.15) | 68 (18.63) |  | 101 (12.24) | 4.36 | 1.5 |  | 77 (12.88) | 13 (8.61) | 11 (14.47) |  |
| **Emergency department** | **all** | **678 (52.19)** | **359 (27.64)** | **262 (20.17)** |  | **1112 (8.79)** | **4.35** | **1.28** |  | **827 (74.37)** | **195 (17.54)** | **90 (8.09)** |  |
|  | low risk | 376 (55.46) | 173 (48.19) | 150 (57.25) | nsꬸ | 601 (54.05) | 4.57 | 1.28 | <0.001† <0.001◊ ns¥ | 417 (50.42) | 116 (59.49) | 68 (75.56) | <0.001ꬸ |
|  | indeterminate risk | 186 (27.43) | 116 (32.31) | 67 (25.57) |  | 315 (28.33) | 4.19 | 1.19 |  | 247 (29.87) | 54 (27.69) | 14 (15.56) |  |
|  | high risk | 116 (17.11) | 70 (19.5) | 45 (17.18) |  | 196 (17.63) | 3.96 | 1.31 |  | 163 (19.71) | 25 (12.82) | 8 (8.89) |  |
| **Medicine**^‡^ | **all** | **3,116 (67.17)** | **996 (21.47)** | **527 (11.36)** |  | **5,067 (40.6)** | **4.85** | **1.21** |  | **3,207 (63.29)** | **1,241 (24.49)** | **619 (12.22)** |  |
|  | low risk | 2,436 (78.18) | 745 (74.8) | 391 (74.19) | nsꬸ | 3,893 (76.83) | 4.9 | 1.19 | <0.001† <0.001◊ <0.001¥ | 2417 (75.37) | 988 (79.61) | 488 (78.84) | <0.05ꬸ |
|  | indeterminate risk | 577 (18.52) | 220 (22.09) | 116 (22.01) |  | 1,015 (20.03) | 4.72 | 1.25 |  | 670 (20.89) | 228 (18.37) | 117 (18.9) |  |
|  | high risk | 103 (3.31) | 31 (3.11) | 20 (3.8) |  | 159 (3.14) | 4.31 | 1.22 |  | 120 (3.74) | 25 (2.01) | 14 (2.26) |  |
| **Surgery^§^** | **all** | **1,581 (66.18)** | **505 (21.14)** | **303 (12.68)** |  | **2,298 (18.17)** | **4.68** | **1.18** |  | **1,608 (69.97)** | **472 (20.54)** | **218 (9.49)** |  |
|  | low risk | 1,035 (65.46) | 295 (58.42) | 146 (48.18) | <0.001ꬸ | 1,455 (63.32) | 4.75 | 1.12 | ns† <0.001◊ <0.001¥ | 999 (62.13) | 313 (66.31) | 143 (65.6) | <0.05ꬸ |
|  | indeterminate risk | 378 (23.91) | 149 (29.5) | 101 (33.33) |  | 600 (26.11) | 4.7 | 1.23 |  | 410 (25.5) | 130 (27.54) | 60 (27.52) |  |
|  | high risk | 168 (10.63) | 61 (12.08) | 56 (18.48) |  | 243 (10.57) | 4.2 | 1.26 |  | 199 (12.38) | 29 (6.14) | 15 (6.88) |  |
| **Diabetology** | **all** | **1,148 (70.52)** | **261 (16.03)** | **219 (13.45)** |  | **2,304 (18.21)** | **4.52** | **1.14** |  | **1,708 (74.13)** | **424 (18.4)** | **172 (7.47)** |  |
|  | low risk | 1,032 (89.9) | 231 (88.51) | 172 (78.54) | <0.001ꬸ | 2,016 (87.5) | 4.55 | 1.13 | <0.05† ns◊ ns¥ | 1,484 (86.89) | 382 (90.09) | 150 (87.21) | nsꬸ |
|  | indeterminate risk | 109 (9.49) | 30 (11.49) | 42 (19.18) |  | 269 (11.68) | 4.28 | 1.16 |  | 209 (12.24) | 41 (9.67) | 19 (11.05) |  |
|  | high risk | 7 (0.61) | 0 (0) | 5 (2.28) |  | 19 (0.82) | 4.28 | 1.39 |  | 15 (0.88) | 1 (0.24) | 3 (1.74) |  |
| **Psychosomatic medicine** | **all** | **25 (55.56)** | **10 (22.22)** | **10 (22.22)** |  | **25 (0.2)** | **5.29** | **1.33** |  | **14 (56)** | **5 (20)** | **6 (24)** |  |
|  | low risk | 21 (84) | 7 (70) | 10 (100) | nsꬸ | 21 (84) | 5.37 | 1.37 | ns† | 11 (78.57) | 4 (80) | 6 (100) | nsꬸ |
|  | indeterminate risk | 4 (16) | 2 (20) | 0 (0) |  | 4 (16) | 4.84 | 1.16 |  | 3 (21.43) | 1 (20) | 0 (0) |  |
|  | high risk | 0 (0) | 1 (10) | 0 (0) |  | 0 (0) | na | na |  | 0 (0) | 0 (0) | 0 (0) |  |

**SD, standard deviation; ASAT, aspartate aminotransferase; ALAT, alanine aminotransferase; HbA1c, glycosylated hemoglobin; ns, not significant; na, not applicable.**

Analysis of variance (ANOVA) and Bonferroni posthoc: † p low risk vs. intermediate risk of ≥ F3 fibrosis; ◊ p low risk vs. high risk of ≥ F3 fibrosis; ¥ intermediate risk vs. high risk of ≥ F3 fibrosis.

ꬸChi-square test.

Low risk of ≥ F3 fibrosis: <1.3 (<65 years) and <2.0 (≥65 years); Intermediate risk of ≥ F3 fibrosis: ≥1.3 to ≤3.25 (<65 years) ≥2.0 to ≤3.25 (≥65 years); High risk of ≥ F3 fibrosis: >3.25.

^α^encompasses secondary hospital with heterogenous patient cohorts.

^‡^encompasses the following clinics: Ophthalmology, Infectiology, Hematology and Central hematological Laboratory, Pneumology & Allergology, Medical and radio oncology & Nuclear medicine, Anesthesiology and Pain management, Nephrology & Hypertension, Urology, Otorhinolaryngology, Osteoporosis, General internal medicine, Dermatology & Venereology, Rheumatology & Immunology, Neurology & Neurosurgery and Obstetrics and Gynaecology.

^§^encompasses the following clinics: Angiology & Vascular surgery, Visceral surgery and medicine, Orthopaedic surgery and Thoracic surgery.

# 7. **Table E.** Cohort and clinical laboratory parameters over all 26 clinics individually by F3 fibrosis risk strata, using an upper FIB-4 cut-off value of 3.25.

|  |  | **N or n (%)** | **Female;  n (%)** | | **Age,  years** | | | **Age group;  n (%)** | | | | **HbA1c;  N or n (%)** | **HbA1c,  mmol/mol** | | |
| --- | --- | --- | --- | --- | --- | --- | --- | --- | --- | --- | --- | --- | --- | --- | --- |
|  |  |  |  | p-value | mean | SD | p-value | 18-34 years | 35-64 years | ≥65 years | p-value |  | mean | SD | p-value |
| **Overall** | **all** | **36,360** | **18,666 (51.34)** |  | **55.13** | **19.36** |  | **6,848 (18.8)** | **16,788 (46.2)** | **12,724 (35.0)** |  | **12,229** | **40.21** | **12.22** |  |
|  | low risk | 26,245 (72.18) | 14,301 (54.49) | <0.001ꬸ | 50.4 | 18.72 | <0.001† <0.001◊ <0.001¥ | 6579 (25.07) | 12380 (47.17) | 7286 (27.76) | <0.001ꬸ | 8,464 (69.21) | 39.73 | 12.13 | <0.001† <0.001◊ ns¥ |
|  | indeterminate risk | 7,518 (20.68) | 3,325 (44.23) |  | 64.96 | 14.87 |  | 238 (3.17) | 3881 (51.62) | 3399 (45.21) |  | 2,815 (23.02) | 41.22 | 12.03 |  |
|  | high risk | 2,597 (7.14) | 1,040 (40.05) |  | 74.44 | 13.78 |  | 31 (1.19) | 527 (20.29) | 2039 (78.51) |  | 950 (7.77) | 41.61 | 13.18 |  |
| **Angiology & Vascular surgery** | **all** | **31 (0.09)** | **15 (48.39)** |  | **49.71** | **19.31** |  | **7 (22.58)** | **16 (51.61)** | **8 (25.81)** |  | **19 (0.16)** | **36.77** | **6.03** |  |
|  | low risk | 24 (77.42) | 14 (58.33) | nsꬸ | 46.54 | 19.08 | ns† ns◊ ns¥ | 7 (29.17) | 12 (50) | 5 (20.83) | nsꬸ | 13 (68.42) | 35.25 | 6.2 | ns† ns◊ ns¥ |
|  | indeterminate risk | 5 (16.13) | 1 (20) |  | 53.2 | 14.1 |  | 0 (0) | 4 (80) | 1 (20) |  | 4 (21.05) | 40.15 | 5.46 |  |
|  | high risk | 2 (6.45) | 0 (0) |  | 79 | 1.41 |  | 0 (0) | 0 (0) | 2 (100) |  | 2 (10.53) | 39.88 | 3.09 |  |
| **Cardiology** | **all** | **1,182 (3.25)** | **284 (24.03)** |  | **60.31** | **17.29** |  | **125 (10.58)** | **508 (42.98)** | **549 (46.45)** |  | **1041 (8.51)** | **40.82** | **9.91** |  |
|  | low risk | 716 (60.58) | 202 (28.21) | <0.001ꬸ | 55.21 | 17.77 | <0.001† <0.001◊ <0.001¥ | 115 (16.06) | 319 (44.55) | 282 (39.39) | <0.001ꬸ | 637 (61.19) | 40.61 | 9.58 | ns† ns◊ ns¥ |
|  | indeterminate risk | 385 (32.57) | 70 (18.18) |  | 65.92 | 12.97 |  | 10 (2.6) | 186 (48.31) | 189 (49.09) |  | 331 (31.8) | 41.4 | 10.94 |  |
|  | high risk | 81 (6.85) | 12 (14.81) |  | 78.8 | 7.38 |  | 0 (0) | 3 (3.7) | 78 (96.3) |  | 73 (7.01) | 40.07 | 7.56 |  |
| **Neurology & Neurosurgery** | **all** | **1,800 (4.95)** | **1,121 (62.28)** |  | **46.9** | **16.12** |  | **494 (27.44)** | **1032 (57.33)** | **274 (15.22)** |  | **561 (4.59)** | **36.79** | **7.77** |  |
|  | low risk | 1551 (86.17) | 989 (63.77) | <0.05ꬸ | 44.65 | 15.55 | <0.001† <0.001◊ <0.05¥ | 489 (31.53) | 851 (54.87) | 211 (13.6) | <0.001ꬸ | 470 (83.78) | 36.42 | 7.69 | ns† ns◊ ns¥ |
|  | indeterminate risk | 232 (12.89) | 124 (53.45) |  | 60.03 | 11.7 |  | 5 (2.16) | 177 (76.29) | 50 (21.55) |  | 82 (14.62) | 38.32 | 8.21 |  |
|  | high risk | 17 (0.94) | 8 (47.06) |  | 72.76 | 9.66 |  | 0 (0) | 4 (23.53) | 13 (76.47) |  | 9 (1.6) | 42.06 | 4.2 |  |
| **Psychosomatic medicine** | **all** | **237 (0.65)** | **169 (71.31)** |  | **43.55** | **15.66** |  | **78 (32.91)** | **144 (60.76)** | **15 (6.33)** |  | **45 (0.37)** | **42.33** | **13.84** |  |
|  | low risk | 208 (87.76) | 151 (72.6) | nsꬸ | 41.9 | 15.52 | <0.001† ns◊ ns¥ | 76 (36.54) | 119 (57.21) | 13 (6.25) | <0.05ꬸ | 38 (84.44) | 43.3 | 14.78 | ns† ns◊ ns¥ |
|  | indeterminate risk | 26 (10.97) | 16 (61.54) |  | 54.88 | 11.18 |  | 2 (7.69) | 23 (88.46) | 1 (3.85) |  | 6 (13.33) | 36.23 | 4.19 |  |
|  | high risk | 3 (1.27) | 2 (66.67) |  | 59.67 | 10.97 |  | 0 (0) | 2 (66.67) | 1 (33.33) |  | 1 (2.22) | 42.06 | na |  |
| **Ophthalmology** | **all** | **62 (0.17)** | **27 (43.55)** |  | **62.89** | **19.27** |  | **8 (12.9)** | **21 (33.87)** | **33 (53.23)** |  | **17 (0.14)** | **44.12** | **15.26** |  |
|  | low risk | 40 (64.52) | 20 (50) | nsꬸ | 58.4 | 20.92 | ns† ns◊ ns¥ | 8 (20) | 13 (32.5) | 19 (47.5) | nsꬸ | 9 (52.94) | 44.61 | 17.85 | ns† ns◊ ns¥ |
|  | indeterminate risk | 18 (29.03) | 6 (33.33) |  | 70.17 | 12.83 |  | 0 (0) | 7 (38.89) | 11 (61.11) |  | 7 (41.18) | 44.87 | 13.42 |  |
|  | high risk | 4 (6.45) | 1 (25) |  | 75 | 12.06 |  | 0 (0) | 1 (25) | 3 (75) |  | 1 (5.88) | 34.41 |  |  |
| **Obstetrics and Gynaecology** | **all** | **1,629 (4.48)** | **1,589 (97.54)** |  | **43.42** | **15.37** |  | **576 (35.36)** | **879 (53.96)** | **174 (10.68)** |  | **481 (3.93)** | **34.54** | **7.03** |  |
|  | low risk | 1423 (87.35) | 1387 (97.47) | nsꬸ | 42.08 | 14.97 | <0.001† <0.001◊ ns¥ | 547 (38.44) | 738 (51.86) | 138 (9.7) | <0.001ꬸ | 421 (87.53) | 34.52 | 7.11 | ns† ns◊ ns¥ |
|  | indeterminate risk | 187 (11.48) | 183 (97.86) |  | 52.14 | 14.68 |  | 27 (14.44) | 131 (70.05) | 29 (15.51) |  | 55 (11.43) | 34.69 | 6.29 |  |
|  | high risk | 19 (1.17) | 19 (100) |  | 57.74 | 16.91 |  | 2 (10.53) | 10 (52.63) | 7 (36.84) |  | 5 (1.04) | 34.63 | 9.64 |  |
| **Anesthesiology and  Pain management** | **all** | **24 (0.07)** | **13 (54.17)** |  | **58.17** | **18.03** |  | **3 (12.5)** | **13 (54.17)** | **8 (33.33)** |  | **6 (0.05)** | **43.7** | **14.3** |  |
|  | low risk | 18 (75) | 12 (66.67) | nsꬸ | 53 | 16.25 | ns† <0.05◊ ns¥ | 3 (16.67) | 11 (61.11) | 4 (22.22) | nsꬸ | 2 (33.33) | 39.33 | 8.5 | ns† ns◊ ns¥ |
|  | indeterminate risk | 4 (16.67) | 1 (25) |  | 67.5 | 13.03 |  | 0 (0) | 2 (50) | 2 (50) |  | 2 (33.33) | 36.6 | 3.09 |  |
|  | high risk | 2 (8.33) | 0 (0) |  | 86 | 9.9 |  | 0 (0) | 0 (0) | 2 (100) |  | 2 (33.33) | 55.18 | 23.19 |  |
| **Infectiology** | **all** | **990 (2.72)** | **334 (33.74)** |  | **50.81** | **13.06** |  | **115 (11.62)** | **742 (74.95)** | **133 (13.43)** |  | **224 (1.83)** | **40.11** | **11.96** |  |
|  | low risk | 699 (70.61) | 247 (35.34) | <0.05ꬸ | 48.01 | 13.31 | <0.001† <0.001◊ ns¥ | 110 (15.74) | 497 (71.1) | 92 (13.16) | <0.001ꬸ | 144 (64.29) | 40.1 | 11.91 | ns† ns◊ ns¥ |
|  | indeterminate risk | 267 (26.97) | 84 (31.46) |  | 57.03 | 9.27 |  | 4 (1.5) | 232 (86.89) | 31 (11.61) |  | 73 (32.59) | 39.62 | 9.68 |  |
|  | high risk | 24 (2.42) | 3 (12.50) |  | 62.88 | 11.27 |  | 1 (4.17) | 13 (54.17) | 10 (41.67) |  | 7 (3.13) | 45.5 | 27.72 |  |
| **Emergency department** | **all** | **6,725 (18.5)** | **3,058 (45.47)** |  | **54.75** | **20.69** |  | **1489 (22.14)** | **2767 (41.14)** | **2469 (36.71)** |  | **1299 (10.62)** | **42.68** | **14.61** |  |
|  | low risk | 4231 (62.91) | 2096 (49.54) | <0.001ꬸ | 47.98 | 20.2 | <0.001† <0.001◊ <0.001¥ | 1389 (32.83) | 1715 (40.53) | 1127 (26.64) | <0.001ꬸ | 699 (53.81) | 43.37 | 16.32 | ns† ns◊ ns¥ |
|  | indeterminate risk | 1590 (23.64) | 623 (39.18) |  | 63.78 | 16.06 |  | 82 (5.16) | 790 (49.69) | 718 (45.16) |  | 369 (28.41) | 41.8 | 12.12 |  |
|  | high risk | 904 (13.44) | 339 (37.50) |  | 70.57 | 14.58 |  | 18 (1.99) | 262 (28.98) | 624 (69.03) |  | 231 (17.78) | 41.99 | 12.56 |  |
| **Medical and radio oncology  & Nuclear medicine** | **all** | **2,668 (7.34)** | **922 (34.56)** |  | **61.43** | **15.32** |  | **204 (7.65)** | **1147 (42.99)** | **1317 (49.36)** |  | **519 (4.24)** | **39.81** | **11.14** |  |
|  | low risk | 1993 (74.7) | 711 (35.67) | <0.05ꬸ | 59.75 | 15.97 | <0.001† <0.001◊ <0.05¥ | 197 (9.88) | 834 (41.85) | 962 (48.27) | <0.001ꬸ | 368 (70.91) | 40.65 | 10.97 | ns† ns◊ ns¥ |
|  | indeterminate risk | 515 (19.30) | 171 (33.20) |  | 65.35 | 12.18 |  | 7 (1.36) | 271 (52.62) | 237 (46.02) |  | 108 (20.81) | 36.96 | 9.84 |  |
|  | high risk | 160 (6.00) | 40 (25) |  | 69.62 | 10.43 |  | 0 (0) | 42 (26.25) | 118 (73.75) |  | 43 (8.29) | 39.78 | 14.28 |  |
| **Otorhinolaryngology** | **all** | **20 (0.06)** | **10 (50)** |  | **59.25** | **15.24** |  | **2 (10)** | **9 (45)** | **9 (45)** |  | **3 (0.02)** | **34.78** | **8.84** |  |
|  | low risk | 16 (80) | 8 (50) | nsꬸ | 59 | 16.37 | ns† | 2 (12.5) | 6 (37.5) | 8 (50) | nsꬸ | 3 (100) | 34.78 | 8.84 | na |
|  | indeterminate risk | 4 (20) | 2 (50) |  | 60.25 | 11.41 |  | 0 (0) | 3 (75) | 1 (25) |  | 0 (0) | na | na |  |
|  | high risk | 0 (0) | na |  | na | na |  | na | na | na |  | 0 (0) | na | na |  |
| **Orthopaedic surgery** | **all** | **74 (0.2)** | **35 (47.3)** |  | **59.93** | **18.66** |  | **9 (12.16)** | **29 (39.19)** | **36 (48.65)** |  | **8 (0.07)** | **37.83** | **5.86** |  |
|  | low risk | 62 (83.78) | 31 (50) | nsꬸ | 58.84 | 18.99 | ns† | 9 (14.52) | 24 (38.71) | 29 (46.77) | nsꬸ | 5 (62.5) | 36.82 | 6.8 | ns† |
|  | indeterminate risk | 9 (16.21) | 4 (33.33 ) |  | 65.58 | 16.38 |  | 0 (0) | 5 (41.67) | 7 (58.33) |  | 3 (37.5) | 39.51 | 4.55 |  |
|  | high risk | 0 (0) | na |  | na | na |  | 0 (0) | 0 (0) | 0 (0) |  | 0 (0) | na | na |  |
| **Osteoporosis** | **all** | **6 (0.02)** | **2 (33.33)** |  | **61** | **14.89** |  | **0 (0)** | **3 (50)** | **3 (50)** |  | **5 (0.04)** | **45.12** | **21.52** |  |
|  | low risk | 5 (83.33) | 2 (40) | nsꬸ | 60 | 16.42 | ns† | 0 (0) | 3 (60) | 2 (40) | nsꬸ | 4 (80) | 47.26 | 24.24 | ns† |
|  | indeterminate risk | 1 (16.67) | 0 (0) |  | 66 | na |  | 0 (0) | 1 (100) | 1 (100) |  | 1 (20) | 36.6 | na |  |
|  | high risk | 0 (0) | na |  | na | na |  | 0 (0) | 0 (0) | 0 (0) |  | 0 (0) | na | na |  |
| **General internal medicine** | **all** | **762 (2.1)** | **407 (53.41)** |  | **49.84** | **16.36** |  | **165 (21.65)** | **447 (58.66)** | **150 (19.69)** |  | **450 (3.68)** | **39.03** | **8.77** |  |
|  | low risk | 637 (83.6) | 356 (55.89) | <0.05ꬸ | 47.45 | 15.93 | <0.001† <0.001◊ ns¥ | 163 (25.59) | 368 (57.77) | 106 (16.64) | <0.001ꬸ | 353 (78.44) | 38.61 | 8.28 | <0.05† ns◊ <0.05¥ |
|  | indeterminate risk | 101 (13.25) | 41 (40.59) |  | 61.73 | 12.81 |  | 2 (1.98) | 70 (69.31) | 29 (28.71) |  | 82 (18.22) | 41.58 | 10.45 |  |
|  | high risk | 24 (3.15) | 10 (41.67) |  | 63.08 | 13.02 |  | 0 (0) | 9 (37.5) | 15 (62.5) |  | 15 (3.33) | 35.07 | 7.28 |  |
| **Hematology and Central  hematological Laboratory** | **all** | **1,139 (3.13)** | **552 (48.46)** |  | **55.34** | **18.75** |  | **214 (18.79)** | **486 (42.67)** | **439 (38.54)** |  | **240 (1.96)** | **37.29** | **9.42** |  |
|  | low risk | 817 (71.73) | 430 (52.63) | <0.001ꬸ | 51.59 | 18.9 | <0.001† <0.001◊ <0.05¥ | 201 (24.6) | 349 (42.72) | 267 (32.68) | <0.001ꬸ | 166 (69.17) | 36.72 | 9.1 | ns† ns◊ ns¥ |
|  | indeterminate risk | 217 (19.05) | 91 (41.94) |  | 62.76 | 14.47 |  | 10 (4.61) | 110 (50.69) | 97 (44.7) |  | 50 (20.83) | 38.24 | 8.34 |  |
|  | high risk | 105 (9.22) | 31 (29.52) |  | 69.18 | 13.83 |  | 3 (2.86) | 27 (25.71) | 75 (71.43) |  | 24 (10) | 39.24 | 13.08 |  |
| **Rheumatology & Immunology** | **all** | **2,313 (6.36)** | **1,495 (64.63)** |  | **52.28** | **16.58** |  | **412 (17.81)** | **1304 (56.38)** | **597 (25.81)** |  | **351 (2.87)** | **39.01** | **9.34** |  |
|  | low risk | 1953 (84.44) | 1293 (66.21) | <0.05ꬸ | 50.31 | 16.55 | <0.001† <0.001◊ ns¥ | 406 (20.79) | 1091 (55.86) | 456 (23.35) | <0.001ꬸ | 281 (80.06) | 38.96 | 9.64 | ns† ns◊ ns¥ |
|  | indeterminate risk | 332 (14.35) | 184 (55.42) |  | 62.73 | 12.14 |  | 6 (1.81) | 202 (60.84) | 124 (37.35) |  | 63 (17.95) | 39.18 | 8.32 |  |
|  | high risk | 28 (1.21) | 18 (64.29) |  | 66.11 | 10.05 |  | 0 (0) | 11 (39.29) | 17 (60.71) |  | 7 (1.99) | 39.41 | 5.88 |  |
| **Pneumology & Allergology** | **all** | **560 (1.54)** | **258 (46.07)** |  | **56.77** | **16.71** |  | **70 (12.5)** | **274 (48.93)** | **216 (38.57)** |  | **139 (1.14)** | **40.81** | **9.32** |  |
|  | low risk | 413 (73.75) | 203 (49.15) | <0.05ꬸ | 53.4 | 16.95 | <0.001† <0.001◊ ns¥ | 69 (16.71) | 210 (50.85) | 134 (32.45) | <0.001ꬸ | 94 (67.63) | 39.62 | 7.47 | <0.05† ns◊ ns¥ |
|  | indeterminate risk | 130 (23.21) | 49 (37.69) |  | 65.27 | 11.84 |  | 1 (0.77) | 62 (47.69) | 67 (51.54) |  | 40 (28.78) | 44.44 | 12.25 |  |
|  | high risk | 17 (3.04) | 6 (35.29) |  | 73.59 | 6.26 |  | 0 (0) | 2 (11.76) | 15 (88.24) |  | 5 (3.6) | 34.19 | 4.26 |  |
| **Thoracic surgery** | **all** | **343 (0.94)** | **144 (41.98)** |  | **49.56** | **18.73** |  | **96 (27.99)** | **150 (43.73)** | **97 (28.28)** |  | **33 (0.27)** | **36.07** | **6.97** |  |
|  | low risk | 296 (86.3) | 131 (44.26) | nsꬸ | 47.21 | 18.44 | <0.001† ns◊ ns¥ | 95 (32.09) | 129 (43.58) | 72 (24.32) | <0.001ꬸ | 25 (75.76) | 35.07 | 6.22 | ns† ns◊ ns¥ |
|  | indeterminate risk | 44 (12.83) | 13 (29.55) |  | 64.7 | 12.9 |  | 1 (2.27) | 19 (43.18) | 24 (54.55) |  | 7 (21.21) | 39.1 | 9.31 |  |
|  | high risk | 3 (0.87) | 0 (0) |  | 60 | 15.1 |  | 0 (0) | 2 (66.67) | 1 (33.33) |  | 1 (3.03) | 39.88 | 0 |  |
| **Dermatology & Venereology** | **all** | **1,250 (3.44)** | **598 (47.84)** |  | **50.84** | **18.52** |  | **305 (24.4)** | **614 (49.12)** | **331 (26.48)** |  | **251 (2.05)** | **40.71** | **11.27** |  |
|  | low risk | 1048 (83.84) | 526 (50.19) | <0.001ꬸ | 48.11 | 18.2 | <0.001† <0.001◊ <0.05¥ | 301 (28.72) | 507 (48.38) | 240 (22.9) | <0.001ꬸ | 194 (77.29) | 40.57 | 11.02 | ns† ns◊ ns¥ |
|  | indeterminate risk | 185 (14.80) | 68 (36.76) |  | 64.19 | 12.93 |  | 4 (2.16) | 106 (57.3) | 75 (40.54) |  | 50 (19.92) | 41.65 | 12.51 |  |
|  | high risk | 17 (1.36) | 4 (23.53) |  | 73.94 | 7.56 |  | 0 (0) | 1 (5.88) | 16 (94.12) |  | 7 (2.79) | 38.16 | 9.55 |  |
| **Diabetology, Endocrinology,  Nutritional medicine  & Metabolism** | **all** | **2,596 (7.14)** | **1,453 (55.97)** |  | **48.91** | **15.97** |  | **561 (21.61)** | **1567 (60.36)** | **468 (18.03)** |  | **1628 (13.31)** | **39.44** | **13.03** |  |
|  | low risk | 2252 (86.75) | 1325 (58.84) | <0.001ꬸ | 47.1 | 15.86 | <0.001† <0.001◊ ns¥ | 555 (24.64) | 1325 (58.84) | 372 (16.52) | <0.001ꬸ | 1,435 (88.14) | 38.89 | 12.45 | <0.001† <0.05◊ ns¥ |
|  | indeterminate risk | 321 (12.37) | 121 (37.69) |  | 60.35 | 9.99 |  | 4 (1.25) | 233 (72.59) | 84 (26.17) |  | 181 (11.12) | 43.24 | 15.62 |  |
|  | high risk | 23 (0.89) | 7 (30.43) |  | 66.3 | 18.31 |  | 2 (8.7) | 9 (39.13) | 12 (52.17) |  | 12 (0.74) | 48.35 | 23.27 |  |
| **Visceral surgery and medicine** | **all** | **1,329 (3.66)** | **767 (57.71)** |  | **48.37** | **17** |  | **362 (27.24)** | **700 (52.67)** | **267 (20.09)** |  | **2329 (19.04)** | **38.79** | **10.31** |  |
|  | low risk | 1095 (82.39) | 672 (61.37) | <0.001ꬸ | 45.16 | 16.25 | <0.001† <0.001◊ <0.05¥ | 358 (32.69) | 562 (51.32) | 175 (15.98) | <0.001ꬸ | 1,433 (61.53) | 38.13 | 9.31 | <0.001† ns◊ ns¥ |
|  | indeterminate risk | 181 (13.62) | 83 (45.86) |  | 61.85 | 11.41 |  | 3 (1.66) | 120 (66.3) | 58 (32.04) |  | 614 (26.36) | 40.26 | 12.03 |  |
|  | high risk | 53 (3.99) | 12 (22.64) |  | 68.75 | 10.1 |  | 1 (1.89) | 18 (33.96) | 34 (64.15) |  | 282 (12.11) | 38.96 | 10.84 |  |
| **Urology** | **all** | **39 (0.11)** | **10 (25.64)** |  | **65.49** | **14.49** |  | **1 (2.56)** | **15 (38.46)** | **23 (58.97)** |  | **9 (0.07)** | **36.96** | **5.1** |  |
|  | low risk | 31 (79.49) | 10 (32.26) | nsꬸ | 63.81 | 14.83 | ns† ns◊ ns¥ | 1 (3.23) | 13 (41.94) | 17 (54.84) | nsꬸ | 5 (55.56) | 35.29 | 4.79 | ns† |
|  | indeterminate risk | 6 (15.38) | 0 (0) |  | 67.5 | 9.22 |  | 0 (0) | 2 (33.33) | 4 (66.67) |  | 4 (44.44) | 39.51 | 5.31 |  |
|  | high risk | 2 (5.13) | 0 (0) |  | 85.5 | 6.36 |  | 0 (0) | 0 (0) | 2 (100) |  | 0 (0) | na | na |  |
| **Nephrology & Hypertension** | **all** | **1,615 (4.44)** | **670 (41.49)** |  | **55.69** | **16.59** |  | **229 (14.18)** | **830 (51.39)** | **556 (34.43)** |  | **1383 (11.31)** | **39.49** | **11.09** |  |
|  | low risk | 1248 (77.28) | 537 (43.03) | <0.05ꬸ | 53.42 | 17.11 | <0.001† <0.001◊ <0.05¥ | 222 (17.79) | 617 (49.44) | 409 (32.77) | <0.001ꬸ | 1,058 (76.5) | 39.48 | 11.47 | ns† ns◊ ns¥ |
|  | indeterminate risk | 331 (20.50) | 125 (37.76) |  | 62.56 | 11.42 |  | 6 (1.81) | 205 (61.93) | 120 (36.25) |  | 296 (21.4) | 39.72 | 9.9 |  |
|  | high risk | 36 (2.23) | 8 (22.22) |  | 70.97 | 13.2 |  | 1 (2.78) | 8 (22.22) | 27 (75) |  | 29 (2.1) | 37.39 | 8.41 |  |
| **Secondary hospital I^†^** | **all** | **5152 (14.2)** | **2702 (52.45)** |  | **61.91** | **20.82** |  | **730 (14.17)** | **1809 (35.11)** | **2613 (50.72)** |  | **643 (5.25)** | **47.35** | **17.2** |  |
|  | low risk | 3303 (64.11) | 1761 (53.32) | nsꬸ | 55.56 | 20.99 | <0.001† <0.001◊ <0.001¥ | 704 (21.31) | 1271 (38.48) | 1328 (40.21) | <0.001ꬸ | 347 (53.97) | 48.86 | 19.59 | <0.05† ns◊ ns¥ |
|  | indeterminate risk | 1345 (26.11) | 694 (51.60) |  | 70.69 | 15.11 |  | 24 (1.78) | 489 (36.36) | 832 (61.86) |  | 213 (33.13) | 45.21 | 13.65 |  |
|  | high risk | 504 (9.78) | 247 (49.01) |  | 80.18 | 11.55 |  | 2 (0.4) | 49 (9.72) | 453 (89.88) |  | 83 (12.91) | 46.53 | 13.89 |  |
| **Secondary hospital II^‡^** | **all** | **3,646 (10.03)** | **1,938 (53.15)** |  | **61.05** | **21.51** |  | **590 (16.18)** | **1260 (34.56)** | **1796 (49.26)** |  | **505 (4.13)** | **46.96** | **17.62** |  |
|  | low risk | 2070 (56.77) | 1133 (54.73) | <0.05ꬸ | 52.49 | 21.11 | <0.001† <0.001◊ <0.001¥ | 549 (26.52) | 783 (37.83) | 738 (35.65) | <0.001ꬸ | 237 (46.93) | 46.92 | 18.81 | ns† ns◊ ns¥ |
|  | indeterminate risk | 1030 (28.25) | 543 (543) |  | 68.13 | 16.68 |  | 40 (3.88) | 423 (41.07) | 567 (55.05) |  | 165 (32.67) | 46.11 | 15.27 |  |
|  | high risk | 546 (14.98) | 262 (47.99) |  | 80.16 | 11.73 |  | 1 (0.18) | 54 (9.89) | 491 (89.93) |  | 103 (20.4) | 48.45 | 18.38 |  |
| **Secondary hospital  specialized in geriatrics^§^** | **all** | **168 (0.46)** | **93 (55.36)** |  | **77.57** | **13.93** |  | **3 (1.79)** | **22 (13.1)** | **143 (85.12)** |  | **40 (0.33)** | **43.7** | **12.34** |  |
|  | low risk | 96 (57.14) | 54 (56.25) | nsꬸ | 74.25 | 14.64 | <0.05† <0.05◊ ns¥ | 3 (3.13) | 13 (13.54) | 80 (83.33) | nsꬸ | 23 (57.5) | 43.3 | 11.75 | ns† ns◊ ns¥ |
|  | indeterminate risk | 49 (29.17) | 28 (57.14) |  | 80.12 | 12.45 |  | 0 (0) | 9 (18.37) | 40 (81.63) |  | 9 (22.5) | 47.17 | 12.89 |  |
|  | high risk | 23 (13.69) | 11 (47.83) |  | 85.96 | 8.67 |  | 0 (0) | 0 (0) | 23 (100) |  | 8 (20) | 40.97 | 14.19 |  |

**Table E.** (Continued.)

|  |  | **HbA1c range;  n (%)** | | | | **Total cholesterol;  N or n (%)** | **Total cholesterol,  mmol/L** | | | **Total cholesterol range;  n (%)** | | | |
| --- | --- | --- | --- | --- | --- | --- | --- | --- | --- | --- | --- | --- | --- |
|  |  | <39 mmol/mol  (normal) | 39-47 mmol/mol  (prediabetes) | ≥48 mmol/mol  (diabetes) | p-value |  | mean | SD | p-value | <5.2 mmol/L  (healthy level) | 5.2-6.19 mmol/L  (borderline high level) | ≥6.2 mmol/L  (high level) | p-value |
| **Overall** | **all** | **7,549 (61.73)** | **2,836 (23.19)** | **1,844 (15.08)** |  | **12,649** | **4.64** | **1.22** |  | **8,778 (69.40)** | **2,630 (20.79)** | **1,241 (9.81)** |  |
|  | low risk | 5482 (72.62) | 1826 (64.39) | 1156 (62.69) | <0.001ꬸ | 9,051 (71.56) | 4.73 | 1.2 | <0.001† <0.001◊ <0.001¥ | 6115 (69.66) | 1990 (75.67) | 946 (76.23) | <0.001ꬸ |
|  | indeterminate risk | 1,566 (20.74) | 765 (26.97) | 484 (26.25) |  | 2,804 (22.17) | 4.5 | 1.24 |  | 2,022 (23.03) | 540 (20.53) | 242 (19.5) |  |
|  | high risk | 501 (6.64) | 245 (8.64) | 204 (11.06) |  | 794 (6.28) | 4.15 | 1.28 |  | 641 (7.3) | 100 (3.8) | 53 (4.27) |  |
| **Angiology & Vascular surgery** | **all** | **11 (57.89)** | **8 (42.11)** | **0 (0)** |  | **19 (0.15)** | **4.79** | **1.36** |  | **11 (57.89)** | **5 (26.32)** | **3 (15.79)** |  |
|  | low risk | 9 (81.82) | 4 (50) | 0 (0) | nsꬸ | 13 (68.42) | 4.87 | 1.27 | ns† ns◊ ns¥ | 7 (63.64) | 4 (80) | 2 (66.67) | nsꬸ |
|  | indeterminate risk | 1 (9.09) | 3 (37.5) | 0 (0) |  | 4 (21.05) | 4.44 | 1.6 |  | 3 (27.27) | 1 (20) | 0 (0) |  |
|  | high risk | 1 (9.09) | 1 (12.5) | 0 (0) |  | 2 (10.53) | 4.98 | 2.43 |  | 1 (9.09) | 0 (0) | 1 (33.33) |  |
| **Cardiology** | **all** | **562 (53.99)** | **330 (31.7)** | **149 (14.31)** |  | **999 (7.9)** | **4.25** | **1.14** |  | **800 (80.08)** | **141 (14.11)** | **58 (5.81)** |  |
|  | low risk | 347 (61.74) | 201 (60.91) | 89 (59.73) | nsꬸ | 601 (60.16) | 4.36 | 1.18 | <0.05† <0.05◊ ns¥ | 465 (58.13) | 89 (63.12) | 47 (81.03) | <0.05ꬸ |
|  | indeterminate risk | 176 (31.32) | 104 (31.52) | 51 (34.23) |  | 325 (32.53) | 4.12 | 1.04 |  | 271 (33.88) | 45 (31.91) | 9 (15.52) |  |
|  | high risk | 39 (6.94) | 25 (7.58) | 9 (6.04) |  | 73 (7.31) | 3.9 | 1 |  | 64 (8) | 7 (4.96) | 2 (3.45) |  |
| **Neurology & Neurosurgery** | **all** | **420 (74.87)** | **110 (19.61)** | **31 (5.53)** |  | **155 (1.23)** | **4.85** | **1.13** |  | **99 (63.87)** | **37 (23.87)** | **19 (12.26)** |  |
|  | low risk | 359 (85.48) | 85 (77.27) | 26 (83.87) | <0.05ꬸ | 123 (79.35) | 4.92 | 1.12 | ns† ns◊ ns¥ | 76 (76.77) | 30 (81.08) | 17 (89.47) | nsꬸ |
|  | indeterminate risk | 59 (14.05) | 19 (17.27) | 4 (12.9) |  | 28 (18.06) | 4.72 | 1.15 |  | 19 (19.19) | 7 (18.92) | 2 (10.53) |  |
|  | high risk | 2 (0.48) | 6 (5.45) | 1 (3.23) |  | 4 (2.58) | 3.77 | 0.7 |  | 4 (4.04) | 0 (0) | 0 (0) |  |
| **Psychosomatic medicine** | **all** | **25 (55.56)** | **10 (22.22)** | **10 (22.22)** |  | **25 (0.2)** | **5.29** | **1.33** |  | **14 (56)** | **5 (20)** | **6 (24)** |  |
|  | low risk | 21 (84) | 7 (70) | 10 (100) | nsꬸ | 21 (84) | 5.37 | 1.37 | ns† | 11 (78.57) | 4 (80) | 6 (100) | nsꬸ |
|  | indeterminate risk | 4 (16) | 2 (20) | 0 (0) |  | 4 (16) | 4.84 | 1.16 |  | 3 (21.43) | 1 (20) | 0 (0) |  |
|  | high risk | 0 (0) | 1 (10) | 0 (0) |  | 0 (0) | na | na |  | 0 (0) | 0 (0) | 0 (0) |  |
| **Ophthalmology** | **all** | **8 (47.06)** | **5 (29.41)** | **4 (23.53)** |  | **12 (0.09)** | **4.83** | **1.06** |  | **8 (66.67)** | **3 (25)** | **1 (8.33)** |  |
|  | low risk | 4 (50) | 3 (60) | 2 (50) | nsꬸ | 7 (58.33) | 5.12 | 1.14 | ns† ns◊ ns¥ | 4 (50) | 2 (66.67) | 1 (100) | nsꬸ |
|  | indeterminate risk | 3 (37.5) | 2 (40) | 2 (50) |  | 4 (33.33) | 4.64 | 0.85 |  | 3 (37.5) | 1 (33.33) | 0 (0) |  |
|  | high risk | 1 (12.5) | 0 (0) | 0 (0) |  | 1 (8.33) | 3.54 |  |  | 1 (12.5) | 0 (0) | 0 (0) |  |
| **Obstetrics and Gynaecology** | **all** | **424 (88.15)** | **40 (8.32)** | **17 (3.53)** |  | **741 (5.86)** | **5.11** | **1** |  | **409 (55.2)** | **237 (31.98)** | **95 (12.82)** |  |
|  | low risk | 371 (87.5) | 36 (90) | 14 (82.35) | nsꬸ | 655 (88.39) | 5.08 | 0.99 | ns† ns◊ ns¥ | 371 (90.71) | 207 (87.34) | 77 (81.05) | nsꬸ |
|  | indeterminate risk | 49 (11.56) | 4 (10) | 2 (11.76) |  | 81 (10.93) | 5.34 | 1.09 |  | 36 (8.8) | 28 (11.81) | 17 (17.89) |  |
|  | high risk | 4 (0.94) | 0 (0) | 1 (5.88) |  | 5 (0.67) | 5.39 | 1.05 |  | 2 (0.49) | 2 (0.84) | 1 (1.05) |  |
| **Anesthesiology and  Pain management** | **all** | **4 (66.67)** | **1 (16.67)** | **1 (16.67)** |  | **6 (0.05)** | **4.63** | **0.81** |  | **5 (83.33)** | **1 (16.67)** | **0 (0)** |  |
|  | low risk | 1 (25) | 1 (100) | 0 (0) | nsꬸ | 2 (33.33) | 4.79 | 0.27 | ns† ns◊ ns¥ | 2 (40) | 0 (0) | 0 (0) | nsꬸ |
|  | indeterminate risk | 2 (50) | 0 (0) | 0 (0) |  | 2 (33.33) | 3.88 | 0.36 |  | 2 (40) | 0 (0) | 0 (0) |  |
|  | high risk | 1 (25) | 0 (0) | 1 (100) |  | 2 (33.33) | 5.21 | 1.12 |  | 1 (20) | 1 (100) | 0 (0) |  |
| **Infectiology** | **all** | **138 (61.61)** | **58 (25.89)** | **28 (12.5)** |  | **797 (6.3)** | **5** | **1.13** |  | **467 (58.59)** | **218 (27.35)** | **112 (14.05)** |  |
|  | low risk | 87 (63.04) | 40 (68.97) | 17 (60.71) | nsꬸ | 548 (68.76) | 5.04 | 1.12 | ns† ns◊ ns¥ | 316 (67.67) | 155 (71.1) | 77 (68.75) | nsꬸ |
|  | indeterminate risk | 46 (33.33) | 18 (31.03) | 9 (32.14) |  | 228 (28.61) | 4.94 | 1.13 |  | 138 (29.55) | 57 (26.15) | 33 (29.46) |  |
|  | high risk | 5 (3.62) | 0 (0) | 2 (7.14) |  | 21 (2.63) | 4.64 | 1.25 |  | 13 (2.78) | 6 (2.75) | 2 (1.79) |  |
| **Emergency department** | **all** | **678 (52.19)** | **359 (27.64)** | **262 (20.17)** |  | **1112 (8.79)** | **4.35** | **1.28** |  | **827 (74.37)** | **195 (17.54)** | **90 (8.09)** |  |
|  | low risk | 376 (55.46) | 173 (48.19) | 150 (57.25) | nsꬸ | 601 (54.05) | 4.57 | 1.28 | <0.001† <0.001◊ ns¥ | 417 (50.42) | 116 (59.49) | 68 (75.56) | <0.001ꬸ |
|  | indeterminate risk | 186 (27.43) | 116 (32.31) | 67 (25.57) |  | 315 (28.33) | 4.19 | 1.19 |  | 247 (29.87) | 54 (27.69) | 14 (15.56) |  |
|  | high risk | 116 (17.11) | 70 (19.5) | 45 (17.18) |  | 196 (17.63) | 3.96 | 1.31 |  | 163 (19.71) | 25 (12.82) | 8 (8.89) |  |
| **Medical and radio oncology  & Nuclear medicine** | **all** | **317 (61.08)** | **131 (25.24)** | **71 (13.68)** |  | **574 (4.54)** | **4.72** | **1.29** |  | **385 (67.07)** | **136 (23.69)** | **53 (9.23)** |  |
|  | low risk | 213 (67.19) | 97 (74.05) | 58 (81.69) | nsꬸ | 414 (72.13) | 4.84 | 1.31 | <0.05† <0.05◊ ns¥ | 269 (69.87) | 103 (75.74) | 42 (79.25) | nsꬸ |
|  | indeterminate risk | 76 (23.97) | 25 (19.08) | 7 (9.86) |  | 120 (20.91) | 4.5 | 1.14 |  | 85 (22.08) | 27 (19.85) | 8 (15.09) |  |
|  | high risk | 28 (8.83) | 9 (6.87) | 6 (8.45) |  | 40 (6.97) | 4.17 | 1.28 |  | 31 (8.05) | 6 (4.41) | 3 (5.66) |  |
| **Otorhinolaryngology** | **all** | **2 (66.67)** | **1 (33.33)** | **0 (0)** |  | **1 (0.01)** | **8.72** | **na** |  | **0 (0)** | **0 (0)** | **1 (100)** |  |
|  | low risk | 2 (100) | 1 (100) | 0 (0) | naꬸ | 3 (100) | 8.72 | na | na | 0 (0) | 0 (0) | 1 (100) | naꬸ |
|  | indeterminate risk | 0 (0) | 0 (0) | 0 (0) |  | 0 (0) | na | na |  | 0 (0) | 0 (0) | 0 (0) |  |
|  | high risk | 0 (0) | 0 (0) | 0 (0) |  | 0 (0) | na | na |  | 0 (0) | 0 (0) | 0 (0) |  |
| **Orthopaedic surgery** | **all** | **4 (50)** | **4 (50)** | **8 (100)** |  | **8 (0.06)** | **4.79** | **0.94** |  | **6 (75)** | **1 (12.5)** | **1 (12.5)** |  |
|  | low risk | 3 (75) | 2 (50) | 5 (62.5) | nsꬸ | 5 (62.5) | 4.96 | 1.13 | ns† | 3 (50) | 1 (100) | 1 (100) | nsꬸ |
|  | indeterminate risk | 1 (25) | 2 (50) | 3 (37.5) |  | 3 (37.5) | 4.51 | 0.57 |  | 3 (50) | 0 (0) | 0 (0) |  |
|  | high risk | 0 (0) | 0 (0) | 0 (0) |  | 0 (0) | na | na |  | 0 (0) | 0 (0) | 0 (0) |  |
| **Osteoporosis** | **all** | **4 (80)** | **0 (0)** | **1 (20)** |  | **4 (0.03)** | **4.99** | **1.04** |  | **2 (50)** | **2 (50)** | **0 (0)** |  |
|  | low risk | 3 (75) | 0 (0) | 1 (100) | nsꬸ | 3 (75) | 4.59 | 0.82 | ns† | 2 (100) | 1 (50) | 0 (0) | nsꬸ |
|  | indeterminate risk | 1 (25) | 0 (0) | 0 (0) |  | 1 (25) | 6.18 | na |  | 0 (0) | 1 (50) | 0 (0) |  |
|  | high risk | 0 (0) | 0 (0) | 0 (0) |  | na | na | na |  | 0 (0) | 0 (0) | 0 (0) |  |
| **General internal medicine** | **all** | **294 (65.33)** | **108 (24)** | **48 (10.67)** |  | **434 (3.43)** | **5.09** | **1.26** |  | **239 (55.07)** | **129 (29.72)** | **66 (15.21)** |  |
|  | low risk | 239 (81.29) | 82 (75.93) | 32 (66.67) | <0.05ꬸ | 346 (79.72) | 5.16 | 1.19 | ns† ns◊ ns¥ | 183 (76.57) | 107 (82.95) | 56 (84.85) | nsꬸ |
|  | indeterminate risk | 43 (14.63) | 24 (22.22) | 15 (31.25) |  | 75 (17.28) | 4.81 | 1.52 |  | 47 (19.67) | 19 (14.73) | 9 (13.64) |  |
|  | high risk | 12 (4.08) | 2 (1.85) | 1 (2.08) |  | 13 (3) | 4.56 | 1.14 |  | 9 (3.77) | 3 (2.33) | 1 (1.52) |  |
| **Hematology and Central  hematological Laboratory** | **all** | **174 (72.5)** | **43 (17.92)** | **23 (9.58)** |  | **248 (1.96)** | **4.43** | **1.17** |  | **188 (75.81)** | **41 (16.53)** | **19 (7.66)** |  |
|  | low risk | 125 (71.84) | 27 (62.79) | 14 (60.87) | nsꬸ | 166 (66.94) | 4.53 | 1.15 | ns† ns◊ ns¥ | 121 (64.36) | 32 (78.05) | 13 (68.42) | nsꬸ |
|  | indeterminate risk | 31 (17.82) | 14 (32.56) | 5 (21.74) |  | 51 (20.56) | 4.34 | 1.12 |  | 40 (21.28) | 8 (19.51) | 3 (15.79) |  |
|  | high risk | 18 (10.34) | 2 (4.65) | 4 (17.39) |  | 31 (12.5) | 4.05 | 1.25 |  | 27 (14.36) | 1 (2.44) | 3 (15.79) |  |
| **Rheumatology & Immunology** | **all** | **234 (66.67)** | **73 (20.8)** | **44 (12.54)** |  | **305 (2.41)** | **5.15** | **1.29** |  | **169 (55.41)** | **66 (21.64)** | **70 (22.95)** |  |
|  | low risk | 190 (81.2) | 57 (78.08) | 34 (77.27) | nsꬸ | 244 (80) | 5.15 | 1.29 | ns† ns◊ ns¥ | 136 (80.47) | 50 (75.76) | 58 (82.86) | nsꬸ |
|  | indeterminate risk | 40 (17.09) | 13 (17.81) | 10 (22.73) |  | 54 (17.7) | 5.11 | 1.33 |  | 30 (17.75) | 14 (21.21) | 10 (14.29) |  |
|  | high risk | 4 (1.71) | 3 (4.11) | 0 (0) |  | 7 (2.3) | 5.3 | 1.13 |  | 3 (1.78) | 2 (3.03) | 2 (2.86) |  |
| **Pneumology & Allergology** | **all** | **72 (51.8)** | **41 (29.5)** | **26 (18.71)** |  | **135 (1.07)** | **4.46** | **1.16** |  | **101 (74.81)** | **23 (17.04)** | **11 (8.15)** |  |
|  | low risk | 52 (72.22) | 26 (63.41) | 16 (61.54) | nsꬸ | 92 (68.15) | 4.58 | 1.13 | ns† ns◊ ns¥ | 68 (67.33) | 15 (65.22) | 9 (81.82) | nsꬸ |
|  | indeterminate risk | 15 (20.83) | 15 (36.59) | 10 (38.46) |  | 40 (29.63) | 4.26 | 1.22 |  | 30 (29.7) | 8 (34.78) | 2 (18.18) |  |
|  | high risk | 5 (6.94) | 0 (0) | 0 (0) |  | 3 (2.22) | 3.38 | 0.61 |  | 3 (2.97) | 0 (0) | 0 (0) |  |
| **Thoracic surgery** | **all** | **23 (69.7)** | **8 (24.24)** | **2 (6.06)** |  | **35 (0.28)** | **4.57** | **1.11** |  | **27 (77.14)** | **4 (11.43)** | **4 (11.43)** |  |
|  | low risk | 19 (82.61) | 5 (62.5) | 1 (50) | nsꬸ | 29 (82.86) | 4.51 | 1.09 | ns† ns◊ ns¥ | 23 (85.19) | 3 (75) | 3 (75) | nsꬸ |
|  | indeterminate risk | 4 (17.39) | 2 (25) | 1 (50) |  | 5 (14.29) | 5.12 | 1.24 |  | 3 (11.11) | 1 (25) | 1 (25) |  |
|  | high risk | 0 (0) | 1 (12.5) | 0 (0) |  | 1 (2.86) | 3.5 | 0 |  | 1 (3.7) | 0 (0) | 0 (0) |  |
| **Dermatology & Venereology** | **all** | **150 (59.76)** | **58 (23.11)** | **43 (17.13)** |  | **293 (2.32)** | **4.92** | **1.09** |  | **178 (60.75)** | **76 (25.94)** | **39 (13.31)** |  |
|  | low risk | 115 (76.67) | 47 (81.03) | 32 (74.42) | nsꬸ | 245 (83.62) | 4.92 | 1.04 | ns† ns◊ ns¥ | 150 (84.27) | 63 (82.89) | 32 (82.05) | nsꬸ |
|  | indeterminate risk | 29 (19.33) | 11 (18.97) | 10 (23.26) |  | 44 (15.02) | 5.02 | 1.37 |  | 24 (13.48) | 13 (17.11) | 7 (17.95) |  |
|  | high risk | 6 (4) | 0 (0) | 1 (2.33) |  | 4 (1.37) | 4.2 | 0.41 |  | 4 (2.25) | 0 (0) | 0 (0) |  |
| **Diabetology, Endocrinology,  Nutritional medicine  & Metabolism** | **all** | **1148 (70.52)** | **261 (16.03)** | **219 (13.45)** |  | **2304 (18.21)** | **4.52** | **1.14** |  | **1708 (74.13)** | **424 (18.4)** | **172 (7.47)** |  |
|  | low risk | 1032 (89.9) | 231 (88.51) | 172 (78.54) | <0.001ꬸ | 2,016 (87.5) | 4.55 | 1.13 | <0.05† ns◊ ns¥ | 1484 (86.89) | 382 (90.09) | 150 (87.21) | nsꬸ |
|  | indeterminate risk | 109 (9.49) | 30 (11.49) | 42 (19.18) |  | 269 (11.68) | 4.28 | 1.16 |  | 209 (12.24) | 41 (9.67) | 19 (11.05) |  |
|  | high risk | 7 (0.61) | 0 (0) | 5 (2.28) |  | 19 (0.82) | 4.28 | 1.39 |  | 15 (0.88) | 1 (0.24) | 3 (1.74) |  |
| **Visceral surgery and medicine** | **all** | **1543 (66.25)** | **485 (20.82)** | **301 (12.92)** |  | **2236 (17.68)** | **4.68** | **1.18** |  | **1564 (69.95)** | **462 (20.66)** | **210 (9.39)** |  |
|  | low risk | 1004 (65.07) | 284 (58.56) | 145 (48.17) | <0.001ꬸ | 1,408 (62.97) | 4.75 | 1.12 | ns† <0.001◊ <0.001¥ | 966 (61.76) | 305 (66.02) | 137 (65.24) | <0.05ꬸ |
|  | indeterminate risk | 372 (24.11) | 142 (29.28) | 100 (33.22) |  | 588 (26.3) | 4.69 | 1.23 |  | 401 (25.64) | 128 (27.71) | 59 (28.1) |  |
|  | high risk | 167 (10.82) | 59 (12.16) | 56 (18.6) |  | 240 (10.73) | 4.19 | 1.26 |  | 197 (12.6) | 29 (6.28) | 14 (6.67) |  |
| **Urology** | **all** | **5 (55.56)** | **4 (44.44)** | **0 (0)** |  | **11 (0.09)** | **4.63** | **1.35** |  | **7 (63.64)** | **2 (18.18)** | **2 (18.18)** |  |
|  | low risk | 3 (60) | 2 (50) | 0 (0) | nsꬸ | 8 (72.72) | 4.65 | 1.33 | ns† | 5 (71.43) | 2 (100) | 1 (50) | nsꬸ |
|  | indeterminate risk | 2 (40) | 2 (50) | 0 (0) |  | 3 (27.27) | 4.58 | 1.7 |  | 2 (28.57) | 0 (0) | 1 (50) |  |
|  | high risk | 0 (0) | 0 (0) | 0 (0) |  | 0 (0) | na | na |  | 0 (0) | 0 (0) | 0 (0) |  |
| **Nephrology & Hypertension** | **all** | **870 (62.91)** | **323 (23.36)** | **190 (13.74)** |  | **1351 (10.68)** | **4.62** | **1.26** |  | **950 (70.32)** | **270 (19.99)** | **131 (9.7)** |  |
|  | low risk | 672 (77.24) | 241 (74.61) | 145 (76.32) | nsꬸ | 1,039 (76.91) | 4.68 | 1.25 | <0.05† ns◊ ns¥ | 714 (75.16) | 221 (81.85) | 104 (79.39) | nsꬸ |
|  | indeterminate risk | 181 (20.8) | 73 (22.6) | 42 (22.11) |  | 284 (21.02) | 4.44 | 1.25 |  | 214 (22.53) | 45 (16.67) | 25 (19.08) |  |
|  | high risk | 17 (1.95) | 9 (2.79) | 3 (1.58) |  | 28 (2.07) | 4.15 | 1.14 |  | 22 (2.32) | 4 (1.48) | 2 (1.53) |  |
| **Secondary hospital I^†^** | **all** | **227 (35.3)** | **205 (31.9)** | **211 (32.81)** |  | **478 (3.78)** | **4.52** | **1.27** |  | **349 (73.01)** | **85 (17.78)** | **44 (9.21)** |  |
|  | low risk | 122 (53.74) | 107 (52.2) | 118 (55.92) | nsꬸ | 285 (59.62) | 4.62 | 1.26 | ns† ns◊ ns¥ | 200 (57.31) | 58 (68.24) | 27 (61.36) | nsꬸ |
|  | indeterminate risk | 78 (34.36) | 75 (36.59) | 60 (28.44) |  | 150 (31.38) | 4.38 | 1.25 |  | 116 (33.24) | 21 (24.71) | 13 (29.55) |  |
|  | high risk | 27 (11.89) | 23 (11.22) | 33 (15.64) |  | 43 (9) | 4.37 | 1.35 |  | 33 (9.46) | 6 (7.06) | 4 (9.09) |  |
| **Secondary hospital II^‡^** | **all** | **193 (38.22)** | **158 (31.29)** | **154 (30.5)** |  | **347 (2.74)** | **4.42** | **1.36** |  | **249 (71.76)** | **66 (19.02)** | **32 (9.22)** |  |
|  | low risk | 101 (52.33) | 61 (38.61) | 75 (48.7) | nsꬸ | 169 (48.7) | 4.53 | 1.26 | ns† ns◊ ns¥ | 115 (46.18) | 39 (59.09) | 15 (46.88) | nsꬸ |
|  | indeterminate risk | 56 (29.02) | 65 (41.14) | 44 (28.57) |  | 120 (34.58) | 4.3 | 1.36 |  | 90 (36.14) | 20 (30.3) | 10 (31.25) |  |
|  | high risk | 36 (18.65) | 32 (20.25) | 35 (22.73) |  | 58 (16.71) | 4.34 | 1.62 |  | 44 (17.67) | 7 (10.61) | 7 (21.88) |  |
| **Secondary hospital  specialized in geriatrics^§^** | **all** | **19 (47.5)** | **12 (30)** | **9 (22.5)** |  | **19 (0.15)** | **4.08** | **1.2** |  | **16 (84.21))** | **1 (5.26)** | **2 (110.52)** |  |
|  | low risk | 12 (63.16) | 6 (50) | 5 (55.56) | nsꬸ | 10 (52.63) | 4.65 | 1.36 | ns† ns◊ ns¥ | 7 (43.75) | 1 (100) | 2 (100) | nsꬸ |
|  | indeterminate risk | 2 (10.53) | 4 (33.33) | 3 (33.33) |  | 6 (31.58) | 3.5 | 0.66 |  | 6 (37.5) | 0 (0) | 0 (0) |  |
|  | high risk | 5 (26.32) | 2 (16.67) | 1 (11.11) |  | 3 (15.79) | 3.36 | 0.42 |  | 3 (18.75) | 0 (0) | 0 (0) |  |

**SD, standard deviation; ASAT, aspartate aminotransferase; ALAT, alanine aminotransferase; HbA1c, glycosylated hemoglobin; ns, not significant; na, not applicable.**

Analysis of variance (ANOVA) and Bonferroni posthoc: † p low risk vs. intermediate risk of ≥ F3 fibrosis; ◊ p low risk vs. high risk of ≥ F3 fibrosis; ¥ intermediate risk vs. high risk of ≥ F3 fibrosis.

ꬸChi-square test.

Low risk of ≥ F3 fibrosis: <1.3 (<65 years) and <2.0 (≥65 years); Intermediate risk of ≥ F3 fibrosis: ≥1.3 to ≤3.25 (<65 years) ≥2.0 to ≤3.25 (≥65 years); High risk of ≥ F3 fibrosis: >3.25.

^†^encompasses heterogenous patient cohorts from the hospitals Aarberg, Riggisberg and Münsingen.

‡encompasses heterogenous patient cohorts from the hospital Tiefenau.

§encompasses the nursing home Frienisberg and Seelandheim.

# 8. **Table F.** Comparison of risk stratification for advanced fibrosis by FIB-4 score in hematological settings.

|  |  |  | **FIB-4 score** | | | | | |
| --- | --- | --- | --- | --- | --- | --- | --- | --- |
|  |  |  | Lower cutoff: **<1.3** (<65 years); **<2.0** (≥65 years) Upper cutoff: **>2.67** | | | Lower cutoff: **<1.3** (<65 years); **<2.0** (≥65 years) Upper cutoff: **>3.25** | | |
|  |  | N or n (%) | Low risk  of ≥ F3 fibrosis | Indeterminate risk  of ≥ F3 fibrosis | High risk  of ≥ F3 fibrosis | Low risk  of ≥ F3 fibrosis | Indeterminate risk  of ≥ F3 fibrosis | High risk  of ≥ F3 fibrosis |
| **Overall** | **original data†** | **36,360** | **26,245 (72.18)** | **6,202 (17.06)** | **3,913 (10.76)** | **26,245 (72.18)** | **7,518 (20.68)** | **2,597 (7.14 )** |
|  | **excluded data^§^** | **33,722** | **25,991 (77.07)** | **5,503 (16.32)** | **2,228 (6.61)** | **25,991 (77.07)** | **6,504 (19.29)** | **1,227 (3.64 )** |
| Medicine^‡^ | original data† | 14877 (40.92) | 11892 (79.94) | 2219 (14.92) | 766 (5.15) | 11892 (79.94) | 2530 (17.01) | 455 (3.06) |
|  | excluded data^§^ | 13,953 (41.37) | 11,776 (84.40) | 1,888 (13.53) | 289 (2.07) | 11,776 (84.40) | 2,077 (14.89) | 100 (0.72) |
| Hematology and Central  hematological Laboratory | original data† | 1,139 (3.13) | 817 (71.73) | 167 (14.66) | 155 (13.61) | 817 (71.73) | 217 (19.05) | 105 (9.22) |
|  | excluded data^§^ | 954 (2.82) | 797 (83.54) | 122 (12.79) | 35 (3.67) | 797 (83.54) | 148 (15.51) | 9 (0.94) |

**FIB-4, Fibrosis-4; F3, advanced fibrosis.**

^†^dataset included patients with platelet counts < 150 G/L.

^§^dataset excluded patients with platelet counts < 150 G/L.

^‡^encompasses the following clinics: Ophthalmology, Infectiology, Hematology and Central hematological Laboratory, Pneumology & Allergology, Medical and radio oncology & Nuclear medicine, Anesthesiology and Pain management, Nephrology & Hypertension, Urology, Otorhinolaryngology, Osteoporosis, General internal medicine, Dermatology & Venereology, Rheumatology & Immunology, Neurology & Neurosurgery and Obstetrics and Gynaecology.

# 9. **Text A.** The structure of the Swiss health care system and the “Insel Gruppe” in Switzerland in the year of 2022.

The Swiss healthcare system is organized into different levels of care (primary, secondary and tertiary care) with increasing levels of specialization. Primary care is typically provided by general practitioners. Secondary care consists of a more specialized services (e.g. provided at hospitals) that offer a broader range of specialties but do not have the same level of specialized care as tertiary care centers (e.g. academic hospitals or university-affiliated centers).

In the year of 2022 the regional networks called “Inselgruppe” in Switzerland encompassesed the Inselspital, University Hospital Bern, four regional hospitals (the hospitals Aarberg, Riggisberg and Münsingen and Tiefenau) and two nursing homes (Frienisberg and Seelandheim).
